# Genome-Wide Screening of Genes Regulated by DNA Methylation in Colon Cancer Development

**DOI:** 10.1371/journal.pone.0046215

**Published:** 2012-10-01

**Authors:** Sándor Spisák, Alexandra Kalmár, Orsolya Galamb, Barna Wichmann, Ferenc Sipos, Bálint Péterfia, István Csabai, Ilona Kovalszky, Szabolcs Semsey, Zsolt Tulassay, Béla Molnár

**Affiliations:** 1 Molecular Medicine Research Unit, Hungarian Academy of Sciences, Budapest, Hungary; 2 2nd Department of Internal Medicine, Semmelweis University, Budapest, Hungary; 3 1st Department of Pathology and Experimental Cancer Research, Semmelweis University, Budapest, Hungary; 4 Department of Physics of Complex Systems, Eötvös University, Budapest, Hungary; 5 Department of Physics and Astronomy, The Johns Hopkins University, Baltimore, Maryland, United States of America; 6 Center for Models of Life, Niels Bohr Institute, Copenhagen, Denmark; Dartmouth Medical School, United States of America

## Abstract

Tumorigenesis is accompanied by changes in the DNA methylation pattern. Our aim was to test a novel approach for identification of transcripts at whole transcript level which are regulated by DNA methylation. Our approach is based on comparison of data obtained from transcriptome profiling of primary human samples and in vitro cell culture models. Epithelial cells were collected by LCM from normal, adenoma, and tumorous colonic samples. Using gene expression analysis, we identified downregulated genes in the tumors compared to normal tissues. In parallel 3000 upregulated genes were determined in HT-29 colon adenocarcinoma cell culture model after DNA demethylation treatment. Of the 2533 transcripts showing reduced expression in the tumorous samples, 154 had increased expression as a result of DNA demethylation treatment. Approximately 2/3 of these genes had decreased expression already in the adenoma samples. Expression of five genes (*GCG*, *NMES-1*, *LRMP*, *FAM161B* and *PTGDR*), was validated using RT-PCR. *PTGDR* showed ambiguous results, therefore it was further studied to verify the extent of DNA methylation and its effect on the protein level. Results confirmed that our approach is suitable for genome-wide screening of genes which are regulated or inactivated by DNA methylation. Activity of these genes possibly interferes with tumor progression, therefore genes identified can be key factors in the formation and in the progression of the disease.

## Introduction

Changes of gene expression, including activation of oncogenes and inactivation of tumor suppressors, are responsible for formation and development of colorectal cancer (CRC) [1], [2]. Beside the accumulating changes in the DNA sequence, the dysfunction of the epigenetic regulation system can also lead to aberrant formation of the colon epithelia along the progressive process of carcinogenesis [3], [4]. However, it is not clear which molecular events affect individual gene activities and whether it is a direct or an indirect effect [5], [6], [7].

One of the epigenetic processes influencing gene expression is DNA methylation, a post-replicative DNA modification that occurs predominantly in the genome regions rich in CG dinucleotides, so-called CpG islands [8]. Modification of bases by addition of a methyl group can physically inhibit binding of transcription factors, and also permits recruitment of the methyl-CpG-binding domain proteins (eg.: MDB1-3, MeCP2) to promoter regions, which can repress transcription initiation [9]. Aberrant changes of the tissue-specific methylation pattern are frequently manifested in two ways: (i) global hypomethylation, which occurs in the whole genome with aging, and (ii) local hypermethylation of 5′ regulatory regions in tumorigenesis, which usually leads to decreased or ceased transcriptional activity of the affected genes [10], [11], [12]. Hypermethylation mediated changes in gene regulation play a key role during development of several tumor types, including colorectal cancer, and also show a tumor-specific pattern [13].

It is well known, that DNA methylation affects gene activities without changing the DNA sequence itself, and can be reverted by demethylating agents which act by inhibiting DNA methylation, such as 5-aza-2′-deoxycytidine (5-Aza). This gives a theoretical possibility to decelerate or to arrest tumor development in case of early detection. However, for the better understanding of the DNA methylation processes, and for the possibility of using colorectal cancer-specific methylation biomarkers for screening patients, or even to achieve gene-targeted demethylation in the future, the affected genes need to be identified. Although several methylation-regulated genes have been reported to be associated with cancer (including colorectal cancer) development, a detailed process still remains unclear [14], [15], [16], [17], [18], [19].

Although many strategies are available for assessing DNA methylation at the whole genome level including sequencing [20], [21] and array systems [22], [23], these approaches can be sufficient only in case of tissues or cell cultures, from which relatively large amount of starting material (genomic DNA) can be obtained, as different cell types have distinct methylation patterns [24], [25], [26]. Laser capture microdissection (LCM) can serve as an adequate cell separating method [27]. However, the limited amount of the collectable specimen results in a challenging disadvantage in case of methylation studies, because the currently available in vitro amplification techniques cannot conserve the methylation pattern. In contrast, gene expression can be analyzed routinely on laser microdissected cells, which can be combined with microarray analysis to gather information even from a single cell at the whole genome level.

In this paper we present a gene expression based approach which is suitable for efficient, high-throughput, genome-wide screening for methylation-regulated genes, whose reduced expression may be related to cancer progression. The potential of this method was demonstrated previously by identifying 17 transcripts which were downregulated during colorectal cancer progression, and showed increased activity in HT-29 colorectal cancer cells after 5-Aza treatment [28]. Extending the previous study, here we report 154 genes, which are likely inhibited by methylation during the progression of colorectal cancer. The reliability of the method is supported by a wide range of experimental and *in silico* methods presented in this paper. Expression of several transcripts was validated by RT-PCR. We have revealed the relationship between the gene expression and methylation status in case of the *PTGDR* gene by RT-PCR analysis, immunohistochemistry, bisulfite sequencing, and HRM analysis. In PCA analyses normal, adenoma, and CRC samples could be successfully separated based on the expression patterns of these 154 transcripts, both in our own sample set and in independent sample sets obtained from the GEO database.

## Results

### Microarrays of LCM Tissue Samples and HT-29 Cells

**Figure 1:**
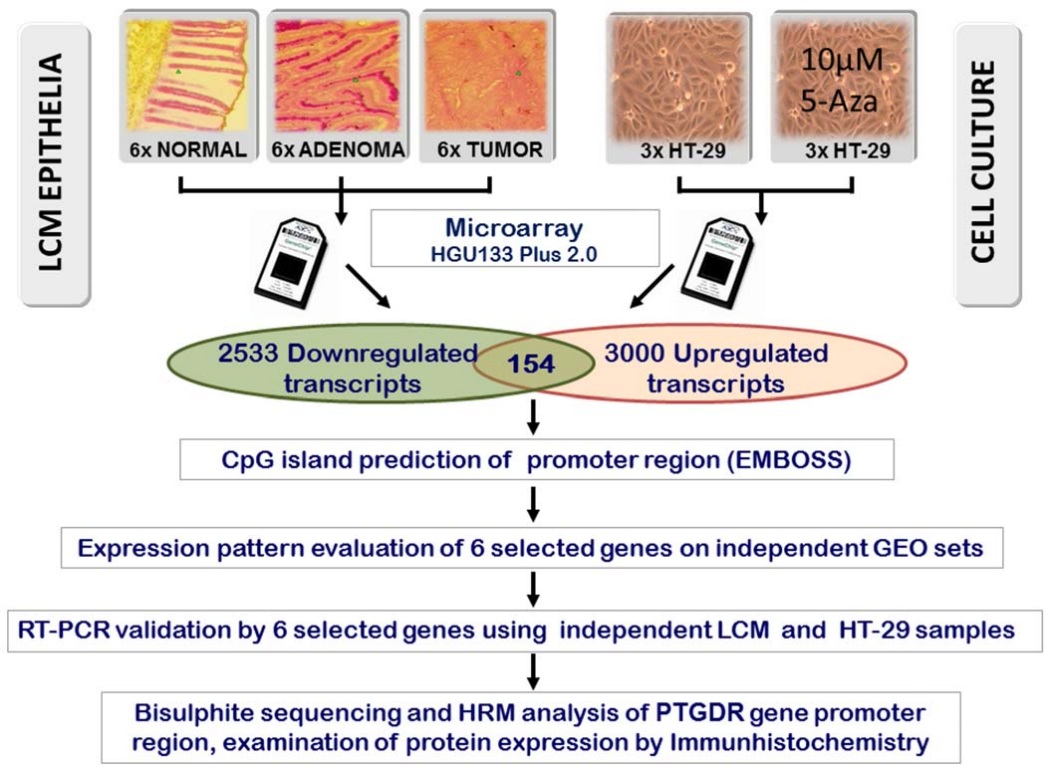
Experimental design.

Hypermethylation of several genomic regions were previously proven to be associated with oncogenic transformation. Because methylation of the CpG island(s) in a gene’s promoter region can reduce transcription of the gene, we searched for genes with decreasing expression in human colon tumor samples compared to normal colonic epithelial tissues. Expression changes in tumor samples can result from different molecular events including the direct and indirect effects of mutations [29], [30], [31]. However, changes in the expression pattern observed in a demethylated cell culture model can predict which genes are regulated by DNA methylation (Figure 1). Gene expression in tissue samples was studied by HGU133 Plus2.0 microarrays, and the obtained data were analyzed by the SAM (Significance Analysis of Microarrays) algorithm. About 2000 transcripts, belonging to approximately 2500 probe sets, were identified which had decreased expression in the tumor samples (Table S1).

**Figure 2:**
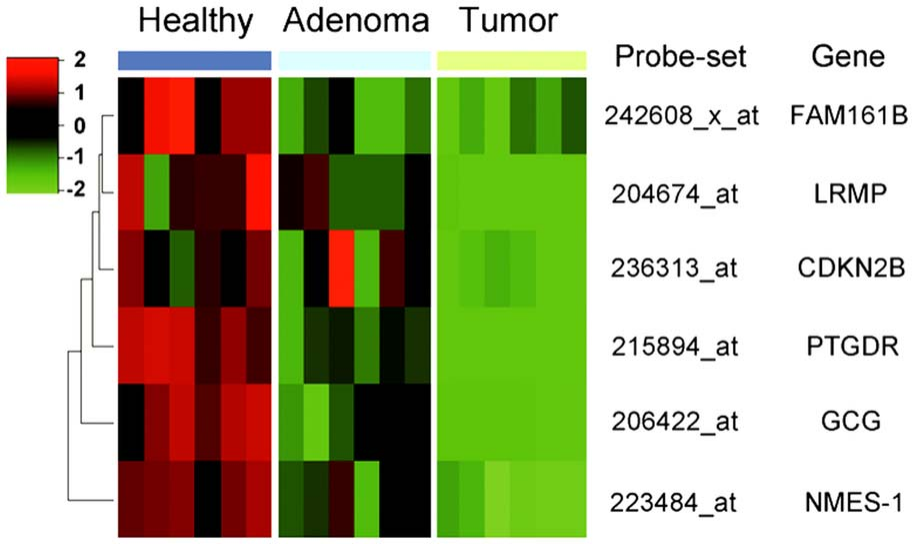
Differential expression of six selected genes in normal colonic epithelial samples, adenoma, and tumor samples. Expression of the tumor suppressor gene *CDKN2B* is shown for comparison. Probe set identification codes and gene names are indicated on the right. The six columns indicate microarray results obtained from six individual samples for each type of tissues. Black squares indicate that gene expression was unchanged in that experiment. The degree of intensity of red or green indicates the level of gene expression high or low, respectively.

Genes which are inactivated by hypermethylation can be reactivated by the removal of the methyl groups from the CpG islands of their promoter regions, which can be obtained by growing the cells in the presence of the DNA methyltransferase inhibitor 5-aza-2′-deoxycytidine. Gene expression levels in 5-Aza-treated and non-treated HT-29 colon adenocarcinoma cells were compared. Because 5-Aza treatment causes dose-dependent inhibition of cell proliferation [32], [33], [34], MTT assays were used to optimize the treatment concentration. Based on the results 10 µM 5-Aza was applied in the demethylation treatment of HT-29 cells. Because the 5-Aza treatment may not reactivate genes completely, we considered the top 3000 probe sets having the highest log_2_FC values at the significance level of p<0.025 to be potentially upregulated. Among the about 2000 transcripts that showed reduced transcription in the colon tumors, 154 genes were found which showed increased expression in the 5-Aza-treated HT-29 cells using the above criteria. Based on the microarray analysis these are candidate genes which are likely silenced in colorectal tumors by DNA hypermethylation either directly or indirectly (Table S2). Interestingly, 108 of the 154 transcripts had significantly decreased levels already in the adenoma samples (Figure S1) (Table S3). Promoter region prediction was performed using the EMBOSS CpG Plot tools. Based on the prediction results and analyses of independent public microarray data, 6 transcripts were selected for further analysis. These transcripts belong to the *GCG* (glucagon), *NMES-1* (normal mucosa of esophagus-specific 1, also called *C15orf48*), *LRMP* (lymphoid-restricted membrane protein), *FAM161B* (family with sequence similarity 161, member B), and *PTGDR* (prostaglandin D2 receptor) genes, which also showed differential expression in our previous pilot study [35], but have not been linked to CRC before, and to *CDKN2B*, a known tumor suppressor gene. Figure 2 represents the expression patterns of this set of 6 genes on our LCM data sets. The heatmap shows relatively high expression of the gene set in normal tissues which decreases during carcinogenesis.

### RT-PCR Validation Assays

In the microarray analyses transcription of all of these genes was inhibited in the tumor samples but showed different degrees of heterogeneity in the adenoma samples (Figure 2). Furthermore, these genes were reactivated to different extents as a result of 5-Aza treatment in HT-29 cells. In the microarray analysis GCG, NMES-1, and LRMP transcripts showed strong responses (>2.5-fold increase), while FAM161B and PTGDR mRNAs showed weaker responses (<1.5-fold increase). Expression of *CDKN2B*, as a well-known methylation regulated tumor suppressor gene, was validated by RT-PCR before [36]. Furthermore, expression of five selected genes, *GCG*, *NMES-1*, *LRMP*, *FAM161B*, and *PTGDR*, was also tested on independent laser microdissected colonic and also on demethylated HT-29 cells by real-time PCR. The applied PCR primer sequences are given in Table S4.

#### 5-Aza treated HT-29 cells

**Figure 3:**
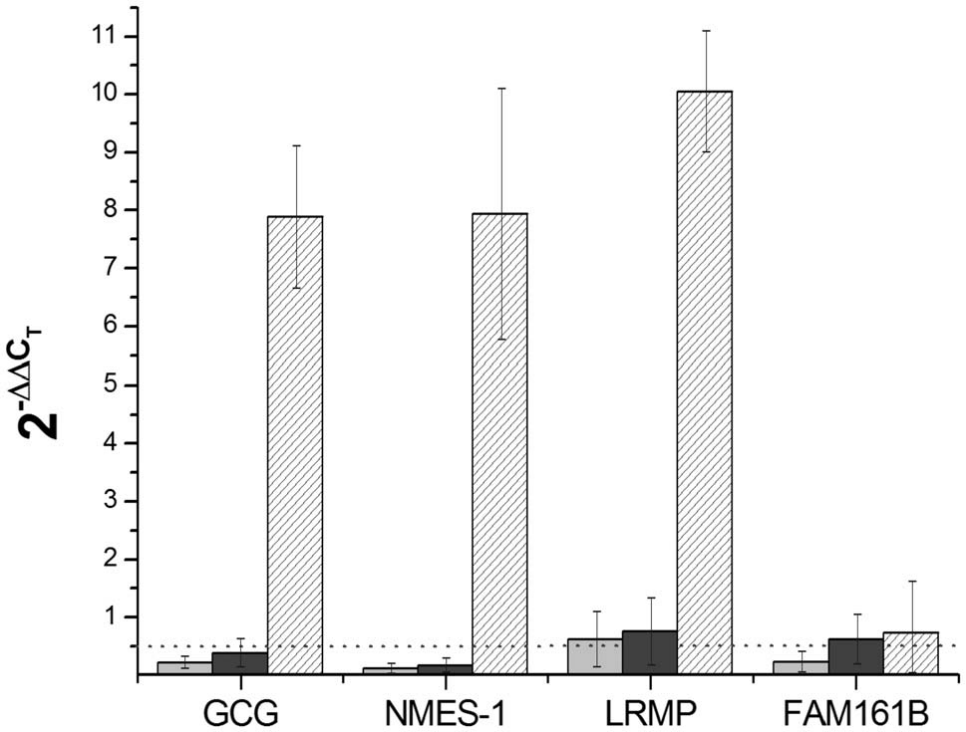
The expression of *GCG*, *NMES-1*, *LRMP*, and *FAM161B* genes, tested on independent laser microdissected colonic and also on demethylated HT-29 cells by real-time PCR. Data analysis was carried out with the comparative Cp method (see Materials and Methods). Genes were considered to be downregulated with 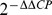
values lower than 0.5 (50% decrease, horizontal dotted line), and upregulated with values higher than 2 (two-fold increase). GAPDH was used as a control housekeeping gene. Light grey and dark grey columns show gene expression levels in adenoma and in tumor samples relative to normal samples, respectively. Hatched columns indicate the comparison of 5-Aza treated and control cells. Standard deviations of the measured transcript levels were calculated and are indicated for each transcript. Housekeeping gene intensities were averaged for each group.

To study the effect of demethylation, HT-29 cells were treated with 10 µM 5-Aza and the expression level of the selected gene group was examined in comparison with the acetate-treated (solvent for 5-Aza) control samples (Figure 3). After the demethylation, *GCG*, *NMES-1*, and *LRMP* genes showed increasing expression, while transcription of *FAM161B* gene did not change significantly. Similar results were obtained with a 20 µM 5-Aza treatment (data not shown).

#### LCM tissue samples

The RT-PCR results were compared in the normal colon mucosa-adenoma and normal colon mucosa-tumor relations in individual tissue samples. These five normal epithelial, five adenoma and four tumor samples were independent of the ones used in the microarray analysis. Results showed good agreement with the microarray analysis, *GCG*, *NMES-1*, and *FAM161B* genes were downregulated in both the adenoma and the tumor samples, while *LRMP* showed reduced expression only in the tumor samples (Figure 3).

**Figure 4:**
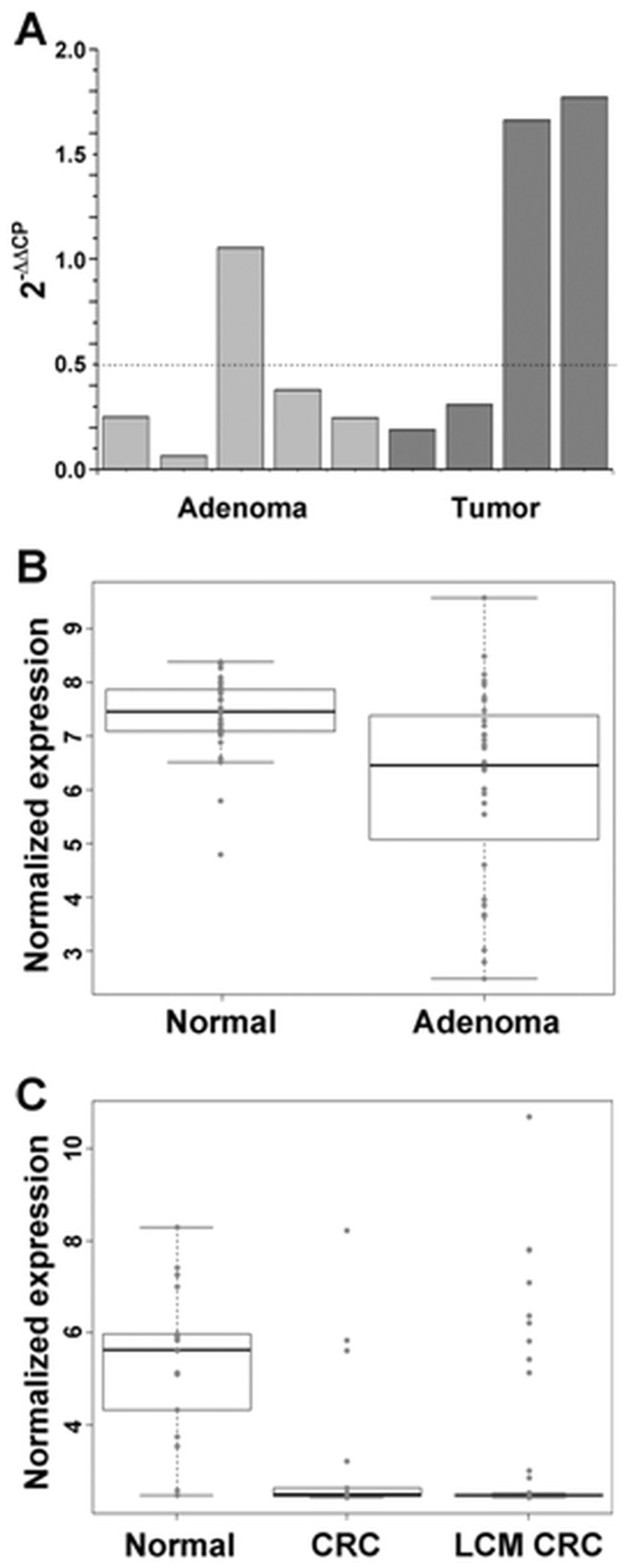
Heterogeneity of PTGDR mRNA expression levels in laser microdissected adenoma and tumor samples. Real-time PCR and data analysis was carried out as described in Materials and Methods. Genes were considered to be downregulated with 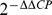
values lower than 0.5 (50% decrease, horizontal dotted line) (A) Expression value distribution of the 215894_at probeset (for *PTGDR* gene) in the GSE8671 (B) and GSE18105 (C) GEO data sets. P values: Normal vs. Adenoma P = 0.000712; CRC vs. Normal P = 0.0003797; LCM CRC vs. Normal P = 0.0000004; LCM CRC vs. CRC P = 0.834.

*PTGDR* expression showed ambiguous results in the RT-PCR experiments. It did not show a strong response for the 10 µM 5-Aza treatment, but had about 1.7-fold increased expression when cells were treated with 20 µM 5-Aza. Also, PTGDR RNA levels showed heterogeneity in the tissue samples (Figure 4A), therefore we performed further analyses to reveal the background of these observations. Expression values for the 215894_at PTGDR probe set were tested on independent GEO sets (Figure 4 B, C). In these independent sample sets, decreased PTGDR mRNA levels were found already in the adenoma samples, which further decreased in CRC samples.

### Bisulfite-specific PCR and HRM (High Resolution Melting) Analysis

**Figure 5:**
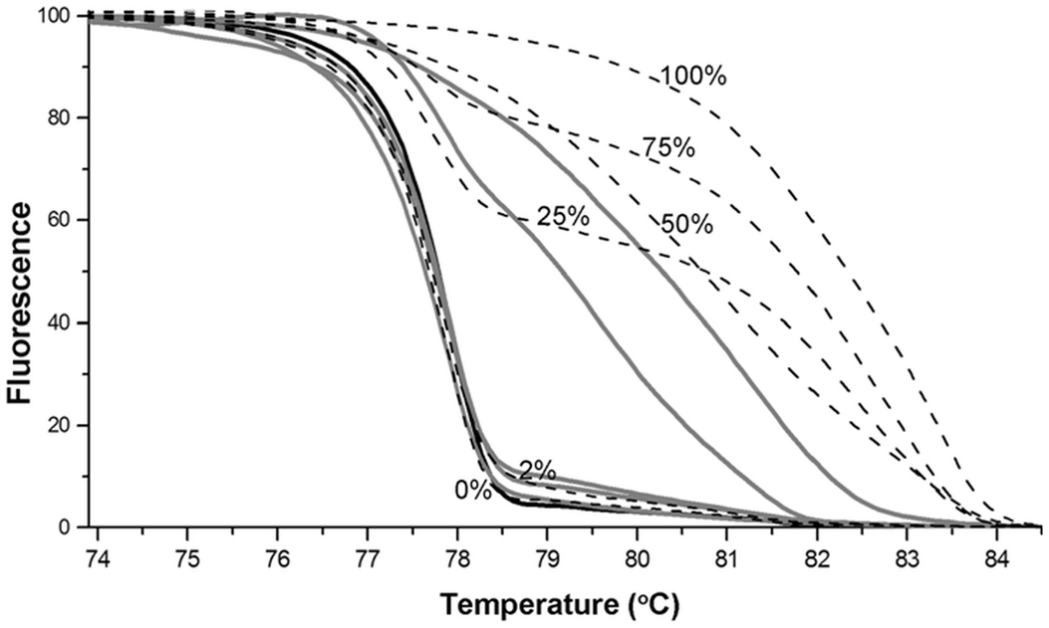
The normalized melting curves of the HRM analyses. Dashed lines represent the standard dilution series from artificially methylated DNA (0%, 25%, 50%, 75% and 100%). The solid black line represents results obtained from a normal sample. Solid grey lines represent results obtained from 5 independent tumorous tissue samples. The HRM analysis can distinguish methylation of the normal and tumorous tissues based on the different G:C contents in the bisulfite-converted promoter sequence. Promoter regions containing more methylated cytosines, which cannot be converted to uracil by the bisulfite treatment, has higher melting temperatures. The melting temperature is proportional to the ratio of non-converted cytosines.

To verify that the regulatory region of *PTGDR* is indeed hypermethylated, we performed bisulfite-specific PCR and high resolution melting analysis on genomic DNA [37]. The standard dilution series were used to test the sensitivity of our HRM assays. According to the normalized melting curves, our assay was able to detect 2% methylated DNA in 98% unmethylated background. The methylation percent of tissue samples was estimated according to their normalized melting curves compared to the standard dilution series. All normal colon mucosa samples normalized melting curve fell between the areas delimited by the 0%-2% standard samples. In case of the tumor samples, 1 specimen was in the 0%-2% methylation range, 2 between 2–25% and two samples showed methylation ratio higher than 25% (Figure 5). Analysis of the selected *PTGDR* region from HT29 cell line showed nearly 100% methylation. For validation, bisulfite sequencing was performed to determine the methylation status of this region. (Figure S2.)

### Tissue Microarray Analysis, PTGDR Immunohistochemistry

**Figure 6:**
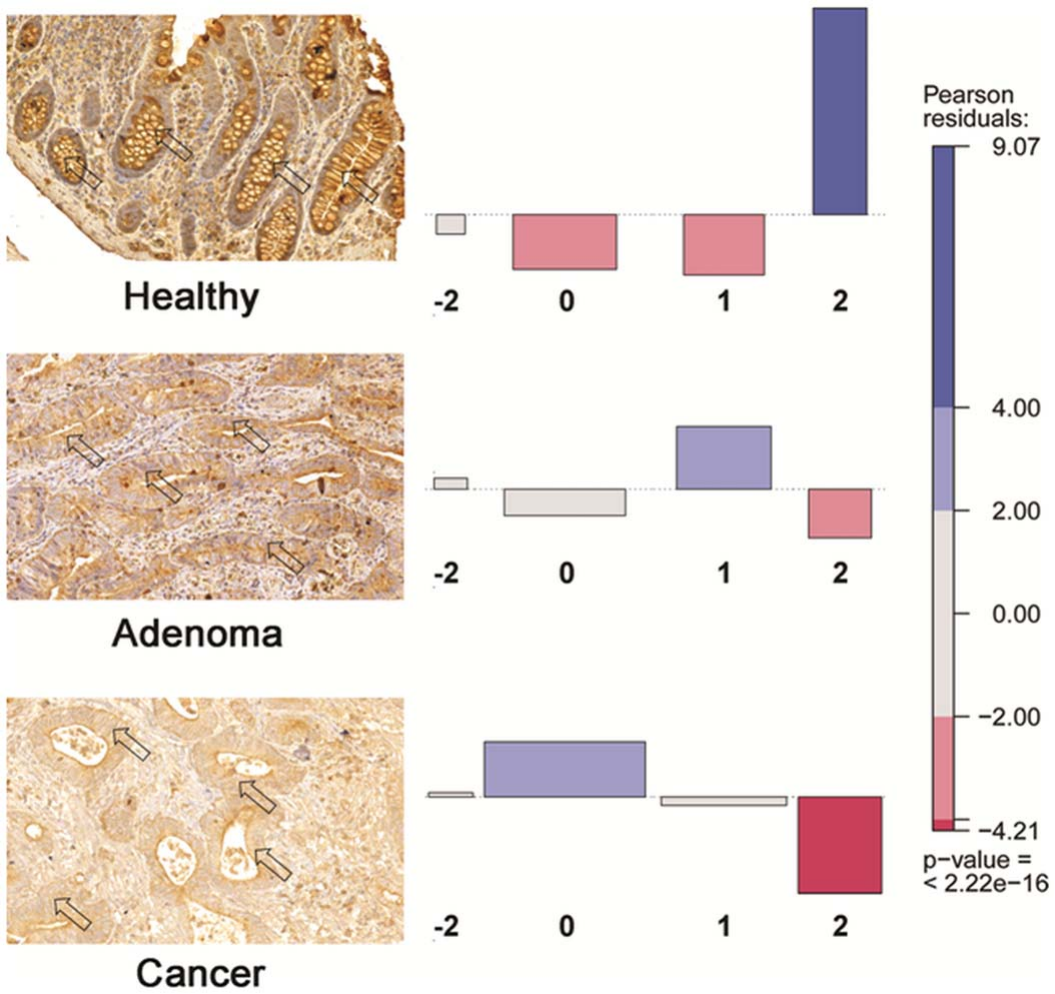
Immunhistochemical staining (20× magnification) of the prostaglandin D2 receptor (PTGDR) protein (left panel) on normal colon (top), adenoma (middle) and tumorous (bottom) colonic tissues illustrating the progressive decrease of the intraepithelial PTGDR protein expression (arrows) in the adenoma-carcinoma sequence. In the normal samples (top), predominantly strong, diffuse cytoplasmic staining was detected, which moderately decreased in the adenoma samples (middle), and only weak cytoplasmic staining was observed in the colorectal cancer samples (bottom). The right panel shows the association plots which represent tendentiously decreasing PTGDR protein expression along the adenoma carcinoma sequence. To measure the association of two variables (expression and disease stage), the Chi-square test was used. The height (and color depth) of the boxes is proportional to the difference between the observed and expected frequency of scores. The downward red colored boxes indicate that the observed frequency is lower than expected. The upward, blue items represent the opposite. PTGDR immunostaining scores: *−*2 = no staining; 0 = weak staining; 1 = moderate staining; 2 = strong diffuse epithelial cytoplasmic immunostaining.

The prostaglandin D2 receptor was further studied by immunohistochemistry using TMA slides to determine the effect of DNA methylation on the protein level. Normal colon mucosa samples showed strong epithelial cytoplasmic immunostaining, which sequentially decreased in the adenoma stage near the luminal surface. The immunostaining intensity was further reduced during the progression and only moderate expression could be observed in the tumor samples (Figure 6 left panel). Although in some adenoma and tumorous samples the protein expression was found to be similar to what was detected in the normal epithelium (figure S3). A tendentious PTGDR protein expression decrease was observed in the adenoma-carcinoma sequence progression (Figure 6 right panel).

### Testing of Methylation Regulated Genes on Independent Sample/expression Sets by PCA

**Figure 7:**
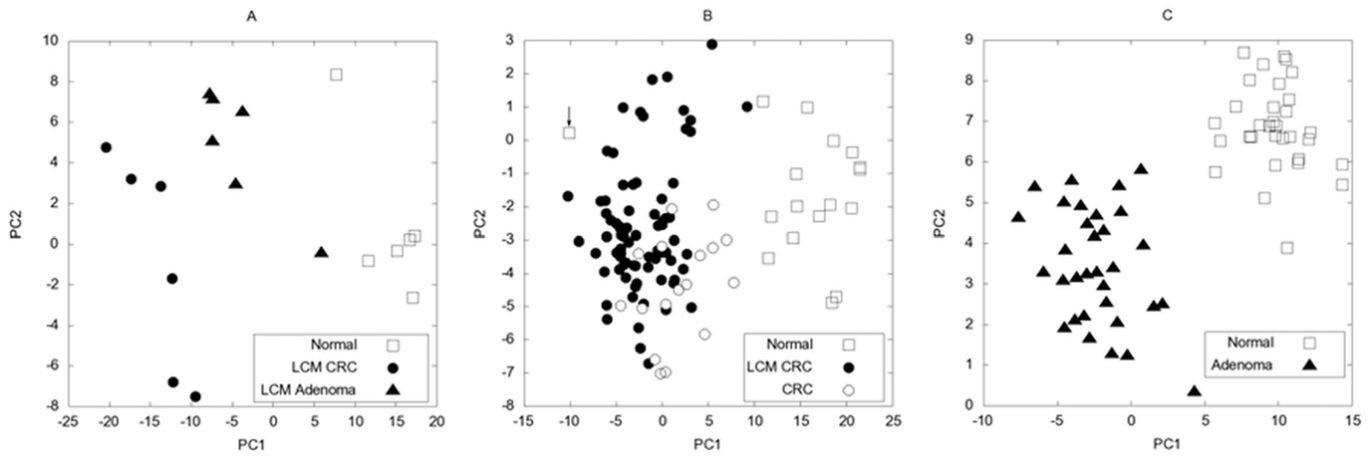
The first 2 principal components (PC) for our LCM (GSE15960) (A), the GSE18105 (B) and GSE8671 sample sets (C). The principal components were calculated from the log_2_-expression values of the 154 selected probe sets for the normal-CRC samples in our LCM set, and then all 3 sets were projected into the principal component coordinate space. Note that this method can be considered as an unsupervised classification, since we did not use explicitly the categories in the data analysis process. Figure 7.A shows that PC transformation separates very well the normal and LCM CRC samples and places the adenoma samples between normal and CRC samples. To validate our list of potential marker genes, we transformed the data of two other independent studies into the same PC coordinates. The two studies from the Gene Expression Omnibus microarray archive were GSE18105 with normal, CRC and LCM CRC samples and GSE8671 with normal and adenoma samples. For both sets the separation of the categories is good except for one outlier point in B marked with an arrow (see discussion in text).

To test the discriminatory power of the 154 selected genes, which are likely regulated by methylation during carcinogenesis, we have tested them on two independent sample sets from the Gene Expression Omnibus (GEO) public microarray archive. One of the sample sets, GSE8671 [38], contains normal and adenoma, and the other, GSE18105 [39], normal and tumor samples. The latter sample set is especially suitable for validation, since it contains both homogenized and LCM tumor samples. Activities of all the 154 genes in our list were compared using a standard dimension reduction method, principal component analysis (PCA) [40] which transforms the multivariate log_2_-expression data into 2 dimensions. PCA selects the directions with the largest variance then rotates and projects the data into this space. We calculated the transformation matrix using our 6 normal and 6 LCM CRC samples (the adenoma samples were not used during the calculation of the transformation matrix) and used the resulted transformation matrix to project all the samples into the space spanned by the first 2 principal components. The first two components contain 81% of the cumulative total variance; hence one can expect them to faithfully represent the multivariate distribution. Figure 7A shows that the normal and LCM CRC samples are clearly separated, and when the adenoma samples are projected into the PCA space, they are located in between the normal and CRC samples, supporting the hypothesis that adenoma is a transitional state between the normal and CRC phases [2], [41]. Similar results were obtained using only the six genes listed in Figure 2 (data not shown). The discriminatory power of the selected 154 genes was further validated using previously published data sets. The PCA projection which was calculated using solely our LCM CRC and normal samples could also completely separate the normal and adenoma subsets of the GSE8671 study (see Figure 7C). For the other GEO dataset, GSE18105, the separation is almost perfect for both the homogenized CRC and LCM CRC samples, only one point, marked with an arrow in Figure 7B is misclassified. To find the reason of misclassification Euclidean distance analysis was performed in which this particular sample was proven to be an outlier (Supplementary Figure S4).

## Discussion

In this study we present a novel high-throughput screening for the selection of a gene group, whose altered gene expression due to the aberrant DNA methylation pattern can be related to cancer. Activity of such genes may inhibit cancer progression, therefore their identification could improve the determination of prognosis.

In a previous work, we have developed an experimental system for identification of methylation regulated genes based on the examination of gene expression levels in clinical LCM samples and in 5-aza treated HT-29 cells. 110 transcripts showed decreased expression in tumor development and 71 upregulated transcripts were identified and a result of the 5-aza treatment. 17 transcripts belonged to both groups, and these transcripts were assumed to be regulated by DNA methylation [28].

In the present work a much broader analysis strategy was used, resulting in identification of 2533 downregulated transcripts during tumor development, and 3000 upregulated transcripts as a result of 5-aza treatment of HT-29 cells (Figure 1). Methylation related 154 transcripts were present in both groups, i.e. had decreased expression in tumors compared to normal colon mucosa cells and showed increased activity after demethylation treatment of HT-29 colon cancer cells. Therefore these transcripts are likely inhibited by methylation, directly or indirectly during the progression of colorectal cancer. The validity of this list is supported by (i) the presence of many genes which were previously found to be downregulated in colorectal cancer (e.g. *CHGA*, *FCGBP*, *GSN*, *LPP*, *MYH11*, *PLCG2*, *SST*, *NBL1*) [42], [43], (ii) the presence of several known tumor suppressor genes (e.g. *CDKN2B*, *MTUS1*, *RASSF6*, *PDCD4*, *KLF5*, *CDS1*), and (iii) by the results of principal component analyses performed on previously published data sets. There are various genetic and epigenetic factors that can contribute to the decreased expression of genes in tumor cells. We were interested in genes which can be silenced by DNA methylation. Previous studies showed that many genes are inactivated in colorectal cancer cell lines, however, there are large variations between the different cell lines [16], [17], suggesting that not all the inactivated genes are related to tumorigenesis. According to our hypothesis, activity of several genes identified by our approach may interfere with tumor progression. This hypothesis is supported by the presence of the cyclin-dependent kinase inhibitor 2B (*CDKN2B*) and 2C (*CDKN2C*) tumor suppressor genes in the identified gene set. The *CDKN2B* gene is located on chromosome 9p21, a locus at which deletions frequently occur in many primary human tumors, including esophageal carcinoma [44] and colorectal cancer [45]. *CDKN2B*, which was downregulated in our adenoma and colorectal tumor samples is also silenced by DNA methylation in a variety of haematological malignancies [46]. *CDKN2C* was previously reported to be inactivated by promoter hypermethylation in Reed-Sternberg cells in Hodgkin lymphomas, and the loss of CDKN2C showed negative correlation with the overall survival of the patients [47]. Retinoic acid receptor responder 1 (*RARRES1*) is also a tumor suppressor gene. Its expression is frequently downregulated through DNA hypermethylation in several types of malignant tissues. Downregulation of *RARRES1* was suggested to be related to stage D progression of colorectal cancer [48]. Underexpression of several other genes, identified by our approach, was previously described to be associated to colon cancer. For example, the methyl-CpG binding domain protein, MBD4, which is involved in mismatch repair at CpG sites, is affected by frameshift mutations in over 40% of microsatellite unstable sporadic colon cancers [49]. The human polimeric immunoglobulin receptor (PIGR) was found to be underexpressed in colon tumors and also in colon tumor cell lines [50]. Similar to our results, the Ephrin-A5 gene (*EFNA5*) was also reported before as a downregulated gene in colon cancer [51]. The expression of the POU domain class 2 transcription factor 3 (POU2F3) was decreased in the adenoma and the tumorous colonic epithelia. CpG islands in the *POU2F3* regulatory region are often aberrantly methylated in cervical cancer [52].

To evaluate the accuracy of our approach, we measured the expression of five genes by RT-PCR in independent tissue samples. These genes were also identified in our previous pilot study but they have not been linked to CRC before. In case of two genes, *GCG* and *NMES-1*, a remarkable decrease was found in the gene expression already in the adenoma stage. Both genes showed a strong reactivation as a result of a demethylation treatment of HT-29 cells, suggesting that these genes are inactivated by promoter hypermethylation. The *NMES-1* gene (also named as *C15orf48*) is expressed along the healthy gastrointestinal tract and it is frequently downregulated in esophageal squamous cell carcinomas [53]. Aberrant methylation of the *NMES-1* promoter region was also detected in invasive cervical cancer (ICC), but not in normal cervical samples [54]. The protein encoded by the glucagon (GCG) gene is a preproprotein which is cleaved into four distinct mature peptides. These peptides are involved in maintaining nutrient homeostasis, and are regulators of cell proliferation, differentiation, and apoptosis [55]. *FAM161B* is a predicted gene, whose contribution to carcinogenesis has not been reported yet. It was underexpressed in both stages in our experiments, however, it did not show a remarkable activation as a result of 5-Aza treatment.

The prostaglandin D2 receptor (*PTGDR*) gene is located in the prostaglandin receptor cluster. In previous studies the methylation status of *PTGDR* was found to be correlated inversely with its expression in neuroblastoma cell lines [56]. Our microarray results suggested that the PTGDR mRNA level decreases along the adenoma-carcinoma sequence on average. However, analysis of the PTGDR mRNA level by RT-PCR and the PTGDR protein level by immunohistochemistry in individual tissue samples showed heterogeneity. Similar results were obtained by analyzing the previously published GSE8671 and GSE18105 gene expression data sets (Figure 4). The HRM assays detected different levels of CpG methylation in the PTGDR promoter region in individual colon cancer samples. These observations suggest that the *PTGDR* gene is silenced by DNA hypermethylation during the development of certain colorectal tumors. However, it needs further investigations whether PTGDR silencing shows a correlation with the prognosis of the disease.

The microarray data together with the validation results suggest that DNA methylation partially inactivates certain genes already in the early adenoma stage. This observation can be important in the future, because after the early detection of colorectal cancer, gene-specific therapies or targeted gene activation methods can have relevant importance. Interestingly, genes which are downregulated in the adenoma stage do not always show a gradually decreasing activity along the adenoma–carcinoma sequence, i.e. they are expressed at a lower level in the adenoma than in the tumor samples. Also, we observed higher uniformity of adenoma samples than colorectal cancer samples in the PCA analyses of gene expression data sets (Figure 7C vs 7B). This observation suggests that certain genes which are needed to be inactivated for adenoma formation are reactivated in the tumor. It is easier to obtain such patterns of gene activity by reversible epigenetic regulation than by mutations. Methylation-mediated regulation can affect about 60% of the human promoters [11], and allows fine-tuning of gene activities to obtain an optimal combination of expression. This optimal combination may depend on several factors and can change during tumor progression. However, because of the limited number of samples in this work, further studies are needed to verify this conclusion.

Downregulation of many genes identified by our approach is related to tumorigenesis. However, further studies are needed to answer whether the set of underexpresed genes presented in this study is specific to colorectal cancer. Applying our approach to other tumor models would allow investigation of the tumor-specificity of hypermethylation mediated gene inactivation patterns.

## Materials and Methods

### Sample Collection

Tissue samples obtained from surgically removed colon tissues were snap-frozen in liquid nitrogen and stored on −80°C until use. The study included adenoma with low-grade dysplasia samples and left side (sigmoid, rectum), Stage II, moderately differentiated colorectal tumors. The paired control normal colon mucosa specimen originated from the histologically normal area of the removed sample farthest available from the tumor.

The clinicopathological diagnosis was made for each sample by a pathologist. Ethics Committee approval was obtained (Nr.: TUKEB 2005/037. Semmelweis University Regional and Institutional Committee of Science and Research Ethics, Budapest, Hungary) and and written informed consent was provided by all patients [28].

### Laser Captured Microdissection (LCM)

Specimen were embedded in TissueTek OCT compound (Sakura Finetek, Japan), then series of 6 µm sections were mounted onto PALM Membrane Slide 1.0 PEN (Carl Zeiss, Bernried, Germany) at −20°C. The slides were stored at −80°C until ethanol fixation and staining was performed by cresyl violet acetate (Sigma-Aldrich, St. Louis, USA), an alcohol diluted stain [57]. A total of 5×10^3^ colonic epithelial cells were collected from each section in 5 biological replicates using the PALM Microbeam system (PALM, Bernried, Germany) [28].

### Cell Culture Model and 5-Aza Demethylation Treatment

HT-29 colon adenocarcinoma cells (ATCC Number: HTB-38) were grown in RPMI-1640 containing 10% FCS (Sigma-Aldrich) on 37°C in 5% CO_2_ concentration [49]. In 25 cm^2^ cell culture flask, 1,5×10^6^ cells/flask were cultured for 1 day, then for demethylation the cells were treated with 10 µM 5-aza-2′-deoxycitidine (Sigma-Aldrich) for 72 hours in FCS-free medium. In the control cultures PCR-grade water and acetate, the solvent of 5-Aza was added in 1∶1 ratio [28].

### RNA Isolation and Quality Control

Total RNA was extracted from the microdissected tissue samples and from the treated HT-29 cells using RNeasy Micro Kit (Qiagen) according to the manufacturer’s instruction. The quality of the isolated nucleic acid was measured by microcapillary electrophoresis system using RNA 6000 Pico LabChip kit (Agilent BioAnalyzer 2100). For the microarray analysis 5–50 ng total RNA was used from the samples with RIN (RNA Integrity Number) between 7–10.

### Microarray Analysis

Microarray experiments were performed according to the recommendation of Minimum Information About a Microarray Experiment (MIAME) guideline [58]. For the amplification and the labeling of the transcripts in case of the HT-29 cells single-round in vitro transcription was performed by using the One-Cycle Target Labeling and Control Kit (Affymetrix, Santa Clara, CA, USA), while the microdissected samples - due to their lower template amount- required two-cycle T7-based linear amplification by using the Two-Cycle Target Labeling and Control Kit (Affymetrix) [59], [60]. The samples were hybridized on HGU133 Plus2.0 arrays (Affymetrix) at 45°C for 16 hours, the microarrays were washed and stained with Fluidics Station 450 device (Affymetrix) by EukGE_Ws_2v5 wash protocol using an antibody-based signal amplification method according to the manufacturer’s instructions (with 10 µg/ml streptavidin-phycoerythtrin (Molecular Probes)). Fluorescent signals were detected with GeneChip Scanner 3000 (Affymetrix). The datasets of LCM and cell culture experiments are available in the Gene Expression Omnibus databank for further analysis (http://www.ncbi.nlm.nih.gov/geo/), series accession numbers: GSE15960 [61] and GSE29060 (this study).

### Independent Gene Expression Omnibus Datasets

Microarray datasets with HGU133 Plus2.0 experiments obtained from colonic biopsy/tissue samples collected by other research groups were downloaded from the Gene Expression Omnibus (GEO) database (dataset IDs: GSE8671 [38], GSE18105 [39]). Our selected gene panels were tested on the downloaded datasets, and discriminatory efficacy was determined using principal component analysis (PCA) and hierarchical cluster analysis.

### Statistical Evaluation

From the CEL files, quality control and RNA digestion plot were generated in R-environment using the Bioconductor system. After data preprocessing, the differentially expressed genes between the analyzed sample groups were determined by SAM (Significance Analysis of Microarrays) at the significance level of p<0.01. Feature selections were done according to the log_2_FC (log_2_ fold change) values to select at least two-fold up/downregulated genes. In case of the tissue specimen, the downregulated genes were identified with a log_2_FC value lower than −1. As the 5-Aza treatment of the HT-29 cells results in only partial demethylation, genes belonging to the top 3000 probe sets with the highest log_2_FC values at the significance level of p<0.025 were considered to be upregulated.

### Tissue Microarray Analysis (TMA), PTGDR Immunohistochemistry

Cores of 1 mm diameter were collected from selected areas of formalin-fixed, paraffin-embedded tissue blocks prepared from 37 low-grade and 44 high-grade dysplastic colorectal adenomas, 89 early stage CRC (Stage II), 57 advanced stage CRC (Stage III and IV) and 62 normal colonic samples of 119 patients and inserted into 4 recipient blocks taking 70 samples each. Five µm thick tissue sections were cut from the blocks. TMA analysis with PTGDR-specific immunohistochemistry was performed as described before [30]. Immunostained TMA slides were digitalized using high-resolution MIRAX DESK instrument (Zeiss, Gottingen, Germany), and analyzed with the MIRAX TMA Module software [62]. Protein expression was evaluated using an empirical scale considering intensity and proportion of positive cytoplasmic staining of epithelial/carcinoma cells. Scores were given for PTGDR: −2 for no staining; 0 for weak, 1 for moderate, 2 for strong diffuse immunostaining. Pearson’s Chi-test and Fisher’s exact test were done for revealing if the staining difference in progression groups was significant (p<0.05). Contingency tables and association plots were also constructed from the two categorical variables (group and score) [63].

### MTT Cell Proliferation Assay

In 96-well plates, 5×10^3^ HT-29 cells per well were maintained for 24 hours in 100 µl RPMI-1640 medium containing 10% FCS, after which, the cells were exposed with 5, 10, 20 and 100 µM 5-aza-2′deoxycitidine (Sigma-Aldrich, diluted in the 1∶1 mixture of PCR-grade water and acetic acid) for 48 or 72 hours in FCS-free medium. A volume of 0.5 mg/ml of MTT (methylthiazolyldiphenyl-tetrazolium bromide, Sigma-Aldrich) was then added to each well, and the cells were incubated for 4 hours at 37°C. The medium was carefully removed, and blue formazan – spawned from MTT by the mitochondrial dehydrogenase enzyme system of cells – was diluted in DMSO. Absorbance was measured at 570 nm using a Multiscan MS ELISA plate reader (Thermo Fisher Scientific Inc., Waltham, MA, USA).

### Real-time PCR Validation

Real-time PCR was used to assess the expression of selected genes in 5 adenoma and 5 tumor-normal paired laser microdissected colonic samples and also in HT-29 cell culture treated with 10 µM 5-Aza. The preparation protocols of the samples were similar to those which were previously applied for the microarray analyses. The reverse transcription was performed in a final volume of 15 µl using MultiScribe Reverse Transcriptase enzyme (50 U/µl), RNase Inhibitor (20 U/µl), 10× RT buffer and 100 µM dNTP from the TaqMan Reverse Transcription Kit (Applied Biosystems, Carlsbad, USA) to synthesize cDNA from 1 µg of total RNA per each templates, where the primers (200 nM) were specific to the selected transcripts (Table S1). Due to the limited amount of the laser microdissected samples, the cDNA was necessary to be amplified before the PCR reaction, thus a pre-amplification with 12 cycles was performed. In 50 µl final volume 5 µl template, 25 µl LightCycler Probes Master (2x) (Roche), 3 µl gene-specific primer and 17 µl PCR-grade water were mixed and incubated as follows: 94°C 5 minutes, for 12 cycles: 94°C for 15 seconds, 60°C for 15 seconds, 72°C for 15 seconds, and 2 minutes final extension on 72°C. The real-time PCR reactions were performed in 10 µl final volume with LightCycler Probes Master (2x) (Roche) according to the manufacturer’s instructions. The PCR reactions were automatically compiled in 384-well plates by Eppendorf epMotion 5070 and all assays were carried out in triplicates. The pre-amplified samples were analyzed in SYBR Green assays on LightCycler 480 system (Roche) using the following thermocycling conditions: 95°C for 5 minutes, 95°C for 10 seconds, 60°C for 10 seconds, 72°C for 10 seconds repeated in 45 cycles, 65°C after 1 minute continuous warming to 97°C, and finally 40°C for 30 seconds. Data analysis was carried out with the comparative crossing point (Cp) method [64] after determination of the related Cp values based on the 2nd derivative maximum method [65]. The GAPDH housekeeping gene was used as a reference for all the LCM and cell culture samples.

### Bisulfite-specific PCR and HRM (High Resolution Melting) Analysis

Prostaglandin D2 receptor (*PTGDR*) gene has been selected for further methylation analyses. Genomic DNA was extracted from fresh frozen tissue (5 normal colon, 5 tumor samples) and HT-29 cells using High Pure PCR Template Preparation Kit (Roche). For calibration of the analysis, 0%, 2%, 25%, 50%, 75% and 100% artificially methylated DNA samples were prepared by mixing methylated (Universal Methylated Human DNA Standard, Zymo Research) and unmethylated (Unmethlyated EpiTect Control DNA, Qiagen) samples in the appropriate ratios. From each template 300 ng DNA was bisulfite converted by methylSEQr kit (Applied Biosystems) according to the manufacturer’s instructions. The CpG islands in the gene’s promoter region were predicted by CpG Plot EMBOSS Application http://www.ebi.ac.uk/Tools/emboss/cpgplot/index.html [66] and primers were designed using MethPrimer software http://www.urogene.org/methprimer/index1.html [67] to amplify a region of the identified CpG islands. The specificity of the primers were tested *in silico* by the BiSearch software http://bisearch.enzim.hu [68] (Table S4). Bisulfite-specific PCR reactions were perfomed in a final volume of 15 µl using AmpliTaq Gold 360 PCR Master Mix (2x) (Applied Biosystems), ResoLight HRM Dye (20x) (Roche), bisulfite-specific primers (200 nM) and 5 ng/well bisulfite converted DNA template. The amplification was carried out with the following thermocycling conditions: 95°C for 10 minutes, 95°C for 30 seconds, 58°C for 30 seconds, 72°C for 30 seconds for 10 touchdown cycles, 95°C for 30 seconds, 53°C for 30 seconds and 72°C for 30 seconds in 40 cycles. On completion of the PCR thermal cycling, for the HRM analysis the samples were denatured at 95°C for 1 minute, cooled down to 40°C and held for 1 minute, then continuously warmed up to 95°C with 0.03°C/second rate during the melting curve fluorescence acquisition. The Cp values and the normalized melting curves were retrieved after data preprocessing using the LightCycler 480 Software release 1.5.0 (Roche).

## Supporting Information

Figure S1 **Heatmap of 108 potentially methylation regulated transcripts at the early stage of carcinogenesis, based on the adenoma-carcinoma sequence progression model.** This group of transcripts showed downregulation in tumors (T) compared to normal cells (N), and overexpression after 5-Aza treatment. Furthermore, these genes were found to be under expressed already in the normal-adenoma transition (Ad).
(TIF)

Figure S2 **(A) Results of bisulphite sequencing on the** ***PTGDR*** **gene promoter region in case of normal and tumorous biopsy samples, and HT-29 cell line.** This region contains 24 CpG dinucleotides, which are potential targets of methyl-transferases. In normal samples only converted cytosines were detected with higher T peak (red) (see the chromatogram of a representative normal sample). In tumorous samples higher C peaks (blue) were observed in position 2, 8, 9, 14, 15 and 23 which originate from the non-convertible, methyl group containing cytosines. In the HT-29 cell line this region was found to be completely methylated (non-convertible). (B) Methylation status heatmap of the examined CpG positions by bisulphite sequencing data. Black and white rectangles are indicate the totally methylated (100%) and unmethylated (0%) CpG positions, respectively.
(TIF)

Figure S3 **PTGDR immunohistochemistry on tumor samples.** Although low PTGDR protein level was observed in most tumor epithelial samples (see Figure 5), in some well differentiated early stage CRC cases strong dark brown staining (representing high PTGDR protein expression) could be detected (indicated by red arrows).
(TIF)

Figure S4 **Euclidean distance analysis performed on the GSE18105 dataset.** The aim of this analysis was to clarify the reason for the misclassification of sample GSM452639 in the PCAs. There were no alterations in microarray QC parameters including histogram of fluorescence intensity, RNA degradation and proportion of GAPDH and ACTB transcripts 3′/5′ intensity ratios. However, the Euclidean distance calculation using 17 normal and 17 homogenized CRC tissue samples from the GSE18105 dataset resulted in clear separation of normal and tumorous samples. One of the normal samples (GSM452639, indicated by an arrow) which was also found to be an outlier in PCA, generates a distinct cluster in the distance analysis. This indicates that the misclassification of this sample could be resulted from an error in the sample collection or sample handling process.
(TIF)

Table S1 **Downregulated transcripts in tumor compared to normal epithelial cells.** In tumorous epithelia, 2533 downregulated transcripts were identified which belong to 1509 known genes.
(PDF)

Table S2 **Putative methylation regulated transcripts in LCM CRC samples.** 154 common elements of 2533 downregulated transcripts in LCM tumor samples and 3000 upregulated transcripts from 5-Aza-treated HT29 cells.
(PDF)

Table S3 **Putative methylation regulated transcripts downregulated in both adenoma and CRC samples.** From the 154 methylation related transcripts (listed in Supplementary Table S2.), 108 were also downregulated in adenoma samples. These transcripts were found to be inactivated by DNA methylation at the early phase of carcinogenesis. Supplemetary Figure S1 shows the expression pattern of these genes in normal, adenoma and tumor samples.
(PDF)

Table S4 **Applied primer sequences.** The table shows the primer sequences used for RT-PCR validation of mRNA levels of six genes (top) and for bisulphite sequencing on PTGDR promoter region sequence (chr.14∶52734410-52734668) (bottom).
(PDF)
