# Supplementary figures and images for: Genome-Wide Screening of Genes Regulated by DNA Methylation in Colon Cancer Development
Source: PLoS One. 2012 Oct 1;7(10):e46215. doi: 10.1371/journal.pone.0046215 (PMC3462205; doi:10.1371/journal.pone.0046215)

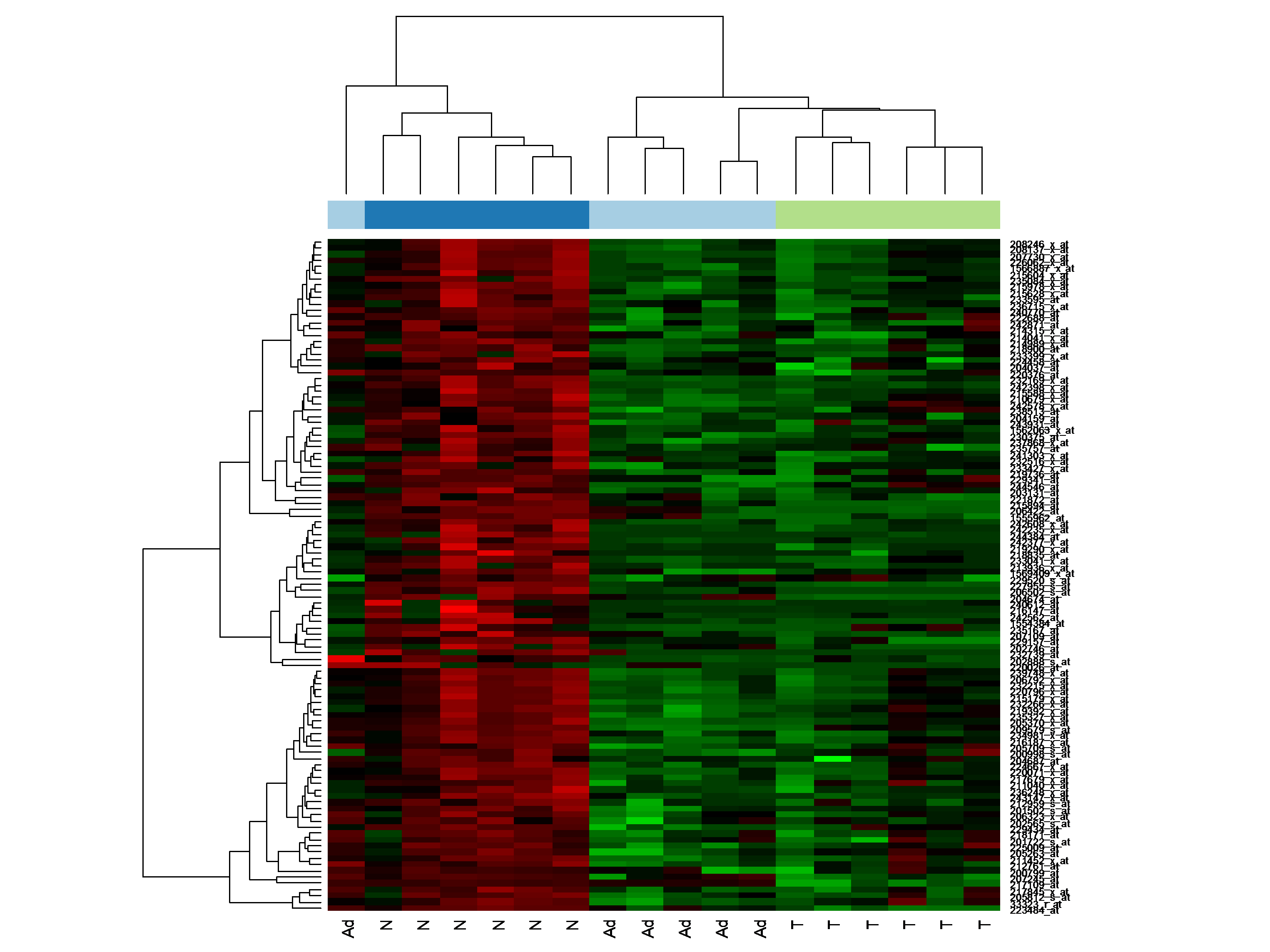

Supplement: Figure S1 — Heatmap of 108 potentially methylation regulated transcripts at the early stage of carcinogenesis, based on the adenoma-carcinoma sequence progression model. This group of transcripts showed downregulation in tumors (T) compared to normal cells (N), and overexpression after 5-Aza treatment. Furthermore, these genes were found to be under expressed already in the normal-adenoma transition (Ad). (TIF) [file pone.0046215.s001.tif]

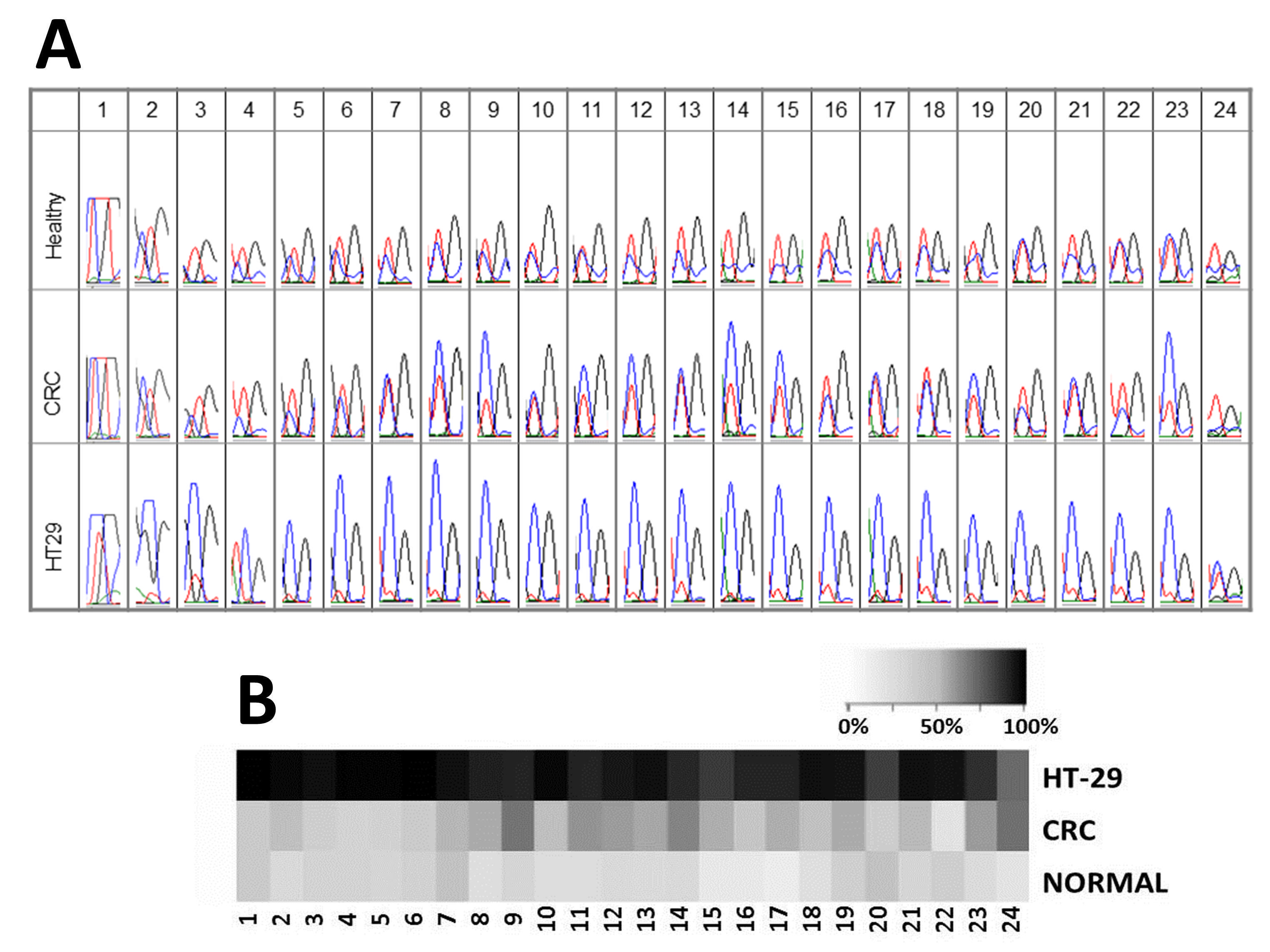

Supplement: Figure S2 — (A) Results of bisulphite sequencing on the PTGDR gene promoter region in case of normal and tumorous biopsy samples, and HT-29 cell line. This region contains 24 CpG dinucleotides, which are potential targets of methyl-transferases. In normal samples only converted cytosines were detected with higher T peak (red) (see the chromatogram of a representative normal sample). In tumorous samples higher C peaks (blue) were observed in position 2, 8, 9, 14, 15 and 23 which originate from the non-convertible, methyl group containing cytosines. In the HT-29 cell line this region was found to be completely methylated (non-convertible). (B) Methylation status heatmap of the examined CpG positions by bisulphite sequencing data. Black and white rectangles are indicate the totally methylated (100%) and unmethylated (0%) CpG positions, respectively. (TIF) [file pone.0046215.s002.tif]

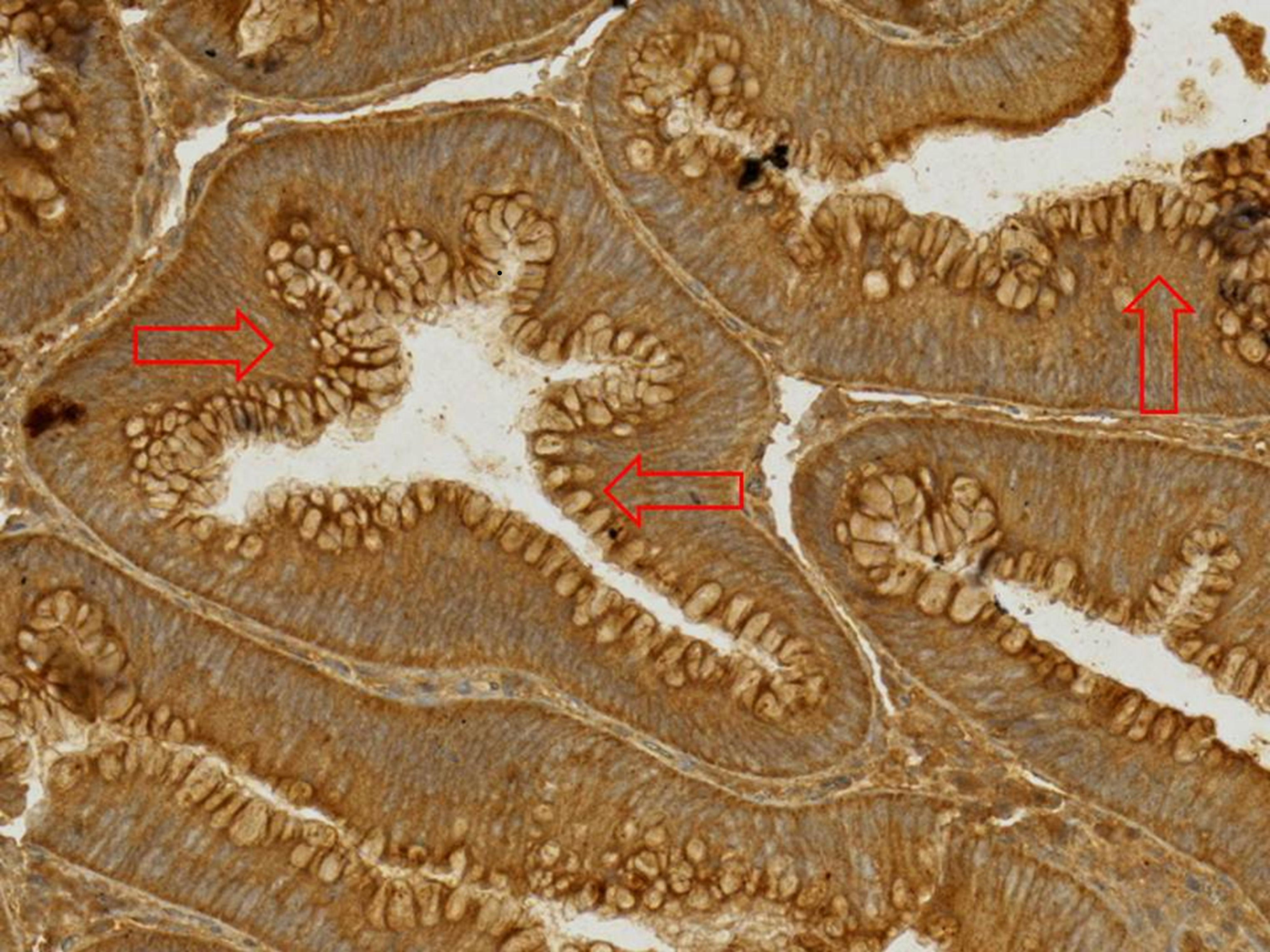

Supplement: Figure S3 — PTGDR immunohistochemistry on tumor samples. Although low PTGDR protein level was observed in most tumor epithelial samples (see Figure 5), in some well differentiated early stage CRC cases strong dark brown staining (representing high PTGDR protein expression) could be detected (indicated by red arrows). (TIF) [file pone.0046215.s003.tif]

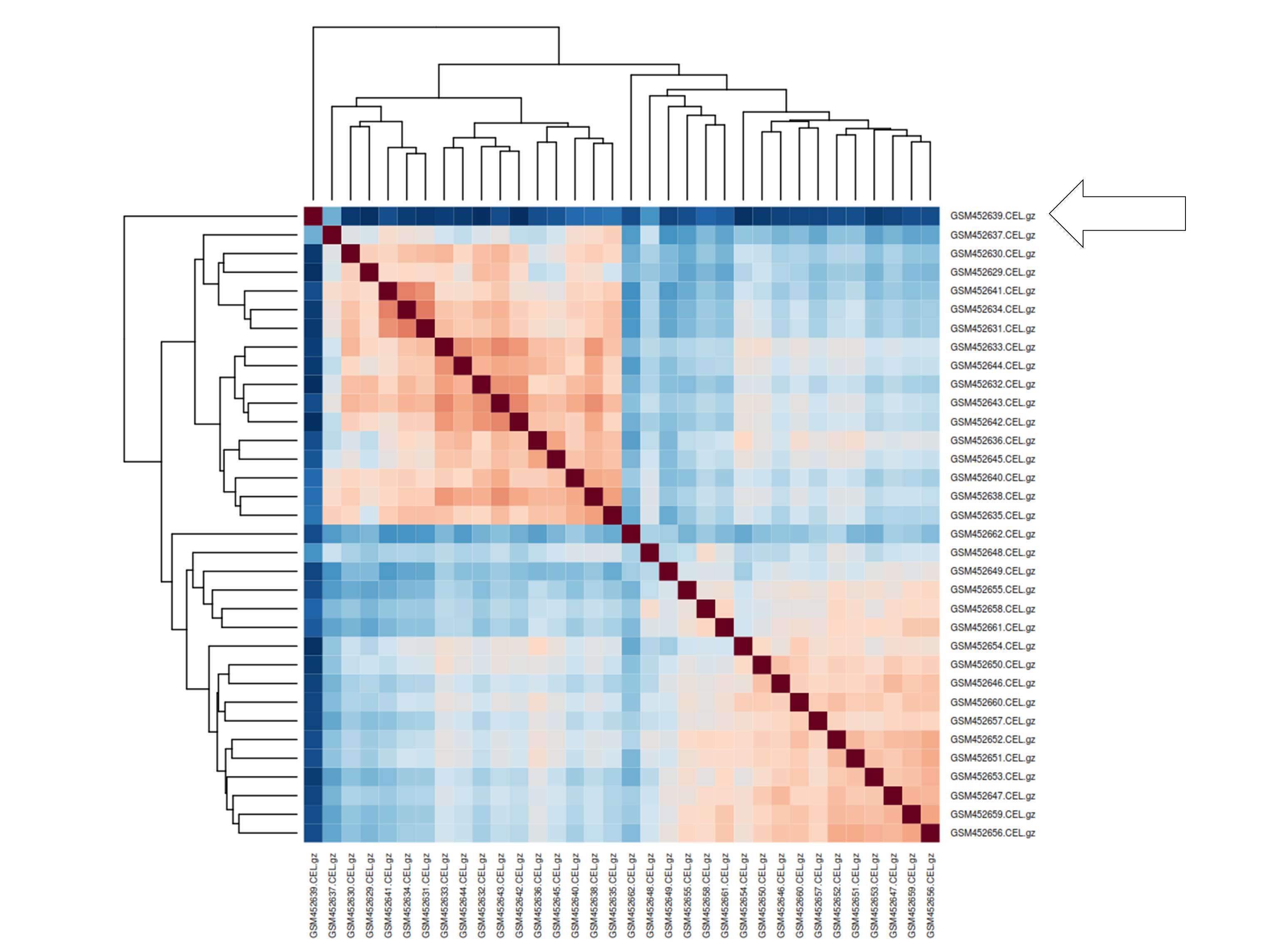

Supplement: Figure S4 — Euclidean distance analysis performed on the GSE18105 dataset. The aim of this analysis was to clarify the reason for the misclassification of sample GSM452639 in the PCAs. There were no alterations in microarray QC parameters including histogram of fluorescence intensity, RNA degradation and proportion of GAPDH and ACTB transcripts 3′/5′ intensity ratios. However, the Euclidean distance calculation using 17 normal and 17 homogenized CRC tissue samples from the GSE18105 dataset resulted in clear separation of normal and tumorous samples. One of the normal samples (GSM452639, indicated by an arrow) which was also found to be an outlier in PCA, generates a distinct cluster in the distance analysis. This indicates that the misclassification of this sample could be resulted from an error in the sample collection or sample handling process. (TIF) [file pone.0046215.s004.tif]
