# Supplementary material for: Genome-Wide Screening of Genes Regulated by DNA Methylation in Colon Cancer Development
Source: PLoS One. 2012 Oct 1;7(10):e46215. doi: 10.1371/journal.pone.0046215 (PMC3462205; doi:10.1371/journal.pone.0046215)
Supplement: Table S1 — Downregulated transcripts in tumor compared to normal epithelial cells. In tumorous epithelia, 2533 downregulated transcripts were identified which belong to 1509 known genes. (PDF) [file pone.0046215.s005.pdf]

Supplementary Table 1

Downregulated transcripts in tumor compared to normal epithelial cells

Probe Set ID	Gene Symbol	Gene Title
**213353_at**	ABCA5	ATP-binding cassette, sub-family A (ABC1), member 5
**242553_at**	ABCC3	ATP-binding cassette, sub-family C (CFTR/MRP), member 3
**202850_at**	ABCD3	ATP-binding cassette, sub-family D (ALD), member 3
**221927_s_at**	ABHD11	abhydrolase domain containing 11
**213017_at**	ABHD3	abhydrolase domain containing 3
**216929_x_at**	ABO	ABO blood group (transferase A, alpha 1-3-N-acetylgalactosaminyltransferase; transferase B, alpha 1-3-galactosyltransferase)
**203559_s_at**	ABP1	amiloride binding protein 1 (amine oxidase (copper-containing))
**214274_s_at**	ACAA1	acetyl-CoA acyltransferase 1
**49452_at**	ACACB	acetyl-CoA carboxylase beta
**202366_at**	ACADS	acyl-CoA dehydrogenase, C-2 to C-3 short chain
**200710_at**	ACADVL	acyl-CoA dehydrogenase, very long chain
**222688_at**	ACER3	alkaline ceramidase 3
**214763_at**	ACOT11	acyl-CoA thioesterase 11
**228603_at**	ACTR3	ARP3 actin-related protein 3 homolog (yeast)
**1563182_at**	ACVR1C	activin A receptor, type IC
**226950_at**	ACVRL1	activin A receptor type II-like 1
**205260_s_at**	ACYP1	acylphosphatase 1, erythrocyte (common) type
**210377_at**	ACSM3	acyl-CoA synthetase medium-chain family member 3
**234312_s_at**	ACSS2	acyl-CoA synthetase short-chain family member 2
**206134_at**	ADAMDEC1	ADAM-like, decysin 1
**203741_s_at**	ADCY7	adenylate cyclase 7
**207820_at**	ADH1A	alcohol dehydrogenase 1A (class I), alpha polypeptide
**206262_at**	ADH1C	alcohol dehydrogenase 1C (class I), gamma polypeptide
**214261_s_at**	ADH6	alcohol dehydrogenase 6 (class V)
**209869_at**	ADRA2A	adrenergic, alpha-2A-, receptor
**201924_at**	AFF1	AF4/FMR2 family, member 1
**232865_at**	AFF4	AF4/FMR2 family, member 4
**1552287_s_at**	AFG3L1	AFG3 ATPase family gene 3-like 1 (S. cerevisiae)
**1557820_at**	AFG3L2	AFG3 ATPase family gene 3-like 2 (yeast)
**221971_x_at**	AGAP10 /// AGAP4 /// AGAP5 /// AGAP9	ArfGAP with GTPase domain, ankyrin repeat and PH domain 10 /// ArfGAP with GTPase domain, ankyrin repeat and PH domain 4 /// ArfGAP with GTPase domain, ankyrin repeat and PH domain 5 /// ArfGAP with GTPase domain, ankyrin repeat and PH domain 9
**221850_x_at**	AGAP11 /// AGAP4 /// AGAP6 /// AGAP7 /// AGAP8	ankyrin repeat and GTPase domain Arf GTPase activating protein 11 /// ArfGAP with GTPase domain, ankyrin repeat and PH domain 4 /// ArfGAP with GTPase domain, ankyrin repeat and PH domain 6 /// ArfGAP with GTPase domain, ankyrin repeat and PH domain 7 /// ArfGAP with GTPase domain, ankyrin repeat and PH domain 8
**203566_s_at**	AGL	amylo-alpha-1, 6-glucosidase, 4-alpha-glucanotransferase
**224480_s_at**	AGPAT9	1-acylglycerol-3-phosphate O-acyltransferase 9
**225108_at**	AGPS	alkylglycerone phosphate synthase
**228969_at**	AGR2	anterior gradient homolog 2 (Xenopus laevis)
**228241_at**	AGR3	anterior gradient homolog 3 (Xenopus laevis)
**205357_s_at**	AGTR1	angiotensin II receptor, type 1
**232488_at**	AGXT2L2	alanine-glyoxylate aminotransferase 2-like 2
**212814_at**	AHCYL2	adenosylhomocysteinase-like 2

**221569_at**	AHI1	Abelson helper integration site 1
**211986_at**	AHNAK	AHNAK nucleoprotein
**244084_at**	AIFM3	apoptosis-inducing factor, mitochondrion-associated, 3
**202587_s_at**	AK1	adenylate kinase 1
**212172_at**	AK2	adenylate kinase 2
**236007_at**	AKAP10	A kinase (PRKA) anchor protein 10
**222024_s_at**	AKAP13	A kinase (PRKA) anchor protein 13
**230846_at**	AKAP5	A kinase (PRKA) anchor protein 5
**205771_s_at**	AKAP7	A kinase (PRKA) anchor protein 7
**215483_at**	AKAP9	A kinase (PRKA) anchor protein (yotiao) 9
**206561_s_at**	AKR1B10	aldo-keto reductase family 1, member B10 (aldose reductase)
**204151_x_at**	AKR1C1	aldo-keto reductase family 1, member C1 (dihydrodiol dehydrogenase 1; 20-alpha (3-alpha)-hydroxysteroid dehydrogenase)
**209699_x_at**	AKR1C2	aldo-keto reductase family 1, member C2 (dihydrodiol dehydrogenase 2; bile acid binding protein; 3-alpha hydroxysteroid dehydrogenase, type III)
**214259_s_at**	AKR7A2	aldo-keto reductase family 7, member A2 (aflatoxin aldehyde reductase)
**216381_x_at**	AKR7A3	aldo-keto reductase family 7, member A3 (aflatoxin aldehyde reductase)
**212224_at**	ALDH1A1	aldehyde dehydrogenase 1 family, member A1
**205208_at**	ALDH1L1	aldehyde dehydrogenase 1 family, member L1
**221590_s_at**	ALDH6A1	Aldehyde dehydrogenase 6 family, member A1
**214707_x_at**	ALMS1	Alstrom syndrome 1
**204294_at**	AMT	aminomethyltransferase
**228023_x_at**	AMY2B /// RNPC3	amylase, alpha 2B (pancreatic) /// RNA-binding region (RNP1, RRM) containing 3
**224667_x_at**	ANAPC16	anaphase promoting complex subunit 16
**205141_at**	ANG	angiogenin, ribonuclease, RNase A family, 5
**229457_at**	ANKHD1	ankyrin repeat and KH domain containing 1
**216550_x_at**	ANKRD12	ankyrin repeat domain 12
**238851_at**	ANKRD13A	ankyrin repeat domain 13A
**1569607_s_at**	ANKRD20A1 /// ANKRD20A2 /// ANKRD20A3 /// ANKRD20A4 /// ANKRD20A5 /// C21orf81 /// LOC100132733 /// LOC284232 /// LOC644339	ankyrin repeat domain 20 family, member A1 /// ankyrin repeat domain 20 family, member A2 /// ankyrin repeat domain 20 family, member A3 /// ankyrin repeat domain 20 family, member A4 /// ankyrin repeat domain 20 family, member A5 /// ankyrin repeat domain 20 family, member A3 pseudogene /// similar to FLJ00310 protein /// ankyrin repeat domain 20 family, member A2 pseudogene /// similar to ANKRD20A2 protein
**214723_x_at**	ANKRD36	ankyrin repeat domain 36
**220940_at**	ANKRD36B	ankyrin repeat domain 36B
**230972_at**	ANKRD9	ankyrin repeat domain 9
**218910_at**	ANO10	anoctamin 10
**229313_at**	ANO5	anoctamin 5
**202888_s_at**	ANPEP	alanyl (membrane) aminopeptidase
**225524_at**	ANTXR2	anthrax toxin receptor 2
**208323_s_at**	ANXA13	annexin A13
**225771_at**	AP1G1	adaptor-related protein complex 1, gamma 1 subunit
**231714_s_at**	AP4B1	adaptor-related protein complex 4, beta 1 subunit
**220023_at**	APOB48R	apolipoprotein B48 receptor
**205216_s_at**	APOH	apolipoprotein H (beta-2-glycoprotein I)
**200602_at**	APP	amyloid beta (A4) precursor protein

**209047_at**	AQP1	aquaporin 1 (Colton blood group)
**39248_at**	AQP3	aquaporin 3 (Gill blood group)
**214102_at**	ARAP2	ArfGAP with RhoGAP domain, ankyrin repeat and PH domain 2
**232175_at**	ARF1	ADP-ribosylation factor 1
**201097_s_at**	ARF4	ADP-ribosylation factor 4
**203312_x_at**	ARF6	ADP-ribosylation factor 6
**241701_at**	ARHGAP21	Rho GTPase activating protein 21
**205068_s_at**	ARHGAP26	Rho GTPase activating protein 26
**229648_at**	ARHGAP32	Rho GTPase activating protein 32
**232567_at**	ARHGAP8	Rho GTPase activating protein 8
**1570511_at**	ARHGEF10L	Rho guanine nucleotide exchange factor (GEF) 10-like
**203263_s_at**	ARHGEF9	Cdc42 guanine nucleotide exchange factor (GEF) 9
**201658_at**	ARL1	ADP-ribosylation factor-like 1
**220468_at**	ARL14	ADP-ribosylation factor-like 14
**236966_at**	ARMC8	armadillo repeat containing 8
**222912_at**	ARRB1	arrestin, beta 1
**225283_at**	ARRDC4	arrestin domain containing 4
**238878_at**	ARX	aristaless related homeobox
**213902_at**	ASAH1	N-acylsphingosine amidohydrolase (acid ceramidase) 1
**218857_s_at**	ASRGL1	asparaginase like 1
**231825_x_at**	ATF7IP	activating transcription factor 7 interacting protein
**225883_at**	ATG16L2	ATG16 autophagy related 16-like 2 (S. cerevisiae)
**213115_at**	ATG4A	ATG4 autophagy related 4 homolog A (S. cerevisiae)
**220920_at**	ATP10B	ATPase, class V, type 10B
**207522_s_at**	ATP2A3	ATPase, Ca++ transporting, ubiquitous
**215716_s_at**	ATP2B1	ATPase, Ca++ transporting, plasma membrane 1
**206043_s_at**	ATP2C2	ATPase, Ca++ transporting, type 2C, member 2
**208972_s_at**	ATP5G1	ATP synthase, H+ transporting, mitochondrial F0 complex, subunit C1 (subunit 9)
**207335_x_at**	ATP5I	ATP synthase, H+ transporting, mitochondrial F0 complex, subunit E
**237400_at**	ATP5S	ATP synthase, H+ transporting, mitochondrial F0 complex, subunit s (factor B)
**208898_at**	ATP6V1D	ATPase, H+ transporting, lysosomal 34kDa, V1 subunit D
**213106_at**	ATP8A1	ATPase, aminophospholipid transporter (APLT), class I, type 8A, member 1
**214594_x_at**	ATP8B1	ATPase, aminophospholipid transporter, class I, type 8B, member 1
**214934_at**	ATP9B	ATPase, class II, type 9B
**218671_s_at**	ATPIF1	ATPase inhibitory factor 1
**210205_at**	B3GALT4	UDP-Gal:betaGlcNAc beta 1,3-galactosyltransferase, polypeptide 4
**1552834_at**	B3GNT6	UDP-GlcNAc:betaGal beta-1,3-N-acetylglucosaminyltransferase 6 (core 3 synthase)
**1555962_at**	B3GNT7	UDP-GlcNAc:betaGal beta-1,3-N-acetylglucosaminyltransferase 7
**229909_at**	B4GALNT3	beta-1,4-N-acetyl-galactosaminyl transferase 3
**212876_at**	B4GALT4	UDP-Gal:betaGlcNAc beta 1,4- galactosyltransferase, polypeptide 4
**202387_at**	BAG1	BCL2-associated athanogene
**214052_x_at**	BAT2L2	HLA-B associated transcript 2-like 2
**204032_at**	BCAR3	breast cancer anti-estrogen resistance 3
**204378_at**	BCAS1	breast carcinoma amplified sequence 1
**205263_at**	BCL10	B-cell CLL/lymphoma 10
**1559078_at**	BCL11A	B-cell CLL/lymphoma 11A (zinc finger protein)
**203685_at**	BCL2	B-cell CLL/lymphoma 2
**1555372_at**	BCL2L11	BCL2-like 11 (apoptosis facilitator)
**221241_s_at**	BCL2L14	BCL2-like 14 (apoptosis facilitator)

**236083_at**	BCL2L15	BCL2-like 15
**218285_s_at**	BDH2	3-hydroxybutyrate dehydrogenase, type 2
**205870_at**	BDKRB2	bradykinin receptor B2
**224227_s_at**	BDP1	B double prime 1, subunit of RNA polymerase III transcription initiation factor IIIB
**207432_at**	BEST2	bestrophin 2
**1552296_at**	BEST4	bestrophin 4
**229963_at**	BEX5	brain expressed, X-linked 5
**207655_s_at**	BLNK	B-cell linker
**202201_at**	BLVRB	biliverdin reductase B (flavin reductase (NADPH))
**205289_at**	BMP2	bone morphogenetic protein 2
**219546_at**	BMP2K	BMP2 inducible kinase
**218024_at**	BRP44L	brain protein 44-like
**224943_at**	BTBD7	BTB (POZ) domain containing 7
**217207_s_at**	BTNL3	butyrophilin-like 3
**220421_at**	BTNL8	butyrophilin-like 8
**225035_x_at**	BTNL8 /// LOC100288778 /// WASH1 /// WASH2P /// WASH3P /// WASH7P	butyrophilin-like 8 /// similar to WAS protein family homolog 1 /// WAS protein family homolog 1 /// WAS protein family homolog 2 pseudogene /// WAS protein family homolog 3 pseudogene /// WAS protein family homolog 7 pseudogene
**226340_x_at**	BTNL8 /// WASH1 /// WASH2P /// WASH3P /// WASH7P	butyrophilin-like 8 /// WAS protein family homolog 1 /// WAS protein family homolog 2 pseudogene /// WAS protein family homolog 3 pseudogene /// WAS protein family homolog 7 pseudogene
**202808_at**	C10orf26	chromosome 10 open reading frame 26
**225373_at**	C10orf54	chromosome 10 open reading frame 54
**227736_at**	C10orf99	chromosome 10 open reading frame 99
**219806_s_at**	C11orf75	chromosome 11 open reading frame 75
**242469_at**	C11orf93	Chromosome 11 open reading frame 93
**222613_at**	C12orf4	chromosome 12 open reading frame 4
**224719_s_at**	C12orf57	chromosome 12 open reading frame 57
**229520_s_at**	C14orf118	chromosome 14 open reading frame 118
**223239_at**	C14orf129	chromosome 14 open reading frame 129
**219563_at**	C14orf139	chromosome 14 open reading frame 139
**232814_x_at**	C14orf153	Chromosome 14 open reading frame 153
**218298_s_at**	C14orf159	chromosome 14 open reading frame 159
**229054_at**	C14orf181	chromosome 14 open reading frame 181
**202279_at**	C14orf2	chromosome 14 open reading frame 2
**238647_at**	C14orf28	chromosome 14 open reading frame 28
**237654_at**	C14orf50	chromosome 14 open reading frame 50
**228558_at**	C14orf80	chromosome 14 open reading frame 80
**224804_s_at**	C15orf17	chromosome 15 open reading frame 17
**242649_x_at**	C15orf21	Dresden prostate cancer 2
**232094_at**	C15orf29	chromosome 15 open reading frame 29
**223484_at**	C15orf48	chromosome 15 open reading frame 48
**203173_s_at**	C16orf62	chromosome 16 open reading frame 62
**205781_at**	C16orf7	chromosome 16 open reading frame 7
**238295_at**	C17orf42	Chromosome 17 open reading frame 42
**214696_at**	C17orf91	chromosome 17 open reading frame 91
**224493_x_at**	C18orf45	chromosome 18 open reading frame 45
**223181_at**	C18orf55	chromosome 18 open reading frame 55
**222266_at**	C19orf2	Chromosome 19 open reading frame 2

**223631_s_at**	C19orf33	chromosome 19 open reading frame 33
**230213_at**	C19orf43	chromosome 19 open reading frame 43
**232469_x_at**	C1orf191	chromosome 1 open reading frame 191
**223125_s_at**	C1orf21	chromosome 1 open reading frame 21
**1554246_at**	C1orf210	chromosome 1 open reading frame 210
**209006_s_at**	C1orf63	chromosome 1 open reading frame 63
**228004_at**	C20orf56	chromosome 20 open reading frame 56
**202217_at**	C21orf33	chromosome 21 open reading frame 33
**239208_s_at**	C21orf57	Chromosome 21 open reading frame 57
**1555231_a_at**	C21orf88	chromosome 21 open reading frame 88
**36552_at**	C2CD3	C2 calcium-dependent domain containing 3
**213143_at**	C2orf72	chromosome 2 open reading frame 72
**228195_at**	C2orf88	chromosome 2 open reading frame 88
**227867_at**	C2orf89	chromosome 2 open reading frame 89
**242447_at**	C3orf70	chromosome 3 open reading frame 70
**219450_at**	C4orf19	chromosome 4 open reading frame 19
**224990_at**	C4orf34	chromosome 4 open reading frame 34
**230424_at**	C5orf13	chromosome 5 open reading frame 13
**224707_at**	C5orf32	chromosome 5 open reading frame 32
**1560128_x_at**	C5orf56	Chromosome 5 open reading frame 56
**229070_at**	C6orf105	chromosome 6 open reading frame 105
**227456_s_at**	C6orf136	chromosome 6 open reading frame 136
**212913_at**	C6orf26	chromosome 6 open reading frame 26
**202992_at**	C7	complement component 7
**229146_at**	C7orf31	chromosome 7 open reading frame 31
**228600_x_at**	C7orf46	chromosome 7 open reading frame 46
**224890_s_at**	C7orf59	chromosome 7 open reading frame 59
**230903_s_at**	C8orf42	chromosome 8 open reading frame 42
**225603_s_at**	C8orf83	chromosome 8 open reading frame 83
**224458_at**	C9orf125	chromosome 9 open reading frame 125
**229964_at**	C9orf152	chromosome 9 open reading frame 152
**223697_x_at**	C9orf64	chromosome 9 open reading frame 64
**205950_s_at**	CA1	carbonic anhydrase I
**215867_x_at**	CA12	carbonic anhydrase XII
**209301_at**	CA2	carbonic anhydrase II
**207504_at**	CA7	carbonic anhydrase VII
**214933_at**	CACNA1A	calcium channel, voltage-dependent, P/Q type, alpha 1A subunit
**204811_s_at**	CACNA2D2	calcium channel, voltage-dependent, alpha 2/delta subunit 2
**1559419_at**	CACNB2	calcium channel, voltage-dependent, beta 2 subunit
**34726_at**	CACNB3	calcium channel, voltage-dependent, beta 3 subunit
**210817_s_at**	CALCOCO2	calcium binding and coiled-coil domain 2
**211984_at**	CALM1	calmodulin 1 (phosphorylase kinase, delta)
**64408_s_at**	CALML4	calmodulin-like 4
**214315_x_at**	CALR	calreticulin
**238908_at**	CALU	Calumenin
**235626_at**	CAMK1D	calcium/calmodulin-dependent protein kinase ID
**226959_at**	CAMK1D /// LOC283070	calcium/calmodulin-dependent protein kinase ID /// hypothetical LOC283070
**231793_s_at**	CAMK2D	calcium/calmodulin-dependent protein kinase II delta
**229163_at**	CAMK2N1	calcium/calmodulin-dependent protein kinase II inhibitor 1
**46323_at**	CANT1	calcium activated nucleotidase 1
**234709_at**	CAPN13	calpain 13

**208683_at**	CAPN2	calpain 2, (m/II) large subunit
**226292_at**	CAPN5	calpain 5
**229030_at**	CAPN8	calpain 8
**210641_at**	CAPN9	calpain 9
**37012_at**	CAPZB	capping protein (actin filament) muscle Z-line, beta
**1552701_a_at**	CARD16	caspase recruitment domain family, member 16
**1552703_s_at**	CARD16 /// CASP1	caspase recruitment domain family, member 16 /// caspase 1, apoptosis-related cysteine peptidase (interleukin 1, beta, convertase)
**224619_at**	CASC4	cancer susceptibility candidate 4
**219342_at**	CASD1	CAS1 domain containing 1
**206011_at**	CASP1	caspase 1, apoptosis-related cysteine peptidase (interleukin 1, beta, convertase)
**207181_s_at**	CASP7	caspase 7, apoptosis-related cysteine peptidase
**1561405_s_at**	CATSPER2	cation channel, sperm associated 2
**238549_at**	CBFA2T2	core-binding factor, runt domain, alpha subunit 2; translocated to, 2
**208056_s_at**	CBFA2T3	core-binding factor, runt domain, alpha subunit 2; translocated to, 3
**244052_at**	CBR4	carbonyl reductase 4
**225320_at**	CCDC109A	coiled-coil domain containing 109A
**1553542_at**	CCDC125	coiled-coil domain containing 125
**225017_at**	CCDC14	coiled-coil domain containing 14
**237475_x_at**	CCDC152	coiled-coil domain containing 152
**235095_at**	CCDC64B	coiled-coil domain containing 64B
**220180_at**	CCDC68	coiled-coil domain containing 68
**232489_at**	CCDC76	coiled-coil domain containing 76
**222809_x_at**	CCDC85C	coiled-coil domain containing 85C
**210390_s_at**	CCL14-CCL15 /// CCL15	CCL14-CCL15 read-through transcript /// chemokine (C-C motif) ligand 15
**224027_at**	CCL28	chemokine (C-C motif) ligand 28
**201743_at**	CD14	CD14 molecule
**219669_at**	CD177	CD177 molecule
**205789_at**	CD1D	CD1d molecule
**200983_x_at**	CD59	CD59 molecule, complement regulatory protein
**200663_at**	CD63	CD63 molecule
**208728_s_at**	CDC42	cell division cycle 42 (GTP binding protein, 25kDa)
**214464_at**	CDC42BPA	CDC42 binding protein kinase alpha (DMPK-like)
**227850_x_at**	CDC42EP5	CDC42 effector protein (Rho GTPase binding) 5
**219796_s_at**	CDHR5	cadherin-related family member 5
**203468_at**	CDK10	cyclin-dependent kinase 10
**232266_x_at**	CDK13	Cyclin-dependent kinase 13
**229468_at**	CDK3	cyclin-dependent kinase 3
**235512_at**	CDKL1	cyclin-dependent kinase-like 1 (CDC2-related kinase)
**236313_at**	CDKN2B	cyclin-dependent kinase inhibitor 2B (p15, inhibits CDK4)
**204159_at**	CDKN2C	cyclin-dependent kinase inhibitor 2C (p18, inhibits CDK4)
**205709_s_at**	CDS1	CDP-diacylglycerol synthase (phosphatidate cytidylyltransferase) 1
**206430_at**	CDX1	caudal type homeobox 1
**206199_at**	CEACAM7	carcinoembryonic antigen-related cell adhesion molecule 7
**219036_at**	CEP70	centrosomal protein 70kDa
**209667_at**	CES2	carboxylesterase 2 (intestine, liver)
**234008_s_at**	CES3	carboxylesterase 3
**205382_s_at**	CFD	complement factor D (adipsin)
**239629_at**	CFLAR	CASP8 and FADD-like apoptosis regulator
**204605_at**	CGRRF1	cell growth regulator with ring finger domain 1

**224932_at**	CHCHD10	coiled-coil-helix-coiled-coil-helix domain containing 10
**244443_at**	CHD2	Chromodomain helicase DNA binding protein 2
**208807_s_at**	CHD3	chromodomain helicase DNA binding protein 3
**212616_at**	CHD9	chromodomain helicase DNA binding protein 9
**218803_at**	CHFR	checkpoint with forkhead and ring finger domains
**204697_s_at**	CHGA	chromogranin A (parathyroid secretory protein 1)
**204260_at**	CHGB	chromogranin B (secretogranin 1)
**210069_at**	CHKB-CPT1B /// CPT1B	choline kinase-like, carnitine palmitoyltransferase 1B (muscle) transcription unit /// carnitine palmitoyltransferase 1B (muscle)
**218571_s_at**	CHMP4A	chromatin modifying protein 4A
**214665_s_at**	CHP	calcium binding protein P22
**206149_at**	CHP2	calcineurin B homologous protein 2
**221164_x_at**	CHST5	carbohydrate (N-acetylglucosamine 6-O) sulfotransferase 5
**200810_s_at**	CIRBP	cold inducible RNA binding protein
**225191_at**	CIRBP	cold inducible RNA binding protein
**209357_at**	CITED2	Cbp/p300-interacting transactivator, with Glu/Asp-rich carboxy-terminal domain, 2
**200999_s_at**	CKAP4	cytoskeleton-associated protein 4
**200884_at**	CKB	creatine kinase, brain
**202712_s_at**	CKMT1A /// CKMT1B	creatine kinase, mitochondrial 1A /// creatine kinase, mitochondrial 1B
**210107_at**	CLCA1	chloride channel accessory 1
**220026_at**	CLCA4	chloride channel accessory 4
**214598_at**	CLDN8	claudin 8
**227742_at**	CLIC6	chloride intracellular channel 6
**201769_at**	CLINT1	clathrin interactor 1
**242287_at**	CLIP1	CAP-GLY domain containing linker protein 1
**213839_at**	CLMN	calmin (calponin-like, transmembrane)
**229777_at**	CLRN3	clarin 3
**205172_x_at**	CLTB	clathrin, light chain B
**205518_s_at**	CMAH	cytidine monophosphate-N-acetylneuraminic acid hydroxylase (CMP-N-acetylneuraminate monooxygenase) pseudogene
**241065_x_at**	CMAS	Cytidine monophosphate N-acetylneuraminic acid synthetase
**234981_x_at**	CMBL	carboxymethylenebutenolidase homolog (Pseudomonas)
**224998_at**	CMTM4	CKLF-like MARVEL transmembrane domain containing 4
**217752_s_at**	CNDP2	CNDP dipeptidase 2 (metallopeptidase M20 family)
**1554522_at**	CNNM2	cyclin M2
**218900_at**	CNNM4	cyclin M4
**229831_at**	CNTN3	contactin 3 (plasmacytoma associated)
**229084_at**	CNTN4	contactin 4
**243608_at**	COG2	component of oligomeric golgi complex 2
**213379_at**	COQ2	coenzyme Q2 homolog, prenyltransferase (yeast)
**224583_at**	COTL1	coactosin-like 1 (Dictyostelium)
**213758_at**	COX4I1	cytochrome c oxidase subunit IV isoform 1
**229426_at**	COX5A	cytochrome c oxidase subunit Va
**201134_x_at**	COX7C	cytochrome c oxidase subunit VIIc
**206100_at**	CPM	carboxypeptidase M
**241706_at**	CPNE8	copine VIII
**203633_at**	CPT1A	carnitine palmitoyltransferase 1A (liver)
**204264_at**	CPT2	carnitine palmitoyltransferase 2
**213059_at**	CREB3L1	cAMP responsive element binding protein 3-like 1
**226455_at**	CREB3L4	cAMP responsive element binding protein 3-like 4

**218358_at**	CRELD2	cysteine-rich with EGF-like domains 2
**205081_at**	CRIP1	cysteine-rich protein 1 (intestinal)
**204573_at**	CROT	carnitine O-octanoyltransferase
**214030_at**	CRYBG3	beta-gamma crystallin domain containing 3
**205489_at**	CRYM	crystallin, mu
**1552347_at**	CRYZL1	crystallin, zeta (quinone reductase)-like 1
**210835_s_at**	CTBP2	C-terminal binding protein 2
**201906_s_at**	CTDSPL	CTD (carboxy-terminal domain, RNA polymerase II, polypeptide A) small phosphatase-like
**217127_at**	CTH	cystathionase (cystathionine gamma-lyase)
**208407_s_at**	CTNND1	catenin (cadherin-associated protein), delta 1
**205927_s_at**	CTSE	cathepsin E
**232617_at**	CTSS	cathepsin S
**201059_at**	CTTN	cortactin
**237040_at**	CWF19L2	CWF19-like 2, cell cycle control (S. pombe)
**220723_s_at**	CWH43	cell wall biogenesis 43 C-terminal homolog (S. cerevisiae)
**214677_x_at**	CYAT1 /// IGLV1-44	cyclosporin A transporter 1 /// immunoglobulin lambda variable 1-44
**244546_at**	CYCS	cytochrome c, somatic
**213295_at**	CYLD	cylindromatosis (turban tumor syndrome)
**1556006_s_at**	CSNK1A1	Casein kinase 1, alpha 1
**235017_s_at**	CSRNP3	cysteine-serine-rich nuclear protein 3
**200621_at**	CSRP1	cysteine and glycine-rich protein 1
**229665_at**	CSTF3	cleavage stimulation factor, 3' pre-RNA, subunit 3, 77kDa
**228738_at**	D2HGDH	D-2-hydroxyglutarate dehydrogenase
**216060_s_at**	DAAM1	dishevelled associated activator of morphogenesis 1
**219290_x_at**	DAPP1	dual adaptor of phosphotyrosine and 3-phosphoinositides
**205370_x_at**	DBT	dihydrolipoamide branched chain transacylase E2
**243318_at**	DCAF8	DDB1 and CUL4 associated factor 8
**203409_at**	DDB2	damage-specific DNA binding protein 2, 48kDa
**213998_s_at**	DDX17	DEAD (Asp-Glu-Ala-Asp) box polypeptide 17
**200702_s_at**	DDX24	DEAD (Asp-Glu-Ala-Asp) box polypeptide 24
**227485_at**	DDX26B	DEAD/H (Asp-Glu-Ala-Asp/His) box polypeptide 26B
**223662_x_at**	DDX59	DEAD (Asp-Glu-Ala-Asp) box polypeptide 59
**1564164_at**	DENND1B	DENN/MADD domain containing 1B
**53991_at**	DENND2A	DENN/MADD domain containing 2A
**221081_s_at**	DENND2D	DENN/MADD domain containing 2D
**91617_at**	DGCR8	DiGeorge syndrome critical region gene 8
**203385_at**	DGKA	diacylglycerol kinase, alpha 80kDa
**218547_at**	DHDDS	dehydrodolichyl diphosphate synthase
**213279_at**	DHRS1	dehydrogenase/reductase (SDR family) member 1
**218756_s_at**	DHRS11	dehydrogenase/reductase (SDR family) member 11
**218021_at**	DHRS4 /// DHRS4L2	dehydrogenase/reductase (SDR family) member 4 /// dehydrogenase/reductase (SDR family) member 4 like 2
**219799_s_at**	DHRS9	dehydrogenase/reductase (SDR family) member 9
**213229_at**	DICER1	dicer 1, ribonuclease type III
**215529_x_at**	DIP2A	DIP2 disco-interacting protein 2 homolog A (Drosophila)
**229579_s_at**	DISP2	dispatched homolog 2 (Drosophila)
**213546_at**	DKFZP586I1420	hypothetical protein DKFZp586I1420
**214247_s_at**	DKK3	dickkopf homolog 3 (Xenopus laevis)
**230229_at**	DLG1	Discs, large homolog 1 (Drosophila)
**233056_x_at**	DLGAP4	discs, large (Drosophila) homolog-associated protein 4
**224215_s_at**	DLL1	delta-like 1 (Drosophila)

**215210_s_at**	DLST	dihydrolipoamide S-succinyltransferase (E2 component of 2-oxo-glutarate complex)
**212820_at**	DMXL2	Dmx-like 2
**223054_at**	DNAJB11	DnaJ (Hsp40) homolog, subfamily B, member 11
**222620_s_at**	DNAJC1	DnaJ (Hsp40) homolog, subfamily C, member 1
**223722_at**	DNAJC12	DnaJ (Hsp40) homolog, subfamily C, member 12
**244193_at**	DNAJC22	DnaJ (Hsp40) homolog, subfamily C, member 22
**242562_at**	DNAJC24	DnaJ (Hsp40) homolog, subfamily C, member 24
**228622_s_at**	DNAJC4	DnaJ (Hsp40) homolog, subfamily C, member 4
**57739_at**	DND1	dead end homolog 1 (zebrafish)
**230263_s_at**	DOCK5	Dedicator of cytokinesis 5
**209691_s_at**	DOK4	docking protein 4
**221817_at**	DOLPP1	dolichyl pyrophosphate phosphatase 1
**236442_at**	DPF3	D4, zinc and double PHD fingers, family 3
**222041_at**	DPH1 /// OVCA2	DPH1 homolog (S. cerevisiae) /// ovarian tumor suppressor candidate 2
**228598_at**	DPP10	dipeptidyl-peptidase 10 (non-functional)
**203716_s_at**	DPP4	dipeptidyl-peptidase 4
**236055_at**	DQX1	DEAQ box RNA-dependent ATPase 1
**204750_s_at**	DSC2	desmocollin 2
**236649_at**	DTWD1	DTW domain containing 1
**224616_at**	DYNC1LI2	dynein, cytoplasmic 1, light intermediate chain 2
**228361_at**	E2F2	E2F transcription factor 2
**223243_s_at**	EDEM3	ER degradation enhancer, mannosidase alpha-like 3
**209058_at**	EDF1	endothelial differentiation-related factor 1
**225275_at**	EDIL3	EGF-like repeats and discoidin I-like domains 3
**208399_s_at**	EDN3	endothelin 3
**228752_at**	EFCAB4B	EF-hand calcium binding domain 4B
**220591_s_at**	EFHC2	EF-hand domain (C-terminal) containing 2
**222483_at**	EFHD2	EF-hand domain family, member D2
**214036_at**	EFNA5	ephrin-A5
**202669_s_at**	EFNB2	ephrin-B2
**232360_at**	EHF	ets homologous factor
**205222_at**	EHHADH	enoyl-CoA, hydratase/3-hydroxyacyl CoA dehydrogenase
**214805_at**	EIF4A1	Eukaryotic translation initiation factor 4A1
**201936_s_at**	EIF4G3	eukaryotic translation initiation factor 4 gamma, 3
**213757_at**	EIF5A	Eukaryotic translation initiation factor 5A
**201510_at**	ELF3	E74-like factor 3 (ets domain transcription factor, epithelial-specific)
**219518_s_at**	ELL3 /// SERINC4	elongation factor RNA polymerase II-like 3 /// serine incorporator 4
**210868_s_at**	ELOVL6	ELOVL family member 6, elongation of long chain fatty acids (FEN1/Elo2, SUR4/Elo3-like, yeast)
**235623_at**	ELP2	Elongation protein 2 homolog (S. cerevisiae)
**1569868_s_at**	EME2	essential meiotic endonuclease 1 homolog 2 (S. pombe)
**204398_s_at**	EML2	echinoderm microtubule associated protein like 2
**204975_at**	EMP2	epithelial membrane protein 2
**212573_at**	ENDOD1	endonuclease domain containing 1
**204142_at**	ENOSF1	enolase superfamily member 1
**205757_at**	ENTPD5	ectonucleoside triphosphate diphosphohydrolase 5
**230629_s_at**	EP400	E1A binding protein p400
**212681_at**	EPB41L3	erythrocyte membrane protein band 4.1-like 3
**228256_s_at**	EPB41L4A	erythrocyte membrane protein band 4.1 like 4A
**223426_s_at**	EPB41L4B	erythrocyte membrane protein band 4.1 like 4B

**206114_at**	EPHA4	EPH receptor A4
**209368_at**	EPHX2	epoxide hydrolase 2, cytoplasmic
**202909_at**	EPM2AIP1	EPM2A (laforin) interacting protein 1
**223895_s_at**	EPN3	epsin 3
**202414_at**	ERCC5	excision repair cross-complementing rodent repair deficiency, complementation group 5
**214372_x_at**	ERN2	endoplasmic reticulum to nucleus signaling 2
**235645_at**	ESCO1	establishment of cohesion 1 homolog 1 (S. cerevisiae)
**225846_at**	ESRP1	epithelial splicing regulatory protein 1
**229223_at**	ESRP2	epithelial splicing regulatory protein 2
**201573_s_at**	ETF1	eukaryotic translation termination factor 1
**202942_at**	ETFB	electron-transfer-flavoprotein, beta polypeptide
**205530_at**	ETFDH	electron-transferring-flavoprotein dehydrogenase
**204034_at**	ETHE1	ethylmalonic encephalopathy 1
**224833_at**	ETS1	v-ets erythroblastosis virus E26 oncogene homolog 1 (avian)
**221911_at**	ETV1	ets variant 1
**208297_s_at**	EVI5	ecotropic viral integration site 5
**229966_at**	EWSR1	Ewing sarcoma breakpoint region 1
**214802_at**	EXOC7	exocyst complex component 7
**213929_at**	EXPH5	exophilin 5
**230183_at**	EXT1	exostosin 1
**213506_at**	F2RL1	coagulation factor II (thrombin) receptor-like 1
**204231_s_at**	FAAH	fatty acid amide hydrolase
**205892_s_at**	FABP1	fatty acid binding protein 1, liver
**202345_s_at**	FABP5	fatty acid binding protein 5 (psoriasis-associated)
**227960_s_at**	FAHD1	fumarylacetoacetate hydrolase domain containing 1
**223058_at**	FAM107B	family with sequence similarity 107, member B
**226697_at**	FAM114A1	family with sequence similarity 114, member A1
**226475_at**	FAM118A	family with sequence similarity 118, member A
**230009_at**	FAM118B	family with sequence similarity 118, member B
**239391_at**	FAM120AOS	Family with sequence similarity 120A opposite strand
**220720_x_at**	FAM128B	family with sequence similarity 128, member B
**222291_at**	FAM149A	family with sequence similarity 149, member A
**238018_at**	FAM150B	family with sequence similarity 150, member B
**223637_s_at**	FAM160A2	family with sequence similarity 160, member A2
**242608_x_at**	FAM161B	Family with sequence similarity 161, member B
**220942_x_at**	FAM162A	family with sequence similarity 162, member A
**235850_at**	FAM162A	family with sequence similarity 162, member A
**51158_at**	FAM174B	family with sequence similarity 174, member B
**227029_at**	FAM177A1	family with sequence similarity 177, member A1
**229762_at**	FAM200A	Family with sequence similarity 200, member A
**226722_at**	FAM20C	family with sequence similarity 20, member C
**212929_s_at**	FAM21A /// FAM21B /// FAM21C /// FAM21D	family with sequence similarity 21, member A /// family with sequence similarity 21, member B /// family with sequence similarity 21, member C /// family with sequence similarity 21, member D
**227676_at**	FAM3D	family with sequence similarity 3, member D
**224973_at**	FAM46A	family with sequence similarity 46, member A
**220306_at**	FAM46C	family with sequence similarity 46, member C
**1561387_a_at**	FAM55A	family with sequence similarity 55, member A
**220645_at**	FAM55D	family with sequence similarity 55, member D
**228115_at**	FAM59A	family with sequence similarity 59, member A
**217562_at**	FAM5C	family with sequence similarity 5, member C

**226062_x_at**	FAM63A	family with sequence similarity 63, member A
**226356_at**	FAM73B	family with sequence similarity 73, member B
**235349_at**	FAM82A1	family with sequence similarity 82, member A1
**220312_at**	FAM83E	family with sequence similarity 83, member E
**224866_at**	FAR1	fatty acyl CoA reductase 1
**239108_at**	FAR2	Fatty acyl CoA reductase 2
**232063_x_at**	FARSB	phenylalanyl-tRNA synthetase, beta subunit
**204781_s_at**	FAS	Fas (TNF receptor superfamily, member 6)
**227203_at**	FBXL17	F-box and leucine-rich repeat protein 17
**242829_x_at**	FBXL3	F-box and leucine-rich repeat protein 3
**241762_at**	FBXO32	F-box protein 32
**215600_x_at**	FBXW12	F-box and WD repeat domain containing 12
**203240_at**	FCGBP	Fc fragment of IgG binding protein
**241954_at**	FDFT1	Farnesyl-diphosphate farnesyltransferase 1
**230559_x_at**	FGD4	FYVE, RhoGEF and PH domain containing 4
**206404_at**	FGF9	fibroblast growth factor 9 (glia-activating factor)
**205014_at**	FGFBP1	fibroblast growth factor binding protein 1
**203638_s_at**	FGFR2	fibroblast growth factor receptor 2
**208228_s_at**	FGFR2	fibroblast growth factor receptor 2
**204379_s_at**	FGFR3	fibroblast growth factor receptor 3
**201540_at**	FHL1	four and a half LIM domains 1
**203391_at**	FKBP2	FK506 binding protein 2, 13kDa
**218003_s_at**	FKBP3	FK506 binding protein 3, 25kDa
**224840_at**	FKBP5	FK506 binding protein 5
**241627_x_at**	FLJ10357	protein SOLO
**226706_at**	FLJ23867 /// QSOX1	hypothetical protein FLJ23867 /// quiescin Q6 sulfhydryl oxidase 1
**243309_at**	FLJ27352	hypothetical LOC145788
**1560010_a_at**	FLJ32063	hypothetical LOC150538
**227593_at**	FLJ37453	hypothetical LOC729614
**220352_x_at**	FLJ42627	hypothetical LOC645644
**230404_at**	FLJ44606	glutaredoxin-like protein YDR286C homolog
**1566558_x_at**	FLJ90757	hypothetical LOC440465
**208614_s_at**	FLNB	filamin B, beta
**205776_at**	FMO5	flavin containing monooxygenase 5
**230389_at**	FNBP1	formin binding protein 1
**229272_at**	FNBP4	formin binding protein 4
**225922_at**	FNIP2	folliculin interacting protein 2
**237086_at**	FOXA1	Forkhead box A1
**223287_s_at**	FOXP1	forkhead box P1
**243278_at**	FOXP2	forkhead box P2
**226145_s_at**	FRAS1	Fraser syndrome 1
**229893_at**	FRMD3	FERM domain containing 3
**213056_at**	FRMD4B	FERM domain containing 4B
**212548_s_at**	FRYL	FRY-like
**203697_at**	FRZB	frizzled-related protein
**214211_at**	FTH1	ferritin, heavy polypeptide 1
**212847_at**	FUBP1	Far upstream element (FUSE) binding protein 1
**202838_at**	FUCA1	fucosidase, alpha-L- 1, tissue
**202489_s_at**	FXYD3	FXYD domain containing ion transport regulator 3
**211795_s_at**	FYB	FYN binding protein
**204451_at**	FZD1	frizzled homolog 1 (Drosophila)
**221245_s_at**	FZD5	frizzled homolog 5 (Drosophila)

**227405_s_at**	FZD8	frizzled homolog 8 (Drosophila)
**214987_at**	GAB1	GRB2-associated binding protein 1
**1557122_s_at**	GABRB2	gamma-aminobutyric acid (GABA) A receptor, beta 2
**40225_at**	GAK	cyclin G associated kinase
**234974_at**	GALM	galactose mutarotase (aldose 1-epimerase)
**201724_s_at**	GALNT1	UDP-N-acetyl-alpha-D-galactosamine:polypeptide N-acetylgalactosaminyltransferase 1 (GalNAc-T1)
**218885_s_at**	GALNT12	UDP-N-acetyl-alpha-D-galactosamine:polypeptide N-acetylgalactosaminyltransferase 12 (GalNAc-T12)
**236129_at**	GALNT5	UDP-N-acetyl-alpha-D-galactosamine:polypeptide N-acetylgalactosaminyltransferase 5 (GalNAc-T5)
**222587_s_at**	GALNT7	UDP-N-acetyl-alpha-D-galactosamine:polypeptide N-acetylgalactosaminyltransferase 7 (GalNAc-T7)
**228238_at**	GAS5	growth arrest-specific 5 (non-protein coding)
**224627_at**	GBA2	glucosidase, beta (bile acid) 2
**222943_at**	GBA3	glucosidase, beta, acid 3 (cytosolic)
**210589_s_at**	GBAP1	glucosidase, beta, acid pseudogene 1
**206422_at**	GCG	glucagon
**230788_at**	GCNT2	glucosaminyl (N-acetyl) transferase 2, I-branching enzyme (I blood group)
**219508_at**	GCNT3	glucosaminyl (N-acetyl) transferase 3, mucin type
**219722_s_at**	GDPD3	glycerophosphodiester phosphodiesterase domain containing 3
**236225_at**	GGT6	gamma-glutamyltransferase 6
**207525_s_at**	GIPC1	GIPC PDZ domain containing family, member 1
**223278_at**	GJB2	gap junction protein, beta 2, 26kDa
**230360_at**	GLDN	gliomedin
**225604_s_at**	GLIPR2	GLI pathogenesis-related 2
**229435_at**	GLIS3	GLIS family zinc finger 3
**221932_s_at**	GLRX5	glutaredoxin 5
**219267_at**	GLTP	glycolipid transfer protein
**219920_s_at**	GMPPB	GDP-mannose pyrophosphorylase B
**213766_x_at**	GNA11	guanine nucleotide binding protein (G protein), alpha 11 (Gq class)
**227692_at**	GNAI1	guanine nucleotide binding protein (G protein), alpha inhibiting activity polypeptide 1
**202615_at**	GNAQ	Guanine nucleotide binding protein (G protein), q polypeptide
**222034_at**	GNB2L1	Guanine nucleotide binding protein (G protein), beta polypeptide 2-like 1
**205042_at**	GNE	glucosamine (UDP-N-acetyl)-2-epimerase/N-acetylmannosamine kinase
**224964_s_at**	GNG2	guanine nucleotide binding protein (G protein), gamma 2
**240106_at**	GNPTAB	N-acetylglucosamine-1-phosphate transferase, alpha and beta subunits
**35436_at**	GOLGA2	golgin A2
**233198_at**	GOLGA2B	golgin A2 family, member B
**202106_at**	GOLGA3	golgin A3
**215203_at**	GOLGA4	golgin A4
**208798_x_at**	GOLGA8A	golgin A8 family, member A
**217771_at**	GOLM1	golgi membrane protein 1
**208813_at**	GOT1	glutamic-oxaloacetic transaminase 1, soluble (aspartate aminotransferase 1)
**206681_x_at**	GP2	glycoprotein 2 (zymogen granule membrane)
**205929_at**	GPA33	glycoprotein A33 (transmembrane)
**1556126_s_at**	GPATCH2	G patch domain containing 2
**212510_at**	GPD1L	glycerol-3-phosphate dehydrogenase 1-like

**240856_at**	GPR120	G protein-coupled receptor 120
**210473_s_at**	GPR125	G protein-coupled receptor 125
**232350_x_at**	GPR161	G protein-coupled receptor 161
**206747_at**	GPRIN2	G protein regulated inducer of neurite outgrowth 2
**239595_at**	GPX2	glutathione peroxidase 2 (gastrointestinal)
**227628_at**	GPX8	glutathione peroxidase 8 (putative)
**219313_at**	GRAMD1C	GRAM domain containing 1C
**238049_at**	GRAMD3	GRAM domain containing 3
**212856_at**	GRAMD4	GRAM domain containing 4
**219233_s_at**	GSDMB	gasdermin B
**200696_s_at**	GSN	gelsolin
**240452_at**	GSPT1	G1 to S phase transition 1
**225609_at**	GSR	glutathione reductase
**203924_at**	GSTA1	glutathione S-transferase alpha 1
**235405_at**	GSTA4	glutathione S-transferase alpha 4
**217751_at**	GSTK1	glutathione S-transferase kappa 1
**209531_at**	GSTZ1	glutathione transferase zeta 1
**222104_x_at**	GTF2H3	general transcription factor IIH, polypeptide 3, 34kDa
**225083_at**	GTF3C6	general transcription factor IIIC, polypeptide 6, alpha 35kDa
**211040_x_at**	GTSE1	G-2 and S-phase expressed 1
**207003_at**	GUCA2A	guanylate cyclase activator 2A (guanylin)
**215599_at**	GUSBP3	glucuronidase, beta pseudogene 3
**201007_at**	HADHB	hydroxyacyl-CoA dehydrogenase/3-ketoacyl-CoA thiolase/enoyl-CoA hydratase (trifunctional protein), beta subunit
**205523_at**	HAPLN1	hyaluronan and proteoglycan link protein 1
**220071_x_at**	HAUS2	HAUS augmin-like complex, subunit 2
**209524_at**	HDGFRP3	hepatoma-derived growth factor, related protein 3
**242349_at**	HECTD1	HECT domain containing 1
**242601_at**	HEPACAM2	HEPACAM family member 2
**203903_s_at**	HEPH	hephaestin
**231928_at**	HES2	hairy and enhancer of split 2 (Drosophila)
**205221_at**	HGD	homogentisate 1,2-dioxygenase
**213374_x_at**	HIBCH	3-hydroxyisobutyryl-CoA hydrolase
**200989_at**	HIF1A	hypoxia inducible factor 1, alpha subunit (basic helix-loop-helix transcription factor)
**217845_x_at**	HIGD1A	HIG1 hypoxia inducible domain family, member 1A
**1555960_at**	HINT1	histidine triad nucleotide binding protein 1
**236278_at**	HIST1H3E	Histone cluster 1, H3e
**202934_at**	HK2	hexokinase 2
**202772_at**	HMGCL	3-hydroxymethyl-3-methylglutaryl-CoA lyase
**204607_at**	HMGCS2	3-hydroxy-3-methylglutaryl-CoA synthase 2 (mitochondrial)
**221606_s_at**	HMGN5	high-mobility group nucleosome binding domain 5
**205313_at**	HNF1B	HNF1 homeobox B
**222040_at**	HNRNPA1	heterogeneous nuclear ribonucleoprotein A1
**1555653_at**	HNRNPA3 /// HNRNPA3P1	heterogeneous nuclear ribonucleoprotein A3 /// heterogeneous nuclear ribonucleoprotein A3 pseudogene 1
**213359_at**	HNRNPD	Heterogeneous nuclear ribonucleoprotein D (AU-rich element RNA binding protein 1, 37kDa)
**214918_at**	HNRNPM	heterogeneous nuclear ribonucleoprotein M
**214639_s_at**	HOXA1	homeobox A1
**230666_at**	HOXA11AS	HOXA11 antisense RNA (non-protein coding)
**213844_at**	HOXA5	homeobox A5

**235753_at**	HOXA7	homeobox A7
**230105_at**	HOXB13	homeobox B13
**205601_s_at**	HOXB5	homeobox B5
**205366_s_at**	HOXB6	homeobox B6
**205975_s_at**	HOXD1	homeobox D1
**236681_at**	HOXD13	homeobox D13
**205522_at**	HOXD4 /// MIR10B	homeobox D4 /// microRNA 10b
**231906_at**	HOXD8	homeobox D8
**1554251_at**	HP1BP3	heterochromatin protein 1, binding protein 3
**206726_at**	HPGDS	hematopoietic prostaglandin D synthase
**222881_at**	HPSE	heparanase
**235496_at**	HRCT1	histidine rich carboxyl terminus 1
**232594_at**	HSBP1L1	heat shock factor binding protein 1-like 1
**204130_at**	HSD11B2	hydroxysteroid (11-beta) dehydrogenase 2
**217989_at**	HSD17B11	hydroxysteroid (17-beta) dehydrogenase 11
**204818_at**	HSD17B2	hydroxysteroid (17-beta) dehydrogenase 2
**216449_x_at**	HSP90B1	heat shock protein 90kDa beta (Grp94), member 1
**200799_at**	HSPA1A	heat shock 70kDa protein 1A
**211538_s_at**	HSPA2	heat shock 70kDa protein 2
**201565_s_at**	ID2	inhibitor of DNA binding 2, dominant negative helix-loop-helix protein
**213931_at**	ID2 /// ID2B	inhibitor of DNA binding 2, dominant negative helix-loop-helix protein /// inhibitor of DNA binding 2B, dominant negative helix-loop-helix protein (pseudogene)
**1558956_s_at**	IFT80	intraflagellar transport 80 homolog (Chlamydomonas)
**217022_s_at**	IGH@ /// IGHA1 /// IGHA2 /// LOC100126583	immunoglobulin heavy locus /// immunoglobulin heavy constant alpha 1 /// immunoglobulin heavy constant alpha 2 (A2m marker) /// hypothetical LOC100126583
**212592_at**	IGJ	immunoglobulin J polypeptide, linker protein for immunoglobulin alpha and mu polypeptides
**214836_x_at**	IGK@ /// IGKC	immunoglobulin kappa locus /// immunoglobulin kappa constant
**209138_x_at**	IGL@	Immunoglobulin lambda locus
**215946_x_at**	IGLL3	immunoglobulin lambda-like polypeptide 3
**202421_at**	IGSF3	immunoglobulin superfamily, member 3
**231929_at**	IKZF2	IKAROS family zinc finger 2 (Helios)
**202948_at**	IL1R1	interleukin 1 receptor, type I
**205403_at**	IL1R2	interleukin 1 receptor, type II
**205945_at**	IL6R	interleukin 6 receptor
**226333_at**	IL6R	interleukin 6 receptor
**235583_at**	ILDR1	immunoglobulin-like domain containing receptor 1
**203011_at**	IMPA1	inositol(myo)-1(or 4)-monophosphatase 1
**203126_at**	IMPA2	inositol(myo)-1(or 4)-monophosphatase 2
**218144_s_at**	INF2	inverted formin, FH2 and WH2 domain containing
**221091_at**	INSL5	insulin-like 5
**206502_s_at**	INSM1	insulinoma-associated 1
**233193_x_at**	INTS4	integrator complex subunit 4
**223165_s_at**	IP6K2	inositol hexakisphosphate kinase 2
**203474_at**	IQGAP2	IQ motif containing GTPase activating protein 2
**206104_at**	ISL1	ISL LIM homeobox 1
**232707_at**	ISX	intestine-specific homeobox
**223597_at**	ITLN1	intelectin 1 (galactofuranose binding)
**202746_at**	ITM2A	integral membrane protein 2A
**221004_s_at**	ITM2C	integral membrane protein 2C
**210740_s_at**	ITPK1	inositol 1,3,4-triphosphate 5/6 kinase

**225311_at**	IVD	isovaleryl-CoA dehydrogenase
**201362_at**	IVNS1ABP	influenza virus NS1A binding protein
**203845_at**	KAT2B	K(lysine) acetyltransferase 2B
**220727_at**	KCNK10	potassium channel, subfamily K, member 10
**221584_s_at**	KCNMA1	potassium large conductance calcium-activated channel, subfamily M, alpha member 1
**226245_at**	KCTD1	potassium channel tetramerisation domain containing 1
**212188_at**	KCTD12	potassium channel tetramerisation domain containing 12
**222668_at**	KCTD15	potassium channel tetramerisation domain containing 15
**200699_at**	KDELR2	KDEL (Lys-Asp-Glu-Leu) endoplasmic reticulum protein retention receptor 2
**227056_at**	KIAA0141	KIAA0141
**213242_x_at**	KIAA0284	KIAA0284
**214295_at**	KIAA0485	hypothetical LOC57235
**204546_at**	KIAA0513	KIAA0513
**212456_at**	KIAA0664	KIAA0664
**207305_s_at**	KIAA1012	KIAA1012
**212779_at**	KIAA1109	KIAA1109
**227230_s_at**	KIAA1211	KIAA1211
**232046_at**	KIAA1217	KIAA1217
**1562063_x_at**	KIAA1245 /// LOC200030 /// NBPF1 /// NBPF10 /// NBPF11 /// NBPF8 /// NBPF9	KIAA1245 /// neuroblastoma breakpoint family, member 11-like /// neuroblastoma breakpoint family, member 1 /// neuroblastoma breakpoint family, member 10 /// neuroblastoma breakpoint family, member 11 /// neuroblastoma breakpoint family, member 8 /// neuroblastoma breakpoint family, member 9
**243589_at**	KIAA1267 /// LOC100294337	KIAA1267 /// hypothetical protein LOC100294337
**221874_at**	KIAA1324	KIAA1324
**225327_at**	KIAA1370	KIAA1370
**235956_at**	KIAA1377	KIAA1377
**225508_at**	KIAA1468	KIAA1468
**233893_s_at**	KIAA1530	KIAA1530
**232030_at**	KIAA1632	KIAA1632
**1569302_at**	KIAA1731	KIAA1731
**220777_at**	KIF13A	kinesin family member 13A
**232083_at**	KIF16B	kinesin family member 16B
**238477_at**	KIF1C	kinesin family member 1C
**203129_s_at**	KIF5C	kinesin family member 5C
**226792_s_at**	KIFC2	kinesin family member C2
**205051_s_at**	KIT	v-kit Hardy-Zuckerman 4 feline sarcoma viral oncogene homolog
**226534_at**	KITLG	KIT ligand
**221841_s_at**	KLF4	Kruppel-like factor 4 (gut)
**209211_at**	KLF5	Kruppel-like factor 5 (intestinal)
**203542_s_at**	KLF9	Kruppel-like factor 9
**221219_s_at**	KLHDC4	kelch domain containing 4
**206551_x_at**	KLHL24	kelch-like 24 (Drosophila)
**235727_at**	KLHL28	kelch-like 28 (Drosophila)
**216699_s_at**	KLK1	kallikrein 1
**205821_at**	KLRK1	killer cell lectin-like receptor subfamily K, member 1
**204009_s_at**	KRAS	v-Ki-ras2 Kirsten rat sarcoma viral oncogene homolog
**233329_s_at**	KRCC1	lysine-rich coiled-coil 1
**201650_at**	KRT19	keratin 19
**213953_at**	KRT20	keratin 20

**209008_x_at**	KRT8	keratin 8
**200914_x_at**	KTN1	kinectin 1 (kinesin receptor)
**229393_at**	L3MBTL3	l(3)mbt-like 3 (Drosophila)
**227048_at**	LAMA1	laminin, alpha 1
**241385_at**	LARP7	La ribonucleoprotein domain family, member 7
**206268_at**	LEFTY1	left-right determination factor 1
**218717_s_at**	LEPREL1	leprecan-like 1
**208450_at**	LGALS2	lectin, galactoside-binding, soluble, 2
**1557197_a_at**	LGALS3	lectin, galactoside-binding, soluble, 3
**204272_at**	LGALS4	lectin, galactoside-binding, soluble, 4
**218326_s_at**	LGR4	leucine-rich repeat-containing G protein-coupled receptor 4
**217892_s_at**	LIMA1	LIM domain and actin binding 1
**202193_at**	LIMK2	LIM domain kinase 2
**1554455_at**	LINS1	lines homolog 1 (Drosophila)
**235871_at**	LIPH	lipase, member H
**212089_at**	LMNA	lamin A/C
**242633_x_at**	LOC100128510	hypothetical protein LOC100128510
**236118_at**	LOC100128893	hypothetical protein LOC100128893
**233527_at**	LOC100129069	Hypothetical protein LOC100129069
**242364_x_at**	LOC100131096	hypothetical LOC100131096
**1558569_at**	LOC100131541	Hypothetical LOC100131541
**211996_s_at**	LOC100132247 /// LOC348162 /// LOC613037 /// LOC728888 /// NPIPL3	similar to Uncharacterized protein KIAA0220 /// hypothetical protein 348162 /// nuclear pore complex interacting protein pseudogene /// similar to acyl-CoA synthetase medium-chain family member 2 /// nuclear pore complex interacting protein-like 3
**227234_at**	LOC100132815	Hypothetical protein LOC100132815
**1559125_at**	LOC100133019	similar to hCG1983765
**214850_at**	LOC100170939	glucuronidase, beta pseudogene
**235167_at**	LOC100190986	hypothetical LOC100190986
**226809_at**	LOC100216479	hypothetical LOC100216479
**235217_at**	LOC100216546	hypothetical LOC100216546
**213089_at**	LOC100272216	hypothetical LOC100272216
**228528_at**	LOC100286909	Hypothetical protein LOC100286909
**230991_at**	LOC100287411	Similar to proline-rich nuclear receptor coactivator 2
**215123_at**	LOC100288332 /// LOC100288583 /// NPIPL3	similar to acyl-CoA synthetase medium-chain family member 2 /// hypothetical protein LOC100288583 /// nuclear pore complex interacting protein-like 3
**236656_s_at**	LOC100288911	hypothetical protein LOC100288911
**1560089_at**	LOC100289019	hypothetical LOC100289019
**232919_at**	LOC100289297	Hypothetical protein LOC100289297
**233775_x_at**	LOC100289333	Hypothetical protein LOC100289333
**232420_x_at**	LOC100289341	similar to hCG2022304
**1556078_at**	LOC100289632	Hypothetical protein LOC100289632
**233960_s_at**	LOC115110	hypothetical protein LOC115110
**230505_at**	LOC145474	hypothetical protein LOC145474
**239594_at**	LOC145837	hypothetical LOC145837
**239965_at**	LOC151878	hypothetical protein LOC151878
**220609_at**	LOC202181	hypothetical protein LOC202181
**230077_at**	LOC220729 /// SDHA /// SDHAP1 /// SDHAP2	succinate dehydrogenase complex, subunit A, flavoprotein pseudogene /// succinate dehydrogenase complex, subunit A, flavoprotein (Fp) /// succinate dehydrogenase complex, subunit A, flavoprotein pseudogene 1 /// succinate dehydrogenase complex, subunit A, flavoprotein pseudogene 2

**230937_at**	LOC285835	hypothetical protein LOC285835
**217506_at**	LOC339290	Hypothetical LOC339290
**232455_x_at**	LOC340085	hypothetical protein LOC340085
**241418_at**	LOC344887	Similar to hCG2041270
**236351_at**	LOC389023	hypothetical gene supported by BC032913; BC048425
**237450_at**	LOC389332	hypothetical LOC389332
**236001_at**	LOC400573	hypothetical gene supported by BC015790; BC041634
**228601_at**	LOC401022	hypothetical LOC401022
**239313_at**	LOC401320	Hypothetical LOC401320
**1557293_at**	LOC440993	hypothetical LOC440993
**240890_at**	LOC643733	hypothetical LOC643733
**242889_x_at**	LOC645431	hypothetical LOC645431
**238715_at**	LOC646014	Hypothetical protein LOC646014
**238143_at**	LOC646627	phospholipase inhibitor
**1568780_at**	LOC649305	hypothetical LOC649305
**239241_at**	LOC727869	hypothetical LOC727869
**1559322_at**	LOC727916	hypothetical protein LOC727916
**233599_at**	LOC728061	hCG2003663
**1560199_x_at**	LOC728153	similar to FAM133B protein
**239319_at**	LOC728342	Hypothetical protein LOC728342
**213737_x_at**	LOC728498	Golgin subfamily A member 8-like protein 1
**1555872_a_at**	LOC728903 /// MGC21881	hypothetical LOC728903 /// hypothetical locus MGC21881
**214291_at**	LOC729046 /// RPL17	similar to ribosomal protein L17 /// ribosomal protein L17
**214375_at**	LOC729222 /// PPFIBP1	similar to PTPRF interacting protein binding protein 1 /// PTPRF interacting protein, binding protein 1 (liprin beta 1)
**218366_x_at**	LOC731602 /// METT11D1	similar to methyltransferase 11 domain containing 1 isoform 2 /// methyltransferase 11 domain containing 1
**213502_x_at**	LOC91316	glucuronidase, beta/immunoglobulin lambda-like polypeptide 1 pseudogene
**221833_at**	LONP2	Lon peptidase 2, peroxisomal
**242931_at**	LONRF3	LON peptidase N-terminal domain and ring finger 3
**204036_at**	LPAR1	lysophosphatidic acid receptor 1
**230252_at**	LPAR5	lysophosphatidic acid receptor 5
**213078_x_at**	LPCAT4	lysophosphatidylcholine acyltransferase 4
**202821_s_at**	LPP	LIM domain containing preferred translocation partner in lipoma
**226908_at**	LRIG3	leucine-rich repeats and immunoglobulin-like domains 3
**204674_at**	LRMP	lymphoid-restricted membrane protein
**35974_at**	LRMP	lymphoid-restricted membrane protein
**220376_at**	LRRC19	leucine rich repeat containing 19
**220622_at**	LRRC31	leucine rich repeat containing 31
**224686_x_at**	LRRC37A2	Leucine rich repeat containing 37, member A2
**211452_x_at**	LRRFIP1	leucine rich repeat (in FLII) interacting protein 1
**220610_s_at**	LRRFIP2	leucine rich repeat (in FLII) interacting protein 2
**224512_s_at**	LSMD1	LSM domain containing 1
**208835_s_at**	LUC7L3	LUC7-like 3 (S. cerevisiae)
**218729_at**	LXN	latexin
**227712_at**	LYRM2	LYR motif containing 2
**210943_s_at**	LYST	lysosomal trafficking regulator
**232168_x_at**	MACF1	microtubule-actin crosslinking factor 1
**226770_at**	MAGI3	membrane associated guanylate kinase, WW and PDZ domain containing 3

**231735_s_at**	MALAT1	metastasis associated lung adenocarcinoma transcript 1 (non-protein coding)
**229473_at**	MAMDC4	MAM domain containing 4
**204388_s_at**	MAOA	monoamine oxidase A
**205698_s_at**	MAP2K6	mitogen-activated protein kinase kinase 6
**219278_at**	MAP3K6	mitogen-activated protein kinase kinase kinase 6
**203553_s_at**	MAP4K5	mitogen-activated protein kinase kinase kinase kinase 5
**213256_at**	MARCH3	membrane-associated ring finger (C3HC4) 3
**201669_s_at**	MARCKS	myristoylated alanine-rich protein kinase C substrate
**235141_at**	MARVELD2	MARVEL domain containing 2
**233634_at**	MARVELD3	MARVEL domain containing 3
**202350_s_at**	MATN2	matrilin 2
**204179_at**	MB	myoglobin
**203353_s_at**	MBD1	methyl-CpG binding domain protein 1
**209579_s_at**	MBD4	methyl-CpG binding domain protein 4
**201152_s_at**	MBNL1	muscleblind-like (Drosophila)
**232138_at**	MBNL2	Muscleblind-like 2 (Drosophila)
**229498_at**	MBNL3	muscleblind-like 3 (Drosophila)
**227379_at**	MBOAT1	membrane bound O-acyltransferase domain containing 1
**229021_at**	MCTP2	multiple C2 domains, transmembrane 2
**211675_s_at**	MDFIC	MyoD family inhibitor domain containing
**235374_at**	MDH1	Malate dehydrogenase 1, NAD (soluble)
**236814_at**	MDM4	Mdm4 p53 binding protein homolog (mouse)
**226420_at**	MECOM	MDS1 and EVI1 complex locus
**226958_s_at**	MED11	mediator complex subunit 11
**207078_at**	MED6	mediator complex subunit 6
**204069_at**	MEIS1	Meis homeobox 1
**214077_x_at**	MEIS3P1	Meis homeobox 3 pseudogene 1
**242111_at**	METTL3	methyltransferase like 3
**209703_x_at**	METTL7A	methyltransferase like 7A
**201155_s_at**	MFN2	mitofusin 2
**218109_s_at**	MFSD1	major facilitator superfamily domain containing 1
**242372_s_at**	MFSD4	major facilitator superfamily domain containing 4
**228040_at**	MGC21881	hypothetical locus MGC21881
**239001_at**	MGST1	Microsomal glutathione S-transferase 1
**204168_at**	MGST2	microsomal glutathione S-transferase 2
**201403_s_at**	MGST3	microsomal glutathione S-transferase 3
**212310_at**	MIA3	melanoma inhibitory activity family, member 3
**219332_at**	MICALL2	MICAL-like 2
**214246_x_at**	MINK1	misshapen-like kinase 1 (zebrafish)
**243256_at**	MKNK1	MAP kinase interacting serine/threonine kinase 1
**223190_s_at**	MLL5	myeloid/lymphoid or mixed-lineage leukemia 5 (trithorax homolog, Drosophila)
**1569652_at**	MLLT3	myeloid/lymphoid or mixed-lineage leukemia (trithorax homolog, Drosophila); translocated to, 3
**218211_s_at**	MLPH	melanophilin
**225157_at**	MLXIP	MLX interacting protein
**239273_s_at**	MMP28	matrix metallopeptidase 28
**219265_at**	MOBKL2B	MOB1, Mps One Binder kinase activator-like 2B (yeast)
**232428_at**	MOGAT2	monoacylglycerol O-acyltransferase 2
**212754_s_at**	MON2	MON2 homolog (S. cerevisiae)
**235864_at**	MPP5	Membrane protein, palmitoylated 5 (MAGUK p55 subfamily member 5)

**213727_x_at**	MPPE1	metallophosphoesterase 1
**210210_at**	MPZL1	myelin protein zero-like 1
**230518_at**	MPZL2	myelin protein zero-like 2
**227226_at**	MRAP2	melanocortin 2 receptor accessory protein 2
**222775_s_at**	MRPL35	mitochondrial ribosomal protein L35
**222499_at**	MRPS16	mitochondrial ribosomal protein S16
**237560_at**	MRPS5	Mitochondrial ribosomal protein S5
**220834_at**	MS4A12	membrane-spanning 4-domains, subfamily A, member 12
**224355_s_at**	MS4A8B	membrane-spanning 4-domains, subfamily A, member 8B
**216320_x_at**	MST1	macrophage stimulating 1 (hepatocyte growth factor-like)
**215563_s_at**	MST1P9	macrophage stimulating 1 (hepatocyte growth factor-like) pseudogene 9
**216336_x_at**	MT1E	metallothionein 1E
**213629_x_at**	MT1F	metallothionein 1F
**204745_x_at**	MT1G	metallothionein 1G
**206461_x_at**	MT1H	metallothionein 1H
**217546_at**	MT1M	metallothionein 1M
**211456_x_at**	MT1P2	metallothionein 1 pseudogene 2
**204326_x_at**	MT1X	metallothionein 1X
**212185_x_at**	MT2A	metallothionein 2A
**36920_at**	MTM1	myotubularin 1
**205076_s_at**	MTMR11	myotubularin related protein 11
**233101_at**	MTMR9	myotubularin related protein 9
**239576_at**	MTUS1	Microtubule associated tumor suppressor 1
**213693_s_at**	MUC1	mucin 1, cell surface associated
**226654_at**	MUC12	mucin 12, cell surface associated
**204673_at**	MUC2	mucin 2, oligomeric mucus/gel-forming
**214898_x_at**	MUC3B	mucin 3B, cell surface associated
**204895_x_at**	MUC4	mucin 4, cell surface associated
**213432_at**	MUC5B	mucin 5B, oligomeric mucus/gel-forming
**218139_s_at**	MUDENG	MU-2/AP1M2 domain containing, death-inducing
**223347_at**	MUM1	melanoma associated antigen (mutated) 1
**202180_s_at**	MVP	major vault protein
**201497_x_at**	MYH11	myosin, heavy chain 11, smooth muscle
**214002_at**	MYL6	myosin, light chain 6, alkali, smooth muscle and non-muscle
**236022_at**	MYO19	myosin XIX
**211916_s_at**	MYO1A	myosin IA
**214656_x_at**	MYO1C	myosin IC
**218966_at**	MYO5C	myosin VC
**242512_at**	MYO9A	myosin IXA
**201798_s_at**	MYOF	myoferlin
**232946_s_at**	NADSYN1	NAD synthetase 1
**218189_s_at**	NANS	N-acetylneuraminic acid synthase
**219368_at**	NAP1L2	nucleosome assembly protein 1-like 2
**238722_x_at**	NAPEPLD	N-acyl phosphatidylethanolamine phospholipase D
**200027_at**	NARS	asparaginyl-tRNA synthetase
**201969_at**	NASP	nuclear autoantigenic sperm protein (histone-binding)
**214440_at**	NAT1	N-acetyltransferase 1 (arylamine N-acetyltransferase)
**1554106_at**	NBEAL1	neurobeachin-like 1
**37005_at**	NBL1	neuroblastoma, suppression of tumorigenicity 1
**230712_at**	NBPF1	neuroblastoma breakpoint family, member 1
**214693_x_at**	NBPF10	neuroblastoma breakpoint family, member 10

**228993_s_at**	NCRNA00081	non-protein coding RNA 81
**225786_at**	NCRNA00201	non-protein coding RNA 201
**234594_at**	NCRNA00203	non-protein coding RNA 203
**1564070_s_at**	NCRNA00213	non-protein coding RNA 213
**209550_at**	NDN	necdin homolog (mouse)
**227739_at**	NDOR1	NADPH dependent diflavin oxidoreductase 1
**206453_s_at**	NDRG2	NDRG family member 2
**209224_s_at**	NDUFA2	NADH dehydrogenase (ubiquinone) 1 alpha subcomplex, 2, 8kDa
**202000_at**	NDUFA6	NADH dehydrogenase (ubiquinone) 1 alpha subcomplex, 6, 14kDa
**206790_s_at**	NDUFB1	NADH dehydrogenase (ubiquinone) 1 beta subcomplex, 1, 7kDa
**201227_s_at**	NDUFB8	NADH dehydrogenase (ubiquinone) 1 beta subcomplex, 8, 19kDa
**232169_x_at**	NDUFS8	NADH dehydrogenase (ubiquinone) Fe-S protein 8, 23kDa (NADH-coenzyme Q reductase)
**224565_at**	NEAT1	nuclear paraspeckle assembly transcript 1 (non-protein coding)
**212448_at**	NEDD4L	neural precursor cell expressed, developmentally down-regulated 4-like
**202149_at**	NEDD9	neural precursor cell expressed, developmentally down-regulated 9
**204321_at**	NEO1	neogenin homolog 1 (chicken)
**222957_at**	NEU4	sialidase 4
**217526_at**	NFATC2IP	nuclear factor of activated T-cells, cytoplasmic, calcineurin-dependent 2 interacting protein
**226895_at**	NFIC	Nuclear factor I/C (CCAAT-binding transcription factor)
**201502_s_at**	NFKBIA	nuclear factor of kappa light polypeptide gene enhancer in B-cells inhibitor, alpha
**202215_s_at**	NFYC	nuclear transcription factor Y, gamma
**226490_at**	NHSL1	NHS-like 1
**232158_x_at**	NIPAL1	NIPA-like domain containing 1
**227001_at**	NIPAL2	NIPA-like domain containing 2
**225876_at**	NIPAL3	NIPA-like domain containing 3
**202891_at**	NIT1	nitrilase 1
**202379_s_at**	NKTR	natural killer-tumor recognition sequence
**206915_at**	NKX2-2	NK2 homeobox 2
**234762_x_at**	NLN	Neurolysin (metallopeptidase M3 family)
**221690_s_at**	NLRP2	NLR family, pyrin domain containing 2
**214722_at**	NOTCH2NL	Notch homolog 2 (Drosophila) N-terminal like
**214321_at**	NOV	nephroblastoma overexpressed gene
**218086_at**	NPDC1	neural proliferation, differentiation and control, 1
**235432_at**	NPHP3	nephronophthisis 3 (adolescent)
**201865_x_at**	NR3C1	nuclear receptor subfamily 3, group C, member 1 (glucocorticoid receptor)
**205259_at**	NR3C2	nuclear receptor subfamily 3, group C, member 2
**208343_s_at**	NR5A2	nuclear receptor subfamily 5, group A, member 2
**210174_at**	NR5A2	nuclear receptor subfamily 5, group A, member 2
**226499_at**	NRARP	NOTCH-regulated ankyrin repeat protein
**229422_at**	NRD1	nardilysin (N-arginine dibasic convertase)
**202600_s_at**	NRIP1	nuclear receptor interacting protein 1
**1566785_x_at**	NSF	N-ethylmaleimide-sensitive factor
**232148_at**	NSMAF	Neutral sphingomyelinase (N-SMase) activation associated factor
**222128_at**	NSUN6	NOP2/Sun domain family, member 6
**238983_at**	NSUN7	NOP2/Sun domain family, member 7
**236703_at**	NT5C2	5'-nucleotidase, cytosolic II
**203939_at**	NT5E	5'-nucleotidase, ecto (CD73)
**223315_at**	NTN4	netrin 4

**203675_at**	NUCB2	nucleobindin 2
**223535_at**	NUDT12	nudix (nucleoside diphosphate linked moiety X)-type motif 12
**228341_at**	NUDT16	nudix (nucleoside diphosphate linked moiety X)-type motif 16
**220183_s_at**	NUDT6	nudix (nucleoside diphosphate linked moiety X)-type motif 6
**218375_at**	NUDT9	nudix (nucleoside diphosphate linked moiety X)-type motif 9
**214250_at**	NUMA1	nuclear mitotic apparatus protein 1
**207545_s_at**	NUMB	numb homolog (Drosophila)
**239748_x_at**	OCIAD1	OCIA domain containing 1
**213131_at**	OLFM1	olfactomedin 1
**206323_x_at**	OPHN1	oligophrenin 1
**236121_at**	OR51E2	olfactory receptor, family 51, subfamily E, member 2
**218556_at**	ORMDL2	ORM1-like 2 (S. cerevisiae)
**209485_s_at**	OSBPL1A	oxysterol binding protein-like 1A
**227946_at**	OSBPL7	oxysterol binding protein-like 7
**230830_at**	OSTBETA	organic solute transporter beta
**206048_at**	OVOL2	ovo-like 2 (Drosophila)
**202780_at**	OXCT1	3-oxoacid CoA transferase 1
**207455_at**	P2RY1	purinergic receptor P2Y, G-protein coupled, 1
**235259_at**	PACRGL	PARK2 co-regulated-like
**201651_s_at**	PACSIN2	protein kinase C and casein kinase substrate in neurons 2
**1554384_at**	PADI2	peptidyl arginine deiminase, type II
**225622_at**	PAG1	phosphoprotein associated with glycosphingolipid microdomains 1
**200907_s_at**	PALLD	palladin, cytoskeletal associated protein
**221751_at**	PANK3	pantothenate kinase 3
**238706_at**	PAPD4	PAP associated domain containing 4
**203058_s_at**	PAPSS2	3'-phosphoadenosine 5'-phosphosulfate synthase 2
**242871_at**	PAQR5	progestin and adipoQ receptor family member V
**204687_at**	PARM1	prostate androgen-regulated mucin-like protein 1
**229515_at**	PAWR	PRKC, apoptosis, WT1, regulator
**219543_at**	PBLD	phenazine biosynthesis-like protein domain containing
**232613_at**	PBRM1	polybromo 1
**213517_at**	PCBP2	poly(rC) binding protein 2
**208383_s_at**	PCK1	phosphoenolpyruvate carboxykinase 1 (soluble)
**213558_at**	PCLO	piccolo (presynaptic cytomatrix protein)
**225069_at**	PCYT1A	Phosphate cytidylyltransferase 1, choline, alpha
**205559_s_at**	PCSK5	proprotein convertase subtilisin/kexin type 5
**203118_at**	PCSK7	proprotein convertase subtilisin/kexin type 7
**212594_at**	PDCD4	programmed cell death 4 (neoplastic transformation inhibitor)
**222380_s_at**	PDCD6	Programmed cell death 6
**206792_x_at**	PDE4C	phosphodiesterase 4C, cAMP-specific (phosphodiesterase E1 dunce homolog, Drosophila)
**228962_at**	PDE4D	phosphodiesterase 4D, cAMP-specific (phosphodiesterase E3 dunce homolog, Drosophila)
**214129_at**	PDE4DIP	phosphodiesterase 4D interacting protein
**205593_s_at**	PDE9A	phosphodiesterase 9A
**218718_at**	PDGFC	platelet derived growth factor C
**203131_at**	PDGFRA	platelet-derived growth factor receptor, alpha polypeptide
**229453_at**	PDIA3	protein disulfide isomerase family A, member 3
**208658_at**	PDIA4	protein disulfide isomerase family A, member 4
**212053_at**	PDXDC1	pyridoxal-dependent decarboxylase domain containing 1
**232288_at**	PDXDC1 /// PDXDC2	pyridoxal-dependent decarboxylase domain containing 1 /// pyridoxal-dependent decarboxylase domain containing 2

**218025_s_at**	PECI	peroxisomal D3,D2-enoyl-CoA isomerase
**223619_x_at**	PECR	peroxisomal trans-2-enoyl-CoA reductase
**232304_at**	PELI1	Pellino homolog 1 (Drosophila)
**220576_at**	PGAP1	post-GPI attachment to proteins 1
**55616_at**	PGAP3	post-GPI attachment to proteins 3
**215179_x_at**	PGF	Placental growth factor
**235615_at**	PGGT1B	protein geranylgeranyltransferase type I, beta subunit
**201968_s_at**	PGM1	phosphoglucomutase 1
**213227_at**	PGRMC2	progesterone receptor membrane component 2
**221611_s_at**	PHF7	PHD finger protein 7
**242248_at**	PHKB	phosphorylase kinase, beta
**212134_at**	PHLDB1	pleckstrin homology-like domain, family B, member 1
**225688_s_at**	PHLDB2	pleckstrin homology-like domain, family B, member 2
**212719_at**	PHLPP1	PH domain and leucine rich repeat protein phosphatase 1
**225683_x_at**	PHPT1	phosphohistidine phosphatase 1
**219093_at**	PID1	phosphotyrosine interaction domain containing 1
**219048_at**	PIGN	phosphatidylinositol glycan anchor biosynthesis, class N
**232101_s_at**	PIGN	phosphatidylinositol glycan anchor biosynthesis, class N
**226147_s_at**	PIGR	polymeric immunoglobulin receptor
**241905_at**	PIK3C2A	Phosphoinositide-3-kinase, class 2, alpha polypeptide
**217620_s_at**	PIK3CB	phosphoinositide-3-kinase, catalytic, beta polypeptide
**225321_s_at**	PILRB	paired immunoglobin-like type 2 receptor beta
**209019_s_at**	PINK1	PTEN induced putative kinase 1
**230076_at**	PITPNM3	PITPNM family member 3
**201133_s_at**	PJA2	praja ring finger 2
**223551_at**	PKIB	protein kinase (cAMP-dependent, catalytic) inhibitor beta
**207717_s_at**	PKP2	plakophilin 2
**207222_at**	PLA2G10	phospholipase A2, group X
**203649_s_at**	PLA2G2A	phospholipase A2, group IIA (platelets, synovial fluid)
**210145_at**	PLA2G4A	phospholipase A2, group IVA (cytosolic, calcium-dependent)
**219014_at**	PLAC8	placenta-specific 8
**201860_s_at**	PLAT	plasminogen activator, tissue
**205111_s_at**	PLCE1	phospholipase C, epsilon 1
**204613_at**	PLCG2	phospholipase C, gamma 2 (phosphatidylinositol-specific)
**213309_at**	PLCL2	phospholipase C-like 2
**226636_at**	PLD1	phospholipase D1, phosphatidylcholine-specific
**229245_at**	PLEKHA6	pleckstrin homology domain containing, family A member 6
**225726_s_at**	PLEKHH1	pleckstrin homology domain containing, family H (with MyTH4 domain) member 1
**204519_s_at**	PLLP	plasma membrane proteolipid (plasmolipin)
**213241_at**	PLXNC1	plexin C1
**225298_at**	PNKD	paroxysmal nonkinesigenic dyskinesia
**211766_s_at**	PNLIPRP2	pancreatic lipase-related protein 2
**207109_at**	POU2F3	POU class 2 homeobox 3
**1559496_at**	PPA2	pyrophosphatase (inorganic) 2
**210946_at**	PPAP2A	phosphatidic acid phosphatase type 2A
**219195_at**	PPARGC1A	peroxisome proliferator-activated receptor gamma, coactivator 1 alpha
**1553639_a_at**	PPARGC1B	peroxisome proliferator-activated receptor gamma, coactivator 1 beta
**204517_at**	PPIC	peptidylprolyl isomerase C (cyclophilin C)
**228469_at**	PPID	Peptidylprolyl isomerase D
**223999_at**	PPIL2	peptidylprolyl isomerase (cyclophilin)-like 2
**204578_at**	PPIP5K1	diphosphoinositol pentakisphosphate kinase 1

**1557553_at**	PPP1R12B	protein phosphatase 1, regulatory (inhibitor) subunit 12B
**216347_s_at**	PPP1R13B	protein phosphatase 1, regulatory (inhibitor) subunit 13B
**227409_at**	PPP1R3E	protein phosphatase 1, regulatory (inhibitor) subunit 3E
**221088_s_at**	PPP1R9A	protein phosphatase 1, regulatory (inhibitor) subunit 9A
**236492_at**	PPP2R2A	protein phosphatase 2, regulatory subunit B, alpha
**209632_at**	PPP2R3A	protein phosphatase 2 (formerly 2A), regulatory subunit B'', alpha
**203338_at**	PPP2R5E	protein phosphatase 2, regulatory subunit B', epsilon isoform
**228070_at**	PPP2R5E	protein phosphatase 2, regulatory subunit B', epsilon isoform
**202425_x_at**	PPP3CA	protein phosphatase 3, catalytic subunit, alpha isozyme
**202432_at**	PPP3CB	protein phosphatase 3, catalytic subunit, beta isozyme
**201594_s_at**	PPP4R1	protein phosphatase 4, regulatory subunit 1
**218208_at**	PQLC1	PQ loop repeat containing 1
**215067_x_at**	PRDX2	peroxiredoxin 2
**200844_s_at**	PRDX6	peroxiredoxin 6
**237291_at**	PRDXDD1P	PrdX deacylase domain containing 1, pseudogene
**227892_at**	PRKAA2	protein kinase, AMP-activated, alpha 2 catalytic subunit
**202742_s_at**	PRKACB	protein kinase, cAMP-dependent, catalytic, beta
**204842_x_at**	PRKAR2A	protein kinase, cAMP-dependent, regulatory, type II, alpha
**203680_at**	PRKAR2B	protein kinase, cAMP-dependent, regulatory, type II, beta
**1552797_s_at**	PROM2	prominin 2
**230270_at**	PRPF38B	PRP38 pre-mRNA processing factor 38 (yeast) domain containing B
**202127_at**	PRPF4B	PRP4 pre-mRNA processing factor 4 homolog B (yeast)
**232215_x_at**	PRR11	proline rich 11
**207291_at**	PRRG4	proline rich Gla (G-carboxyglutamic acid) 4 (transmembrane)
**242055_at**	PSMG4	Proteasome (prosome, macropain) assembly chaperone 4
**226110_at**	PTAR1	protein prenyltransferase alpha subunit repeat containing 1
**222796_at**	PTCD1	pentatricopeptide repeat domain 1
**225363_at**	PTEN	phosphatase and tensin homolog
**215894_at**	PTGDR	prostaglandin D2 receptor (DP)
**204897_at**	PTGER4	prostaglandin E receptor 4 (subtype EP4)
**205128_x_at**	PTGS1	prostaglandin-endoperoxide synthase 1 (prostaglandin G/H synthase and cyclooxygenase)
**200733_s_at**	PTP4A1	protein tyrosine phosphatase type IVA, member 1
**226380_at**	PTPN21	protein tyrosine phosphatase, non-receptor type 21
**212587_s_at**	PTPRC	protein tyrosine phosphatase, receptor type, C
**200636_s_at**	PTPRF	protein tyrosine phosphatase, receptor type, F
**208300_at**	PTPRH	protein tyrosine phosphatase, receptor type, H
**1555579_s_at**	PTPRM	protein tyrosine phosphatase, receptor type, M
**213325_at**	PVRL3	poliovirus receptor-related 3
**227999_at**	PWWP2B	PWWP domain containing 2B
**219076_s_at**	PXMP2	peroxisomal membrane protein 2, 22kDa
**211253_x_at**	PYY	peptide YY
**230523_at**	QSOX1	quiescin Q6 sulfhydryl oxidase 1
**202252_at**	RAB13	RAB13, member RAS oncogene family
**243496_at**	RAB18	RAB18, member RAS oncogene family
**219562_at**	RAB26	RAB26, member RAS oncogene family
**210951_x_at**	RAB27A	RAB27A, member RAS oncogene family
**228708_at**	RAB27B	RAB27B, member RAS oncogene family
**217597_x_at**	RAB40B	RAB40B, member RAS oncogene family
**219125_s_at**	RAG1AP1	recombination activating gene 1 activating protein 1
**226957_x_at**	RALBP1	ralA binding protein 1
**213049_at**	RALGAPA1	Ral GTPase activating protein, alpha subunit 1 (catalytic)

**232500_at**	RALGAPA2	Ral GTPase activating protein, alpha subunit 2 (catalytic)
**242712_x_at**	RANBP2 /// RGPD1 /// RGPD2 /// RGPD3 /// RGPD4 /// RGPD5 /// RGPD6 /// RGPD8	RAN binding protein 2 /// RANBP2-like and GRIP domain containing 1 /// RANBP2-like and GRIP domain containing 2 /// RANBP2-like and GRIP domain containing 3 /// RANBP2-like and GRIP domain containing 4 /// RANBP2-like and GRIP domain containing 5 /// RANBP2-like and GRIP domain containing 6 /// RANBP2-like and GRIP domain containing 8
**202362_at**	RAP1A	RAP1A, member of RAS oncogene family
**203911_at**	RAP1GAP	RAP1 GTPase activating protein
**218657_at**	RAPGEFL1	Rap guanine nucleotide exchange factor (GEF)-like 1
**1552482_at**	RAPH1	Ras association (RalGDS/AF-6) and pleckstrin homology domains 1
**206391_at**	RARRES1	retinoic acid receptor responder (tazarotene induced) 1
**209496_at**	RARRES2	retinoic acid receptor responder (tazarotene induced) 2
**223467_at**	RASD1	RAS, dexamethasone-induced 1
**1553185_at**	RASEF	RAS and EF-hand domain containing
**229147_at**	RASSF6	Ras association (RalGDS/AF-6) domain family member 6
**40359_at**	RASSF7	Ras association (RalGDS/AF-6) domain family (N-terminal) member 7
**239071_at**	RBBP4	Retinoblastoma binding protein 4
**228455_at**	RBM15	RNA binding motif protein 15
**235004_at**	RBM24	RNA binding motif protein 24
**236613_at**	RBM25	RNA binding motif protein 25
**213718_at**	RBM4	RNA binding motif protein 4
**222496_s_at**	RBM47	RNA binding motif protein 47
**236291_at**	RDH5	retinol dehydrogenase 5 (11-cis/9-cis)
**223447_at**	REG4	regenerating islet-derived family, member 4
**202297_s_at**	RER1	RER1 retention in endoplasmic reticulum 1 homolog (S. cerevisiae)
**223970_at**	RETNLB	resistin like beta
**218124_at**	RETSAT	retinol saturase (all-trans-retinol 13,14-reductase)
**238736_at**	REV3L	REV3-like, catalytic subunit of DNA polymerase zeta (yeast)
**1552673_at**	RFX6	regulatory factor X, 6
**203169_at**	RGP1	RGP1 retrograde golgi transport homolog (S. cerevisiae)
**210258_at**	RGS13	regulator of G-protein signaling 13
**202388_at**	RGS2	regulator of G-protein signaling 2, 24kDa
**1554897_s_at**	RHBDL2	rhomboid, veinlet-like 2 (Drosophila)
**205414_s_at**	RICH2	Rho-type GTPase-activating protein RICH2
**215588_x_at**	RIOK3	RIO kinase 3 (yeast)
**201785_at**	RNASE1	ribonuclease, RNase A family, 1 (pancreatic)
**213397_x_at**	RNASE4	ribonuclease, RNase A family, 4
**229285_at**	RNASEL	ribonuclease L (2',5'-oligoisoadenylate synthetase-dependent)
**207735_at**	RNF125	ring finger protein 125
**226077_at**	RNF145	ring finger protein 145
**233819_s_at**	RNF160	ring finger protein 160
**219739_at**	RNF186	ring finger protein 186
**236288_at**	RNF34	ring finger protein 34
**226999_at**	RNPC3	RNA-binding region (RNP1, RRM) containing 3
**205805_s_at**	ROR1	receptor tyrosine kinase-like orphan receptor 1
**228806_at**	RORC	RAR-related orphan receptor C
**221593_s_at**	RPL31	ribosomal protein L31
**215208_x_at**	RPL35A	Ribosomal protein L35a
**207585_s_at**	RPL36AL	ribosomal protein L36a-like
**224766_at**	RPL37	Ribosomal protein L37
**214041_x_at**	RPL37A	Ribosomal protein L37a
**1555878_at**	RPS24	Ribosomal protein S24

**218007_s_at**	RPS27L	ribosomal protein S27-like
**203379_at**	RPS6KA1	ribosomal protein S6 kinase, 90kDa, polypeptide 1
**215032_at**	RREB1	ras responsive element binding protein 1
**238412_at**	RRN3P3	RNA polymerase I transcription factor homolog (S. cerevisiae) pseudogene 3
**230093_at**	RSPH1	radial spoke head 1 homolog (Chlamydomonas)
**219957_at**	RUFY2	RUN and FYVE domain containing 2
**218377_s_at**	RWDD2B	RWD domain containing 2B
**200872_at**	S100A10	S100 calcium binding protein A10
**202598_at**	S100A13	S100 calcium binding protein A13
**218677_at**	S100A14	S100 calcium binding protein A14
**227998_at**	S100A16	S100 calcium binding protein A16
**229402_at**	SAMD13	sterile alpha motif domain containing 13
**1570210_x_at**	SAPS2	SAPS domain family, member 2
**215591_at**	SATB2	SATB homeobox 2
**233914_s_at**	SBF2	SET binding factor 2
**240830_at**	SCARNA17	small Cajal body-specific RNA 17
**233229_at**	SCFD1	sec1 family domain containing 1
**204035_at**	SCG2	secretogranin II
**203889_at**	SCG5	secretogranin V (7B2 protein)
**205979_at**	SCGB2A1	secretoglobin, family 2A, member 1
**1552365_at**	SCIN	scinderin
**206950_at**	SCN9A	sodium channel, voltage-gated, type IX, alpha subunit
**203453_at**	SCNN1A	sodium channel, nonvoltage-gated 1 alpha
**205464_at**	SCNN1B	sodium channel, nonvoltage-gated 1, beta
**201339_s_at**	SCP2	sterol carrier protein 2
**211733_x_at**	SCP2	sterol carrier protein 2
**224960_at**	SCYL2	SCY1-like 2 (S. cerevisiae)
**233565_s_at**	SDCBP2	syndecan binding protein (syntenin) 2
**1569594_a_at**	SDCCAG1	serologically defined colon cancer antigen 1
**201093_x_at**	SDHA	succinate dehydrogenase complex, subunit A, flavoprotein (Fp)
**222021_x_at**	SDHAP1	succinate dehydrogenase complex, subunit A, flavoprotein pseudogene 1
**226693_at**	SDHAP2	succinate dehydrogenase complex, subunit A, flavoprotein pseudogene 2
**228274_at**	SDSL	serine dehydratase-like
**202375_at**	SEC24D	SEC24 family, member D (S. cerevisiae)
**208942_s_at**	SEC62	SEC62 homolog (S. cerevisiae)
**202061_s_at**	SEL1L	sel-1 suppressor of lin-12-like (C. elegans)
**214433_s_at**	SELENBP1	selenium binding protein 1
**219194_at**	SEMA4G	sema domain, immunoglobulin domain (Ig), transmembrane domain (TM) and short cytoplasmic domain, (semaphorin) 4G
**215028_at**	SEMA6A	sema domain, transmembrane domain (TM), and cytoplasmic domain, (semaphorin) 6A
**230071_at**	SEPT11	septin 11
**1559038_at**	SEPT2	septin 2
**224625_x_at**	SERF2	small EDRK-rich factor 2
**202833_s_at**	SERPINA1	serpin peptidase inhibitor, clade A (alpha-1 antiproteinase, antitrypsin), member 1
**239213_at**	SERPINB1	serpin peptidase inhibitor, clade B (ovalbumin), member 1
**229674_at**	SERTAD4	SERTA domain containing 4
**223196_s_at**	SESN2	sestrin 2
**227478_at**	SETBP1	SET binding protein 1

**212465_at**	SETD3	SET domain containing 3
**33323_r_at**	SFN	stratifin
**221768_at**	SFPQ	Splicing factor proline/glutamine-rich (polypyrimidine tract binding protein associated)
**235611_at**	SFRS12	splicing factor, arginine/serine-rich 12
**222310_at**	SFRS15	splicing factor, arginine/serine-rich 15
**230375_at**	SFRS18	splicing factor, arginine/serine-rich 18
**228760_at**	SFRS2B	splicing factor, arginine/serine-rich 2B
**232597_x_at**	SFRS2IP	splicing factor, arginine/serine-rich 2, interacting protein
**242837_at**	SFRS4	Splicing factor, arginine/serine-rich 4
**203380_x_at**	SFRS5	splicing factor, arginine/serine-rich 5
**213649_at**	SFRS7	splicing factor, arginine/serine-rich 7, 35kDa
**218835_at**	SFTPA2	surfactant protein A2
**213936_x_at**	SFTPB	surfactant protein B
**225913_at**	SGK269	NKF3 kinase family member
**223391_at**	SGPP1	sphingosine-1-phosphate phosphatase 1
**244780_at**	SGPP2	sphingosine-1-phosphate phosphotase 2
**230287_at**	SGSM1	small G protein signaling modulator 1
**214779_s_at**	SGSM3	small G protein signaling modulator 3
**225354_s_at**	SH3BGRL2	SH3 domain binding glutamic acid-rich protein like 2
**221269_s_at**	SH3BGRL3	SH3 domain binding glutamic acid-rich protein like 3
**225589_at**	SH3RF1	SH3 domain containing ring finger 1
**243582_at**	SH3RF2	SH3 domain containing ring finger 2
**228400_at**	SHROOM3	shroom family member 3
**206664_at**	SI	sucrase-isomaltase (alpha-glucosidase)
**224391_s_at**	SIAE	sialic acid acetylesterase
**219734_at**	SIDT1	SID1 transmembrane family, member 1
**218765_at**	SIDT2	SID1 transmembrane family, member 2
**1563455_at**	SIK3	SIK family kinase 3
**233587_s_at**	SIPA1L2	signal-induced proliferation-associated 1 like 2
**213600_at**	SIPA1L3	signal-induced proliferation-associated 1 like 3
**235143_at**	SLC10A7	solute carrier family 10 (sodium/bile acid cotransporter family), member 7
**239805_at**	SLC13A2	solute carrier family 13 (sodium-dependent dicarboxylate transporter), member 2
**207057_at**	SLC16A7	solute carrier family 16, member 7 (monocarboxylic acid transporter 2)
**227506_at**	SLC16A9	solute carrier family 16, member 9 (monocarboxylic acid transporter 9)
**207074_s_at**	SLC18A1	solute carrier family 18 (vesicular monoamine), member 1
**204981_at**	SLC22A18	solute carrier family 22, member 18
**206097_at**	SLC22A18AS	solute carrier family 22 (organic cation transporter), member 18 antisense
**223194_s_at**	SLC22A23	solute carrier family 22, member 23
**242578_x_at**	SLC22A3	Solute carrier family 22 (extraneuronal monoamine transporter), member 3
**209003_at**	SLC25A11	solute carrier family 25 (mitochondrial carrier; oxoglutarate carrier), member 11
**210686_x_at**	SLC25A16	solute carrier family 25 (mitochondrial carrier; Graves disease autoantigen), member 16
**203658_at**	SLC25A20	solute carrier family 25 (carnitine/acylcarnitine translocase), member 20
**225306_s_at**	SLC25A29	solute carrier family 25, member 29
**222529_at**	SLC25A37	solute carrier family 25, member 37
**205097_at**	SLC26A2	solute carrier family 26 (sulfate transporter), member 2
**222217_s_at**	SLC27A3	solute carrier family 27 (fatty acid transporter), member 3

**207249_s_at**	SLC28A2	solute carrier family 28 (sodium-coupled nucleoside transporter), member 2
**221024_s_at**	SLC2A10	solute carrier family 2 (facilitated glucose transporter), member 10
**227176_at**	SLC2A13	solute carrier family 2 (facilitated glucose transporter), member 13
**203306_s_at**	SLC35A1	solute carrier family 35 (CMP-sialic acid transporter), member A1
**209865_at**	SLC35A3	solute carrier family 35 (UDP-N-acetylglucosamine (UDP-GlcNAc) transporter), member A3
**231437_at**	SLC35D2	solute carrier family 35, member D2
**220796_x_at**	SLC35E1	solute carrier family 35, member E1
**213119_at**	SLC36A1	solute carrier family 36 (proton/amino acid symporter), member 1
**234978_at**	SLC36A4	solute carrier family 36 (proton/amino acid symporter), member 4
**218928_s_at**	SLC37A1	solute carrier family 37 (glycerol-3-phosphate transporter), member 1
**202667_s_at**	SLC39A7	solute carrier family 39 (zinc transporter), member 7
**223798_at**	SLC41A2	solute carrier family 41, member 2
**228486_at**	SLC44A1	solute carrier family 44, member 1
**205597_at**	SLC44A4	solute carrier family 44, member 4
**1554027_a_at**	SLC4A4	solute carrier family 4, sodium bicarbonate cotransporter, member 4
**209453_at**	SLC9A1	solute carrier family 9 (sodium/hydrogen exchanger), member 1
**211116_at**	SLC9A2	solute carrier family 9 (sodium/hydrogen exchanger), member 2
**235976_at**	SLITRK6	SLIT and NTRK-like family, member 6
**203021_at**	SLPI	secretory leukocyte peptidase inhibitor
**226563_at**	SMAD2	SMAD family member 2
**1565703_at**	SMAD4	SMAD family member 4
**227719_at**	SMAD9	SMAD family member 9
**212927_at**	SMC5	structural maintenance of chromosomes 5
**1558747_at**	SMCHD1	structural maintenance of chromosomes flexible hinge domain containing 1
**220368_s_at**	SMEK1	SMEK homolog 1, suppressor of mek1 (Dictyostelium)
**213624_at**	SMPDL3A	sphingomyelin phosphodiesterase, acid-like 3A
**223773_s_at**	SNHG12	small nucleolar RNA host gene 12 (non-protein coding)
**244669_at**	SNHG5 /// SNORD50A /// SNORD50B	small nucleolar RNA host gene 5 (non-protein coding) /// small nucleolar RNA, C/D box 50A /// small nucleolar RNA, C/D box 50B
**228879_at**	SNORD104	small nucleolar RNA, C/D box 104
**242146_at**	SNRPA1	Small nuclear ribonucleoprotein polypeptide A'
**222716_s_at**	SNX24	sorting nexin 24
**228479_at**	SOAT1	sterol O-acyltransferase 1
**203373_at**	SOCS2	suppressor of cytokine signaling 2
**226465_s_at**	SON	SON DNA binding protein
**213456_at**	SOSTDC1	sclerostin domain containing 1
**219109_at**	SPAG16	sperm associated antigen 16
**229331_at**	SPATA18	spermatogenesis associated 18 homolog (rat)
**220192_x_at**	SPDEF	SAM pointed domain containing ets transcription factor
**230885_at**	SPG7	spastic paraplegia 7 (pure and complicated autosomal recessive)
**232739_at**	SPIB	Spi-B transcription factor (Spi-1/PU.1 related)
**207214_at**	SPINK4	serine peptidase inhibitor, Kazal type 4
**205185_at**	SPINK5	serine peptidase inhibitor, Kazal type 5
**213994_s_at**	SPON1	spondin 1, extracellular matrix protein
**226353_at**	SPPL2A	signal peptide peptidase-like 2A
**225095_at**	SPTLC2	Serine palmitoyltransferase, long chain base subunit 2
**217995_at**	SQRDL	sulfide quinone reductase-like (yeast)
**242748_at**	SREBF2	sterol regulatory element binding transcription factor 2
**227484_at**	SRGAP1	SLIT-ROBO Rho GTPase activating protein 1

**208610_s_at**	SRRM2	serine/arginine repetitive matrix 2
**203787_at**	SSBP2	single-stranded DNA binding protein 2
**213921_at**	SST	somatostatin
**235591_at**	SSTR1	somatostatin receptor 1
**226229_s_at**	SSU72	SSU72 RNA polymerase II CTD phosphatase homolog (S. cerevisiae)
**203759_at**	ST3GAL4	ST3 beta-galactoside alpha-2,3-sialyltransferase 4
**228821_at**	ST6GAL2	ST6 beta-galactosamide alpha-2,6-sialyltranferase 2
**227725_at**	ST6GALNAC1	ST6 (alpha-N-acetyl-neuraminyl-2,3-beta-galactosyl-1,3)-N-acetylgalactosaminide alpha-2,6-sialyltransferase 1
**222571_at**	ST6GALNAC6	ST6 (alpha-N-acetyl-neuraminyl-2,3-beta-galactosyl-1,3)-N-acetylgalactosaminide alpha-2,6-sialyltransferase 6
**221610_s_at**	STAP2	signal transducing adaptor family member 2
**223103_at**	STARD10	StAR-related lipid transfer (START) domain containing 10
**209478_at**	STRA13	stimulated by retinoic acid 13 homolog (mouse)
**229513_at**	STRBP	spermatid perinuclear RNA binding protein
**238303_at**	STT3B	STT3, subunit of the oligosaccharyltransferase complex, homolog B (S. cerevisiae)
**212111_at**	STX12	syntaxin 12
**226794_at**	STXBP5	syntaxin binding protein 5 (tomosyn)
**220030_at**	STYK1	serine/threonine/tyrosine kinase 1
**221696_s_at**	STYK1	serine/threonine/tyrosine kinase 1
**215772_x_at**	SUCLG2	succinate-CoA ligase, GDP-forming, beta subunit
**224974_at**	SUDS3	suppressor of defective silencing 3 homolog (S. cerevisiae)
**215299_x_at**	SULT1A1	sulfotransferase family, cytosolic, 1A, phenol-preferring, member 1
**211385_x_at**	SULT1A2	sulfotransferase family, cytosolic, 1A, phenol-preferring, member 2
**210580_x_at**	SULT1A3 /// SULT1A4	sulfotransferase family, cytosolic, 1A, phenol-preferring, member 3 /// sulfotransferase family, cytosolic, 1A, phenol-preferring, member 4
**207601_at**	SULT1B1	sulfotransferase family, cytosolic, 1B, member 1
**226850_at**	SUMF1	sulfatase modifying factor 1
**217704_x_at**	SUZ12P	Suppressor of zeste 12 homolog pseudogene
**202565_s_at**	SVIL	supervillin
**1554595_at**	SYMPK	symplekin
**221276_s_at**	SYNC	syncoilin, intermediate filament protein
**202761_s_at**	SYNE2	spectrin repeat containing, nuclear envelope 2
**219156_at**	SYNJ2BP	synaptojanin 2 binding protein
**227274_at**	SYNJ2BP	synaptojanin 2 binding protein
**226086_at**	SYT13	synaptotagmin XIII
**205613_at**	SYT17	synaptotagmin XVII
**225496_s_at**	SYTL2	synaptotagmin-like 2
**227703_s_at**	SYTL4	synaptotagmin-like 4
**242093_at**	SYTL5	synaptotagmin-like 5
**236248_x_at**	TADA2B	transcriptional adaptor 2B
**203938_s_at**	TAF1C	TATA box binding protein (TBP)-associated factor, RNA polymerase I, C, 110kDa
**218750_at**	TAF1D	TATA box binding protein (TBP)-associated factor, RNA polymerase I, D, 41kDa
**226735_at**	TAPT1	transmembrane anterior posterior transformation 1
**226318_at**	TBRG1	transforming growth factor beta regulator 1
**242701_at**	TBRG1	transforming growth factor beta regulator 1
**234970_at**	TC2N	tandem C2 domains, nuclear
**226388_at**	TCEA3	transcription elongation factor A (SII), 3
**227279_at**	TCEAL3	transcription elongation factor A (SII)-like 3
**213311_s_at**	TCF25	transcription factor 25 (basic helix-loop-helix)

**212761_at**	TCF7L2	transcription factor 7-like 2 (T-cell specific, HMG-box)
**203742_s_at**	TDG	thymine-DNA glycosylase
**227580_s_at**	TECPR1	tectonin beta-propeller repeat containing 1
**228670_at**	TEP1	telomerase-associated protein 1
**227624_at**	TET2	tet oncogene family member 2
**229341_at**	TFCP2L1	transcription factor CP2-like 1
**204623_at**	TFF3	trefoil factor 3 (intestinal)
**235086_at**	THBS1	thrombospondin 1
**228716_at**	THRB	thyroid hormone receptor, beta (erythroblastic leukemia viral (v-erb-a) oncogene homolog 2, avian)
**222835_at**	THSD4	thrombospondin, type I, domain containing 4
**242377_x_at**	THUMPD3	THUMP domain containing 3
**201449_at**	TIA1	TIA1 cytotoxic granule-associated RNA binding protein
**218357_s_at**	TIMM8B	translocase of inner mitochondrial membrane 8 homolog B (yeast)
**214773_x_at**	TIPRL	TIP41, TOR signaling pathway regulator-like (S. cerevisiae)
**35148_at**	TJP3	tight junction protein 3 (zona occludens 3)
**1557275_a_at**	TLCD2	TLC domain containing 2
**203222_s_at**	TLE1	transducin-like enhancer of split 1 (E(sp1) homolog, Drosophila)
**212703_at**	TLN2	talin 2
**217758_s_at**	TM9SF3	transmembrane 9 superfamily member 3
**219206_x_at**	TMBIM4	transmembrane BAX inhibitor motif containing 4
**235146_at**	TMCC3	transmembrane and coiled-coil domain family 3
**238886_at**	TMED10	transmembrane emp24-like trafficking protein 10 (yeast)
**205812_s_at**	TMED9	transmembrane emp24 protein transport domain containing 9
**224321_at**	TMEFF2	transmembrane protein with EGF-like and two follistatin-like domains 2
**1552302_at**	TMEM106A	transmembrane protein 106A
**239831_at**	TMEM106C	Transmembrane protein 106C
**228775_at**	TMEM111	transmembrane protein 111
**225822_at**	TMEM125	transmembrane protein 125
**225588_s_at**	TMEM129	transmembrane protein 129
**225568_at**	TMEM141	transmembrane protein 141
**238783_at**	TMEM161B	transmembrane protein 161B
**218962_s_at**	TMEM168	transmembrane protein 168
**240770_at**	TMEM171	transmembrane protein 171
**229693_at**	TMEM220	transmembrane protein 220
**213285_at**	TMEM30B	transmembrane protein 30B
**212622_at**	TMEM41B	transmembrane protein 41B
**228054_at**	TMEM44	transmembrane protein 44
**230323_s_at**	TMEM45B	transmembrane protein 45B
**225182_at**	TMEM50B	transmembrane protein 50B
**225536_at**	TMEM54	transmembrane protein 54
**200620_at**	TMEM59	transmembrane protein 59
**1558323_at**	TMEM72	transmembrane protein 72
**225412_at**	TMEM87B	transmembrane protein 87B
**218065_s_at**	TMEM9B	TMEM9 domain family, member B
**226553_at**	TMPRSS2	transmembrane protease, serine 2
**217733_s_at**	TMSB10	thymosin beta 10
**201175_at**	TMX2	thioredoxin-related transmembrane protein 2
**227345_at**	TNFRSF10D	tumor necrosis factor receptor superfamily, member 10d, decoy with truncated death domain
**207037_at**	TNFRSF11A	tumor necrosis factor receptor superfamily, member 11a, NFKB activator

**202687_s_at**	TNFSF10	tumor necrosis factor (ligand) superfamily, member 10
**209499_x_at**	TNFSF12-TNFSF13 /// TNFSF13	TNFSF12-TNFSF13 readthrough /// tumor necrosis factor (ligand) superfamily, member 13
**210314_x_at**	TNFSF13	tumor necrosis factor (ligand) superfamily, member 13
**213107_at**	TNIK	TRAF2 and NCK interacting kinase
**1558142_at**	TNRC6B	trinucleotide repeat containing 6B
**226059_at**	TOMM40L	translocase of outer mitochondrial membrane 40 homolog (yeast)-like
**204529_s_at**	TOX	thymocyte selection-associated high mobility group box
**232097_at**	TOX4	TOX high mobility group box family member 4
**203421_at**	TP53I11	tumor protein p53 inducible protein 11
**210609_s_at**	TP53I3	tumor protein p53 inducible protein 3
**224836_at**	TP53INP2	tumor protein p53 inducible nuclear protein 2
**203476_at**	TPBG	trophoblast glycoprotein
**201689_s_at**	TPD52	tumor protein D52
**214601_at**	TPH1	tryptophan hydroxylase 1
**238688_at**	TPM1	Tropomyosin 1 (alpha)
**229574_at**	TRA2A	transformer 2 alpha homolog (Drosophila)
**215411_s_at**	TRAF3IP2	TRAF3 interacting protein 2
**202124_s_at**	TRAK2	trafficking protein, kinesin binding 2
**215269_at**	TRAPPC10	trafficking protein particle complex 10
**219736_at**	TRIM36	tripartite motif-containing 36
**203567_s_at**	TRIM38	tripartite motif-containing 38
**231403_at**	TRIO	Triple functional domain (PTPRF interacting)
**219360_s_at**	TRPM4	transient receptor potential cation channel, subfamily M, member 4
**224412_s_at**	TRPM6	transient receptor potential cation channel, subfamily M, member 6
**209114_at**	TSPAN1	tetraspanin 1
**217979_at**	TSPAN13	tetraspanin 13
**202242_at**	TSPAN7	tetraspanin 7
**203824_at**	TSPAN8	tetraspanin 8
**218155_x_at**	TSR1	TSR1, 20S rRNA accumulation, homolog (S. cerevisiae)
**209605_at**	TST	thiosulfate sulfurtransferase (rhodanese)
**219481_at**	TTC13	tetratricopeptide repeat domain 13
**225178_at**	TTC14	tetratricopeptide repeat domain 14
**235651_at**	TTC22	tetratricopeptide repeat domain 22
**210652_s_at**	TTC39A	tetratricopeptide repeat domain 39A
**230924_at**	TTLL6	tubulin tyrosine ligase-like family, member 6
**227388_at**	TUSC1	tumor suppressor candidate 1
**213423_x_at**	TUSC3	tumor suppressor candidate 3
**223325_at**	TXNDC11	thioredoxin domain containing 11
**201008_s_at**	TXNIP	thioredoxin interacting protein
**231904_at**	U2AF1	U2 small nuclear RNA auxiliary factor 1
**236715_x_at**	UACA	uveal autoantigen with coiled-coil domains and ankyrin repeats
**1294_at**	UBA7	ubiquitin-like modifier activating enzyme 7
**240383_at**	UBE2D3	ubiquitin-conjugating enzyme E2D 3 (UBC4/5 homolog, yeast)
**1557053_s_at**	UBE2G2	ubiquitin-conjugating enzyme E2G 2 (UBC7 homolog, yeast)
**222252_x_at**	UBQLN4	ubiquilin 4
**235327_x_at**	UBXN2A	UBX domain protein 2A
**203343_at**	UGDH	UDP-glucose 6-dehydrogenase
**232180_at**	UGP2	UDP-glucose pyrophosphorylase 2

**206094_x_at**	UGT1A1 /// UGT1A10 /// UGT1A3 /// UGT1A4 /// UGT1A5 /// UGT1A6 /// UGT1A7 /// UGT1A8 /// UGT1A9	UDP glucuronosyltransferase 1 family, polypeptide A1 /// UDP glucuronosyltransferase 1 family, polypeptide A10 /// UDP glucuronosyltransferase 1 family, polypeptide A3 /// UDP glucuronosyltransferase 1 family, polypeptide A4 /// UDP glucuronosyltransferase 1 family, polypeptide A5 /// UDP glucuronosyltransferase 1 family, polypeptide A6 /// UDP glucuronosyltransferase 1 family, polypeptide A7 /// UDP glucuronosyltransferase 1 family, polypeptide A8 /// UDP glucuronosyltransferase 1 family, polypeptide A9
**219948_x_at**	UGT2A3	UDP glucuronosyltransferase 2 family, polypeptide A3
**207392_x_at**	UGT2B15	UDP glucuronosyltransferase 2 family, polypeptide B15
**207245_at**	UGT2B17	UDP glucuronosyltransferase 2 family, polypeptide B17
**211682_x_at**	UGT2B28	UDP glucuronosyltransferase 2 family, polypeptide B28
**202893_at**	UNC13B	unc-13 homolog B (C. elegans)
**226899_at**	UNC5B	unc-5 homolog B (C. elegans)
**218190_s_at**	UQCR10	ubiquinol-cytochrome c reductase, complex III subunit X
**221173_at**	USH1C	Usher syndrome 1C (autosomal recessive, severe)
**233595_at**	USP34	ubiquitin specific peptidase 34
**237439_at**	USP43	ubiquitin specific peptidase 43
**223701_s_at**	USP47	ubiquitin specific peptidase 47
**220079_s_at**	USP48	ubiquitin specific peptidase 48
**208780_x_at**	VAPA	VAMP (vesicle-associated membrane protein)-associated protein A, 33kDa
**202205_at**	VASP	vasodilator-stimulated phosphoprotein
**204254_s_at**	VDR	vitamin D (1,25- dihydroxyvitamin D3) receptor
**209950_s_at**	VILL	villin-like
**236254_at**	VPS13B	vacuolar protein sorting 13 homolog B (yeast)
**212323_s_at**	VPS13D	vacuolar protein sorting 13 homolog D (S. cerevisiae)
**221704_s_at**	VPS37B	vacuolar protein sorting 37 homolog B (S. cerevisiae)
**218171_at**	VPS4B	vacuolar protein sorting 4 homolog B (S. cerevisiae)
**226485_at**	VSIG10	V-set and immunoglobulin domain containing 10
**228232_s_at**	VSIG2	V-set and immunoglobulin domain containing 2
**1552536_at**	VTI1A	vesicle transport through interaction with t-SNAREs homolog 1A (yeast)
**205011_at**	VWA5A	von Willebrand factor A domain containing 5A
**233929_x_at**	WASH3P	WAS protein family homolog 3 pseudogene
**240282_at**	WDR1	WD repeat domain 1
**229694_at**	WDR11	WD repeat domain 11
**212880_at**	WDR7	WD repeat domain 7
**238650_x_at**	WDR89	WD repeat domain 89
**203892_at**	WFDC2	WAP four-disulfide core domain 2
**224465_s_at**	WIBG	within bgcn homolog (Drosophila)
**229158_at**	WNK4	WNK lysine deficient protein kinase 4
**201294_s_at**	WSB1	WD repeat and SOCS box-containing 1
**213155_at**	WSCD1	WSC domain containing 1
**241994_at**	XDH	xanthine dehydrogenase
**209045_at**	XPNPEP1	X-prolyl aminopeptidase (aminopeptidase P) 1, soluble
**240168_at**	XPO7	exportin 7
**207598_x_at**	XRCC2	X-ray repair complementing defective repair in Chinese hamster cells 2
**227020_at**	YPEL2	yippee-like 2 (Drosophila)
**232516_x_at**	YY1AP1	YY1 associated protein 1
**1554239_s_at**	ZADH2	zinc binding alcohol dehydrogenase domain containing 2
**243648_at**	ZBED6	zinc finger, BED domain containing 6

**226554_at**	ZBTB7A	zinc finger and BTB domain containing 7A
**227782_at**	ZBTB7C	zinc finger and BTB domain containing 7C
**231899_at**	ZC3H12C	zinc finger CCCH-type containing 12C
**213063_at**	ZC3H14	zinc finger CCCH-type containing 14
**206169_x_at**	ZC3H7B	zinc finger CCCH-type containing 7B
**219062_s_at**	ZCCHC2	zinc finger, CCHC domain containing 2
**236243_at**	ZCCHC6	Zinc finger, CCHC domain containing 6
**230332_at**	ZCCHC7	Zinc finger, CCHC domain containing 7
**232440_at**	ZDHHC13	Zinc finger, DHHC-type containing 13
**222730_s_at**	ZDHHC2	zinc finger, DHHC-type containing 2
**218606_at**	ZDHHC7	zinc finger, DHHC-type containing 7
**222186_at**	ZFAND6	Zinc finger, AN1-type domain 6
**226137_at**	ZFHX3	zinc finger homeobox 3
**235728_at**	ZFP3	zinc finger protein 3 homolog (mouse)
**224631_at**	ZFP91	zinc finger protein 91 homolog (mouse)
**223387_at**	ZFYVE1	zinc finger, FYVE domain containing 1
**224445_s_at**	ZFYVE21	zinc finger, FYVE domain containing 21
**214142_at**	ZG16	zymogen granule protein 16 homolog (rat)
**1557384_at**	ZNF131	Zinc finger protein 131
**214715_x_at**	ZNF160	zinc finger protein 160
**231848_x_at**	ZNF207	zinc finger protein 207
**219603_s_at**	ZNF226	zinc finger protein 226
**233399_x_at**	ZNF252	Zinc finger protein 252
**1555192_at**	ZNF277	zinc finger protein 277
**228393_s_at**	ZNF302	zinc finger protein 302
**215359_x_at**	ZNF44	zinc finger protein 44
**1558486_at**	ZNF493	zinc finger protein 493
**244132_x_at**	ZNF518A	Zinc finger protein 518A
**208137_x_at**	ZNF611	zinc finger protein 611
**215978_x_at**	ZNF721	zinc finger protein 721
**235231_at**	ZNF789	zinc finger protein 789
**243618_s_at**	ZNF827	Zinc finger protein 827
**206059_at**	ZNF91	zinc finger protein 91
**223016_x_at**	ZRANB2	zinc finger, RAN-binding domain containing 2
**212601_at**	ZZEF1	zinc finger, ZZ-type with EF-hand domain 1
**226208_at**	ZSWIM6	zinc finger, SWIM-type containing 6

Supplementary Table 1. In tumorous epithelia, 2533 downregulated transcripts were identified which belong to 1509 known genes.
