# Supplementary material for: Genome-Wide Screening of Genes Regulated by DNA Methylation in Colon Cancer Development
Source: PLoS One. 2012 Oct 1;7(10):e46215. doi: 10.1371/journal.pone.0046215 (PMC3462205; doi:10.1371/journal.pone.0046215)
Supplement: Table S2 — Putative methylation regulated transcripts in LCM CRC samples. 154 common elements of 2533 downregulated transcripts in LCM tumor samples and 3000 upregulated transcripts from 5-Aza-treated HT29 cells. (PDF) [file pone.0046215.s006.pdf]

Supplementary Table S2

Putative methylation regulated transcripts in LCM CRC samples

Probe Set ID	Gene Symbol	Gene Title
**222688_at**	ACER3	alkaline ceramidase 3
**1557820_at**	AFG3L2	AFG3 ATPase family gene 3-like 2 (yeast)
**224667_x_at**	ANAPC16	anaphase promoting complex subunit 16
**202888_s_at**	ANPEP	alanyl (membrane) aminopeptidase
**200602_at**	APP	amyloid beta (A4) precursor protein
**1555962_at**	B3GNT7	UDP-GlcNAc:betaGal beta-1,3-N-acetylglucosaminyltransferase 7
**205263_at**	BCL10	B-cell CLL/lymphoma 10
**227736_at**	C10orf99	chromosome 10 open reading frame 99
**229520_s_at**	C14orf118	chromosome 14 open reading frame 118
**223484_at**	C15orf48	chromosome 15 open reading frame 48
**224458_at**	C9orf125	chromosome 9 open reading frame 125
**214315_x_at**	CALR	calreticulin
**208683_at**	CAPN2	calpain 2, (m/II) large subunit
**205789_at**	CD1D	CD1d molecule
**232266_x_at**	CDK13	Cyclin-dependent kinase 13
**236313_at**	CDKN2B	cyclin-dependent kinase inhibitor 2B (p15, inhibits CDK4)
**204159_at**	CDKN2C	cyclin-dependent kinase inhibitor 2C (p18, inhibits CDK4)
**205709_s_at**	CDS1	CDP-diacylglycerol synthase (phosphatidate cytidylyltransferase) 1
**200998_s_at**	CKAP4	cytoskeleton-associated protein 4
**220026_at**	CLCA4	chloride channel accessory 4
**241065_x_at**	CMAS	Cytidine monophosphate N-acetylneuraminic acid synthetase
**234981_x_at**	CMBL	carboxymethylenebutenolidase homolog (Pseudomonas)
**225009_at**	CMTM4	CKLF-like MARVEL transmembrane domain containing 4
**218900_at**	CNNM4	cyclin M4
**205927_s_at**	CTSE	cathepsin E
**244546_at**	CYCS	cytochrome c, somatic
**219290_x_at**	DAPP1	dual adaptor of phosphotyrosine and 3-phosphoinositides
**205370_x_at**	DBT	dihydrolipoamide branched chain transacylase E2
**242562_at**	DNAJC24	DnaJ (Hsp40) homolog, subfamily C, member 24
**227955_s_at**	EFNA5	ephrin-A5
**205222_at**	EHHADH	enoyl-CoA, hydratase/3-hydroxyacyl CoA dehydrogenase
**235645_at**	ESCO1	establishment of cohesion 1 homolog 1 (S. cerevisiae)
**242608_x_at**	FAM161B	Family with sequence similarity 161, member B
**226062_x_at**	FAM63A	family with sequence similarity 63, member A
**203698_s_at**	FRZB	frizzled-related protein
**211795_s_at**	FYB	FYN binding protein
**227405_s_at**	FZD8	frizzled homolog 8 (Drosophila)
**201722_s_at**	GALNT1	UDP-N-acetyl-alpha-D-galactosamine:polypeptide N-acetylgalactosaminyltransferase 1 (GalNAc-T1)
**206422_at**	GCG	glucagon
**212959_s_at**	GNPTAB	N-acetylglucosamine-1-phosphate transferase, alpha and beta subunits
**211040_x_at**	GTSE1	G-2 and S-phase expressed 1
**220071_x_at**	HAUS2	HAUS augmin-like complex, subunit 2
**217845_x_at**	HIGD1A	HIG1 hypoxia inducible domain family, member 1A
**200799_at**	HSPA1A	heat shock 70kDa protein 1A
**206502_s_at**	INSM1	insulinoma-associated 1

**202746_at**	ITM2A	integral membrane protein 2A
**1562063_x_at**	KIAA1245 /// LOC200030 /// NBPF1 /// NBPF10 /// NBPF11 /// NBPF8 /// NBPF9	KIAA1245 /// neuroblastoma breakpoint family, member 11-like /// neuroblastoma breakpoint family, member 1 /// neuroblastoma breakpoint family, member 10 /// neuroblastoma breakpoint family, member 11 /// neuroblastoma breakpoint family, member 8 /// neuroblastoma breakpoint family, member 9
**209211_at**	KLF5	Kruppel-like factor 5 (intestinal)
**213953_at**	KRT20	keratin 20
**200915_x_at**	KTN1	kinectin 1 (kinesin receptor)
**214709_s_at**	KTN1	kinectin 1 (kinesin receptor)
**235167_at**	LOC100190986	hypothetical LOC100190986
**232455_x_at**	LOC340085	hypothetical protein LOC340085
**242889_x_at**	LOC645431	hypothetical LOC645431
**204037_at**	LPAR1	lysophosphatidic acid receptor 1
**204674_at**	LRMP	lymphoid-restricted membrane protein
**220376_at**	LRRC19	leucine rich repeat containing 19
**211452_x_at**	LRRFIP1	leucine rich repeat (in FLII) interacting protein 1
**209579_s_at**	MBD4	methyl-CpG binding domain protein 4
**217109_at**	MUC4	mucin 4, cell surface associated
**226041_at**	NAPEPLD	N-acyl phosphatidylethanolamine phospholipase D
**232169_x_at**	NDUFS8	NADH dehydrogenase (ubiquinone) Fe-S protein 8, 23kDa (NADH-coenzyme Q reductase)
**202149_at**	NEDD9	neural precursor cell expressed, developmentally down-regulated 9
**201502_s_at**	NFKBIA	nuclear factor of kappa light polypeptide gene enhancer in B-cells inhibitor, alpha
**239748_x_at**	OCIAD1	OCIA domain containing 1
**206323_x_at**	OPHN1	oligophrenin 1
**1554384_at**	PADI2	peptidyl arginine deiminase, type II
**242871_at**	PAQR5	progestin and adipoQ receptor family member V
**204687_at**	PARM1	prostate androgen-regulated mucin-like protein 1
**206792_x_at**	PDE4C	phosphodiesterase 4C, cAMP-specific (phosphodiesterase E1 dunce homolog, Drosophila)
**203131_at**	PDGFRA	platelet-derived growth factor receptor, alpha polypeptide
**215179_x_at**	PGF	Placental growth factor
**226147_s_at**	PIGR	polymeric immunoglobulin receptor
**207109_at**	POU2F3	POU class 2 homeobox 3
**219392_x_at**	PRR11	proline rich 11
**232215_x_at**	PRR11	proline rich 11
**238513_at**	PRRG4	Proline rich Gla (G-carboxyglutamic acid) 4 (transmembrane)
**215894_at**	PTGDR	prostaglandin D2 receptor (DP)
**221872_at**	RARRES1	retinoic acid receptor responder (tazarotene induced) 1
**215588_x_at**	RIOK3	RIO kinase 3 (yeast)
**214041_x_at**	RPL37A	Ribosomal protein L37a
**230660_at**	SERTAD4	SERTA domain containing 4
**33323_r_at**	SFN	stratifin
**230375_at**	SFRS18	splicing factor, arginine/serine-rich 18
**218835_at**	SFTPA2	surfactant protein A2
**213936_x_at**	SFTPB	surfactant protein B
**223391_at**	SGPP1	sphingosine-1-phosphate phosphatase 1
**242578_x_at**	SLC22A3	Solute carrier family 22 (extraneuronal monoamine transporter), member 3
**224959_at**	SLC26A2	solute carrier family 26 (sulfate transporter), member 2

**226894_at**	SLC35A3	solute carrier family 35 (UDP-N-acetylglucosamine (UDP-GlcNAc) transporter), member A3
**220796_x_at**	SLC35E1	solute carrier family 35, member E1
**212569_at**	SMCHD1	structural maintenance of chromosomes flexible hinge domain containing 1
**232739_at**	SPIB	Spi-B transcription factor (Spi-1/PU.1 related)
**226353_at**	SPPL2A	signal peptide peptidase-like 2A
**202565_s_at**	SVIL	supervillin
**242093_at**	SYTL5	synaptotagmin-like 5
**236248_x_at**	TADA2B	transcriptional adaptor 2B
**212761_at**	TCF7L2	transcription factor 7-like 2 (T-cell specific, HMG-box)
**229341_at**	TFCP2L1	transcription factor CP2-like 1
**242377_x_at**	THUMPD3	THUMP domain containing 3
**205812_s_at**	TMED9	transmembrane emp24 protein transport domain containing 9
**240770_at**	TMEM171	transmembrane protein 171
**213285_at**	TMEM30B	transmembrane protein 30B
**219736_at**	TRIM36	tripartite motif-containing 36
**236715_x_at**	UACA	uveal autoantigen with coiled-coil domains and ankyrin repeats
**222252_x_at**	UBQLN4	ubiquilin 4
**235327_x_at**	UBXN2A	UBX domain protein 2A
**207245_at**	UGT2B17	UDP glucuronosyltransferase 2 family, polypeptide B17
**233595_at**	USP34	ubiquitin specific peptidase 34
**218171_at**	VPS4B	vacuolar protein sorting 4 homolog B (S. cerevisiae)
**232516_x_at**	YY1AP1	YY1 associated protein 1
**233399_x_at**	ZNF252	Zinc finger protein 252
**208137_x_at**	ZNF611	zinc finger protein 611
**215978_x_at**	ZNF721	zinc finger protein 721
**AFFX-hum_alu_at**	---	---
**1557222_at**	---	---
**1557780_at**	---	---
**1566887_x_at**	---	---
**1569409_x_at**	---	---
**207730_x_at**	---	---
**208246_x_at**	---	---
**210679_x_at**	---	---
**214989_x_at**	---	---
**215604_x_at**	---	---
**215628_x_at**	---	---
**216147_at**	---	---
**216187_x_at**	---	---
**217679_x_at**	---	---
**227682_at**	---	---
**229157_at**	---	---
**229434_at**	---	---
**230324_at**	---	---
**230387_at**	---	---
**232614_at**	---	---
**233017_x_at**	---	---
**233041_x_at**	---	---
**233270_x_at**	---	---
**233427_x_at**	---	---

**234137_s_at**	---	---
**235084_x_at**	---	---
**235757_at**	---	---
**236170_x_at**	---	---
**236251_at**	---	---
**236947_at**	---	---
**237868_x_at**	---	---
**240326_at**	---	---
**240612_at**	---	---
**241303_x_at**	---	---
**241956_at**	---	---
**242235_x_at**	---	---
**242398_x_at**	---	---
**243147_x_at**	---	---
**243931_at**	---	---
**244384_at**	---	---

Supplementary Table S2. 154 common elements of 2533 downregulated transcripts in LCM tumor samples and 3000 upregulated transcripts from 5-Aza-treated HT29 cells.
