# Supplementary material for: Genome-Wide Screening of Genes Regulated by DNA Methylation in Colon Cancer Development
Source: PLoS One. 2012 Oct 1;7(10):e46215. doi: 10.1371/journal.pone.0046215 (PMC3462205; doi:10.1371/journal.pone.0046215)
Supplement: Table S3 — Putative methylation regulated transcripts downregulated in both adenoma and CRC samples. From the 154 methylation related transcripts (listed in Supplementary Table S2.), 108 were also downregulated in adenoma samples. These transcripts were found to be inactivated by DNA methylation at the early phase of carcinogenesis. Supplemetary Figure S1 shows the expression pattern of these genes in normal, adenoma and tumor samples. (PDF) [file pone.0046215.s007.pdf]

Supplementary Table S3

Putative methylation regulated transcripts downregulated in both adenoma and CRC samples

Probe Set ID	Gene Symbol	Gene Title
**222688_at**	ACER3	alkaline ceramidase 3
**224667_x_at**	ANAPC16	anaphase promoting complex subunit 16
**202888_s_at**	ANPEP	alanyl (membrane) aminopeptidase
**1555962_at**	B3GNT7	UDP-GlcNAc:betaGal beta-1,3-N-acetylglucosaminyltransferase 7
**205263_at**	BCL10	B-cell CLL/lymphoma 10
**229520_s_at**	C14orf118	chromosome 14 open reading frame 118
**223484_at**	C15orf48	chromosome 15 open reading frame 48
**224458_at**	C9orf125	chromosome 9 open reading frame 125
**214315_x_at**	CALR	calreticulin
**232266_x_at**	CDK13	Cyclin-dependent kinase 13
**204159_at**	CDKN2C	cyclin-dependent kinase inhibitor 2C (p18, inhibits CDK4)
**205709_s_at**	CDS1	CDP-diacylglycerol synthase (phosphatidate cytidylyltransferase) 1
**200998_s_at**	CKAP4	cytoskeleton-associated protein 4
**220026_at**	CLCA4	chloride channel accessory 4
**234981_x_at**	CMBL	carboxymethylenebutenolidase homolog (Pseudomonas)
**225009_at**	CMTM4	CKLF-like MARVEL transmembrane domain containing 4
**218900_at**	CNNM4	cyclin M4
**244546_at**	CYCS	cytochrome c, somatic
**219290_x_at**	DAPP1	dual adaptor of phosphotyrosine and 3-phosphoinositides
**205370_x_at**	DBT	dihydrolipoamide branched chain transacylase E2
**242562_at**	DNAJC24	DnaJ (Hsp40) homolog, subfamily C, member 24
**227955_s_at**	EFNA5	ephrin-A5
**242608_x_at**	FAM161B	Family with sequence similarity 161, member B
**226062_x_at**	FAM63A	family with sequence similarity 63, member A
**201722_s_at**	GALNT1	UDP-N-acetyl-alpha-D-galactosamine:polypeptide N-acetylgalactosaminyltransferase 1 (GalNAc-T1)
**206422_at**	GCG	glucagon
**212959_s_at**	GNPTAB	N-acetylglucosamine-1-phosphate transferase, alpha and beta subunits
**211040_x_at**	GTSE1	G-2 and S-phase expressed 1
**220071_x_at**	HAUS2	HAUS augmin-like complex, subunit 2
**217845_x_at**	HIGD1A	HIG1 hypoxia inducible domain family, member 1A
**200799_at**	HSPA1A	heat shock 70kDa protein 1A
**206502_s_at**	INSM1	insulinoma-associated 1
**202746_at**	ITM2A	integral membrane protein 2A
**1562063_x_at**	KIAA1245 /// LOC200030 /// NBPF1 /// NBPF10 /// NBPF11 /// NBPF8 /// NBPF9	KIAA1245 /// neuroblastoma breakpoint family, member 11-like /// neuroblastoma breakpoint family, member 1 /// neuroblastoma breakpoint family, member 10 /// neuroblastoma breakpoint family, member 11 /// neuroblastoma breakpoint family, member 8 /// neuroblastoma breakpoint family, member 9
**235167_at**	LOC100190986	hypothetical LOC100190986
**204037_at**	LPAR1	lysophosphatidic acid receptor 1
**204674_at**	LRMP	lymphoid-restricted membrane protein
**220376_at**	LRRC19	leucine rich repeat containing 19
**211452_x_at**	LRRFIP1	leucine rich repeat (in FLII) interacting protein 1
**209579_s_at**	MBD4	methyl-CpG binding domain protein 4
**217109_at**	MUC4	mucin 4, cell surface associated

**232169_x_at**	NDUFS8	NADH dehydrogenase (ubiquinone) Fe-S protein 8, 23kDa (NADH-coenzyme Q reductase)
**201502_s_at**	NFKBIA	nuclear factor of kappa light polypeptide gene enhancer in B-cells inhibitor, alpha
**239748_x_at**	OCIAD1	OCIA domain containing 1
**206323_x_at**	OPHN1	oligophrenin 1
**1554384_at**	PADI2	peptidyl arginine deiminase, type II
**242871_at**	PAQR5	progestin and adipoQ receptor family member V
**204687_at**	PARM1	prostate androgen-regulated mucin-like protein 1
**206792_x_at**	PDE4C	phosphodiesterase 4C, cAMP-specific (phosphodiesterase E1 dunce homolog, Drosophila)
**203131_at**	PDGFRA	platelet-derived growth factor receptor, alpha polypeptide
**215179_x_at**	PGF	Placental growth factor
**207109_at**	POU2F3	POU class 2 homeobox 3
**219392_x_at**	PRR11	proline rich 11
**232215_x_at**	PRR11	proline rich 11
**238513_at**	PRRG4	Proline rich Gla (G-carboxyglutamic acid) 4 (transmembrane)
**215894_at**	PTGDR	prostaglandin D2 receptor (DP)
**221872_at**	RARRES1	retinoic acid receptor responder (tazarotene induced) 1
**215588_x_at**	RIOK3	RIO kinase 3 (yeast)
**214041_x_at**	RPL37A	Ribosomal protein L37a
**33323_r_at**	SFN	stratifin
**230375_at**	SFRS18	splicing factor, arginine/serine-rich 18
**218835_at**	SFTPA2	surfactant protein A2
**213936_x_at**	SFTPB	surfactant protein B
**242578_x_at**	SLC22A3	Solute carrier family 22 (extraneuronal monoamine transporter), member 3
**220796_x_at**	SLC35E1	solute carrier family 35, member E1
**232739_at**	SPIB	Spi-B transcription factor (Spi-1/PU.1 related)
**202565_s_at**	SVIL	supervillin
**236248_x_at**	TADA2B	transcriptional adaptor 2B
**212761_at**	TCF7L2	transcription factor 7-like 2 (T-cell specific, HMG-box)
**229341_at**	TFCP2L1	transcription factor CP2-like 1
**242377_x_at**	THUMPD3	THUMP domain containing 3
**205812_s_at**	TMED9	transmembrane emp24 protein transport domain containing 9
**240770_at**	TMEM171	transmembrane protein 171
**219736_at**	TRIM36	tripartite motif-containing 36
**236715_x_at**	UACA	uveal autoantigen with coiled-coil domains and ankyrin repeats
**235327_x_at**	UBXN2A	UBX domain protein 2A
**207245_at**	UGT2B17	UDP glucuronosyltransferase 2 family, polypeptide B17
**233595_at**	USP34	ubiquitin specific peptidase 34
**218171_at**	VPS4B	vacuolar protein sorting 4 homolog B (S. cerevisiae)
**232516_x_at**	YY1AP1	YY1 associated protein 1
**233399_x_at**	ZNF252	Zinc finger protein 252
**208137_x_at**	ZNF611	zinc finger protein 611
**215978_x_at**	ZNF721	zinc finger protein 721
**1566887_x_at**	---	---
**1569409_x_at**	---	---
**207730_x_at**	---	---
**208246_x_at**	---	---
**210679_x_at**	---	---
**214989_x_at**	---	---

**215604_x_at**	---	---
**215628_x_at**	---	---
**216147_at**	---	---
**216187_x_at**	---	---
**217679_x_at**	---	---
**229157_at**	---	---
**229434_at**	---	---
**233041_x_at**	---	---
**233427_x_at**	---	---
**235084_x_at**	---	---
**235757_at**	---	---
**237868_x_at**	---	---
**240612_at**	---	---
**241303_x_at**	---	---
**242235_x_at**	---	---
**242398_x_at**	---	---
**243147_x_at**	---	---
**243931_at**	---	---
**244384_at**	---	---

Supplementary Table S3. From the 154 methylation related transcripts (listed in Supplementary Table S2.), 108 were also downregulated in adenoma samples. These transcripts were found to be inactivated by DNA methylation at the early phase of carcinogenesis. Supplemetary Figure S1 shows the expression pattern of these genes in normal, adenoma and tumor samples.
