# Supplementary material for: Genome-Wide Screening of Genes Regulated by DNA Methylation in Colon Cancer Development
Source: PLoS One. 2012 Oct 1;7(10):e46215. doi: 10.1371/journal.pone.0046215 (PMC3462205; doi:10.1371/journal.pone.0046215)
Supplement: Table S4 — Applied primer sequences. The table shows the primer sequences used for RT-PCR validation of mRNA levels of six genes (top) and for bisulphite sequencing on PTGDR promoter region sequence (chr.14∶52734410-52734668) (bottom). (PDF) [file pone.0046215.s008.pdf]

Supplementary Table S4

Applied primer sequences

Affy_ID	Gene symbol	Forward primer sequence 5'-3'	Reverse primer sequence 5'-3'
**242608_x_at**	FAM161B	GAGCAAGACCCCATCTCAAA	CAATCATCCTGTCTCCACAGC
**204674_at**	LRMP	TGTTTGCAGCTTTGATGAGC	TGAGTCCTCTTGCTGTGTGG
**206422_at**	GCG	TCCGATCTGACATATCTGCATT	CACCACTGTGGCTACCAGTTC
**223484_at**	C15orf48 (NMES1)	TGCAGCAATAACTGCACTGTC	GCTTTGTAAATTTTAAGAAAAACATTC
**236313_at**	CDKN2B	TTTGAAGGATACATGCAAAACA	CCACAATGGAGCTAGAAGCA
**215894_at**	PTGDR	TTCAGATCTCCAGTATTTCGGATA	CACCGGCTCCTGTACCTAAG
	PTGDR_regio2	ATTATTTTTGTGGAAAAAGGTAATT	CCCTAAAAAAAACCAACACTCCAATAC

Supplementary Table S4. The table shows the primer sequences used for RT-PCR validation of mRNA levels of six genes (top) and for bisulphite sequencing on PTGDR promoter region sequence (chr.14: 52734410-52734668) (bottom).
